# Seasonal Influenza Vaccination for Children in Thailand: A Cost-Effectiveness Analysis

**DOI:** 10.1371/journal.pmed.1001829

**Published:** 2015-05-26

**Authors:** Aronrag Meeyai, Naiyana Praditsitthikorn, Surachai Kotirum, Wantanee Kulpeng, Weerasak Putthasri, Ben S. Cooper, Yot Teerawattananon

**Affiliations:** 1 Health Intervention and Technology Assessment Program, Nonthaburi, Thailand; 2 Department of Epidemiology, Faculty of Public Health, Mahidol University, Bangkok, Thailand; 3 Bureau of AIDS, TB, and STI, Department of Disease Control, Ministry of Public Health, Nonthaburi, Thailand; 4 International Health Policy Program, Ministry of Public Health, Nonthaburi, Thailand; 5 Mahidol Oxford Tropical Medicine Research Unit, Bangkok, Thailand; 6 Centre for Tropical Medicine and Global Health, Nuffield Department of Clinical Medicine, University of Oxford, Oxford, United Kingdom; George Washington University, UNITED STATES

## Abstract

**Background:**

Seasonal influenza is a major cause of mortality worldwide. Routine immunization of children has the potential to reduce this mortality through both direct and indirect protection, but has not been adopted by any low- or middle-income countries. We developed a framework to evaluate the cost-effectiveness of influenza vaccination policies in developing countries and used it to consider annual vaccination of school- and preschool-aged children with either trivalent inactivated influenza vaccine (TIV) or trivalent live-attenuated influenza vaccine (LAIV) in Thailand. We also compared these approaches with a policy of expanding TIV coverage in the elderly.

**Methods and Findings:**

We developed an age-structured model to evaluate the cost-effectiveness of eight vaccination policies parameterized using country-level data from Thailand. For policies using LAIV, we considered five different age groups of children to vaccinate. We adopted a Bayesian evidence-synthesis framework, expressing uncertainty in parameters through probability distributions derived by fitting the model to prospectively collected laboratory-confirmed influenza data from 2005-2009, by meta-analysis of clinical trial data, and by using prior probability distributions derived from literature review and elicitation of expert opinion. We performed sensitivity analyses using alternative assumptions about prior immunity, contact patterns between age groups, the proportion of infections that are symptomatic, cost per unit vaccine, and vaccine effectiveness. Vaccination of children with LAIV was found to be highly cost-effective, with incremental cost-effectiveness ratios between about 2,000 and 5,000 international dollars per disability-adjusted life year averted, and was consistently preferred to TIV-based policies. These findings were robust to extensive sensitivity analyses. The optimal age group to vaccinate with LAIV, however, was sensitive both to the willingness to pay for health benefits and to assumptions about contact patterns between age groups.

**Conclusions:**

Vaccinating school-aged children with LAIV is likely to be cost-effective in Thailand in the short term, though the long-term consequences of such a policy cannot be reliably predicted given current knowledge of influenza epidemiology and immunology. Our work provides a coherent framework that can be used for similar analyses in other low- and middle-income countries.

## Abstract 2

Ben Cooper and colleagues use an age-structured model to estimate optimal cost-effectiveness of flu vaccination among Thai children aged 2 to 17.

## Editors' Summary

**Background:**

Every year, millions of people catch influenza, a viral disease of the airways. Most infected individuals recover quickly, but elderly people, the very young, and chronically ill individuals are at high risk of developing serious complications such as pneumonia; seasonal influenza kills about half a million people annually. Small but frequent changes in the influenza virus mean that an immune response produced one year by exposure to the virus provides only partial protection against influenza the next year. Annual immunization with a vaccine that contains killed or live-attenuated (weakened) influenza viruses of the major circulating strains can reduce a person’s chance of catching influenza. Consequently, many countries run seasonal influenza vaccination programs that target elderly people and other people at high risk of influenza complications, and people who care for these individuals.

**Why Was This Study Done?:**

As well as reducing the vaccinated person’s risk of infection, influenza vaccination protects unvaccinated members of the population by reducing the chances of influenza spreading. Because children make a disproportionately large contribution to the transmission of influenza, vaccination of children might therefore provide greater benefits to the whole population than vaccination of elderly people, particularly when vaccination uptake among the elderly is low. Thus, many high-income countries now recommend annual influenza vaccination of children with a trivalent live-attenuated influenza vaccine (LAIV; a trivalent vaccine contains three viruses), which is sprayed into the nose. However, to date no low- or middle-income countries have evaluated this policy. Here, the researchers develop a mathematical model (framework) to evaluate the cost-effectiveness of annual vaccination of children with LAIV or trivalent inactivated influenza vaccine (TIV) in Thailand. A cost-effectiveness analysis evaluates whether a medical intervention is good value for money by comparing the health outcomes and costs associated with the introduction of the intervention with the health outcomes and costs of the existing standard of care. Thailand, a middle-income country, offers everyone over 65 years old free seasonal influenza vaccination with TIV, but vaccine coverage in this age group is low (10%).

**What Did the Researchers Do and Find?:**

The researchers developed a modeling framework that contained six connected components including a transmission model that incorporated infectious contacts within and between different age groups, a health outcome model that calculated the disability-adjusted life years (DALYs, a measure of the overall disease burden) averted by specific vaccination policies, and a cost model that calculated the costs to the population of each policy. They used this framework and data from Thailand to calculate the cost-effectiveness of six childhood vaccination policies in Thailand (one with TIV and five with LAIV that targeted children of different ages) against a baseline policy of 10% TIV coverage in the elderly; they also investigated the cost-effectiveness of increasing vaccination in the elderly to 66%. All seven vaccination policies tested reduced influenza cases and deaths compared to the baseline policy, but the LAIV-based polices were consistently better than the TIV-based policies; the smallest reductions were seen when TIV coverage in elderly people was increased to 66%. All seven policies were highly cost-effective according to the World Health Organization’s threshold for cost-effectiveness. That is, the cost per DALY averted by each policy compared to the baseline policy (the incremental cost-effectiveness ratio) was less than Thailand’s gross domestic product per capita (the total economic output of a country divided by the number of people in the country).

**What Do These Findings Mean?:**

These findings suggest that seasonal influenza vaccination of children with LAIV is likely to represent good value for money in Thailand and, potentially, in other middle- and low-income countries in the short term. The long-term consequences of annual influenza vaccination of children in Thailand cannot be reliably predicted, however, because of limitations in our current understanding of influenza immunity in populations. Moreover, the accuracy of these findings is limited by the assumptions built into the modeling framework, including the vaccine costs and efficacy that were used to run the model, which were estimated from limited data. Importantly, however, these findings support proposals for large-scale community-based controlled trials of policies to vaccinate children against influenza in low- and middle-income countries. Indeed, based on these findings, Thailand is planning to evaluate school-based seasonal influenza vaccination in a few provinces in 2016 before considering a nationwide program of seasonal influenza vaccination of children.

**Additional Information:**

This list of resources contains links that can be accessed when viewing the PDF on a device or via the online version of the article at http://dx.doi.org/10.1371/journal.pmed.1001829.

- The UK National Health Service Choices website provides information for patients about seasonal influenza, about influenza vaccination, and about influenza vaccination in children
- The World Health Organization provides information on seasonal influenza (in several languages) and on influenza vaccines
- The US Centers for Disease Control and Prevention also provides information for patients and health professionals on all aspects of seasonal influenza, including information about vaccination, about children, influenza, and vaccination, and about herd immunity; its website contains a short video about personal experiences of influenza
- Flu.gov, a US government website, provides access to information on seasonal influenza and vaccination
- MedlinePlus has links to further information about influenza and about vaccination (in English and Spanish)
- The Thai National Influenza Center monitors influenza activity throughout Thailand

## Introduction

Vaccination against influenza has the potential to substantially reduce mortality and morbidity both through direct protection (reduced risk of infection in those receiving the vaccine) and through indirect protection (reduced risk of infection in those not receiving the vaccine) [1,2]. Because children have a high burden of influenza-related illness and make a large contribution to influenza transmission in the wider community, it has been proposed that preferentially vaccinating this age group would represent efficient use of vaccine in high-income countries in the temperate climate zone [3–7]. The experience in Japan, where children were routinely immunized against influenza between 1962 and 1987, suggests that indirect benefits could be large [8]. Moreover, in settings where access to health care is limited, or where voluntary uptake of influenza vaccination in high-risk populations has been low, vaccinating school-aged children could represent a pragmatic and effective intervention to reduce influenza morbidity and mortality in the whole community. A further advantage of targeting this age group is that vaccine effectiveness may be greater in children than in elderly high-risk age groups [3]. Such a policy may be particularly relevant for Thailand, where despite free provision of seasonal influenza vaccine to all Thai nationals aged 65 y and above since 2008, annual vaccine coverage in this age group has remained in the region of 10% [9].

Two types of influenza vaccine are licensed for use in children: trivalent inactivated influenza vaccine (TIV) and trivalent live-attenuated influenza vaccine (LAIV). We consider both in our analysis, though the primary focus is on LAIV, which has been found to be more effective in children [10]. TIV is administered intramuscularly and is recommended for those aged at least 6 mo. LAIV is administered intranasally and is recommended only for those between the ages of 2 and 49 y. In both cases, one dose each year is recommended, except for children under 9 y who were not vaccinated the previous influenza season, for whom two doses at least 4 wk apart are recommended [10]. Antibodies indicating influenza protection emerge about 2 wk after vaccination.

Currently, however, no low- or middle-income country has adopted or, to our knowledge, formally evaluated a childhood influenza vaccination policy. We aimed to evaluate whether such a policy could be cost-effective in a tropical middle-income country, using detailed epidemiological and cost data from Thailand to inform our analysis. Our work also provides a coherent framework (and computer code) that can be used to allow the analysis to be repeated in other low- and middle-income countries.

When evaluating costs and health outcomes associated with vaccination, both direct and indirect effects should be accounted for [1]. Our approach does this using an age-structured dynamic transmission model. We employ a Bayesian approach, representing uncertainty in model parameters through probability distributions chosen to represent current knowledge and beliefs [11]. This allows us to combine information from multiple sources within a unified framework, while ensuring appropriate propagation of uncertainty through the model and accounting for correlations between parameter values [2]. The model thus combines analysis of prospective epidemiological data, meta-analysis of clinical trial data, estimates of life years lost due to influenza infections, and prior probability distributions derived from literature review and formal elicitation of expert opinion. We report both the epidemiological and the economic outcomes of the model.

## Methods

We compared seven vaccination policies against a baseline policy (policy 0) of no additional vaccination (Table 1). Policies 1–6 involved annual vaccination of children in specified age ranges: 2–11 y for policies 1 and 2, and 2–17 y, 2–5 y, 6–11 y, and 12–17 y, respectively, for polices 3–6. In policies 2–6, children were vaccinated with LAIV. Policy 1 differed from policy 2 only in the use of TIV instead of LAIV. Age groups were chosen to reflect the three stages in the Thai education system (3–5 y, kindergarten; 6–11 y, elementary school; 12–17 y, secondary school). For the youngest age group we extended the age range to 2 y, the minimum age at which LAIV can be used. For comparison we also considered a policy of expanding annual coverage levels with TIV to 66% in those aged at least 60 y (policy 7). In all other scenarios (including policy 0), 10% of those aged 60 y and over were assumed to receive TIV annually. For a given value of willingness to pay per disability-adjusted life year (DALY) averted (the cost-effectiveness threshold), we consider the optimal policy to be the one with the highest expected incremental net benefit (INB). The INB is the difference between the monetary value of health gains (the product of the number of DALYs averted by the policy and the cost-effectiveness threshold) and the costs of these health gains.

**Table 1:** Policies modeled.

Policy Number	Policy Description
0	No additional vaccine
1	Vaccinate those aged 2–11 y with TIV
2	Vaccinate those aged 2–11 y with LAIV
3	Vaccinate those aged 2–17 y with LAIV
4	Vaccinate those aged 2–5 y with LAIV
5	Vaccinate those aged 6–11 y with LAIV.
6	Vaccinate those aged 12–17 y with LAIV
7	Increase vaccine coverage for those aged ≥60 y with TIV

We evaluated the sensitivity of our results to alternative assumptions about (i) mixing patterns between age groups, (ii) vaccine effectiveness, (iii) baseline immunity (prior to vaccination), (iv) vaccine coverage, (v) influenza transmissibility in Thailand, (vi) vaccine costs, and (vii) the probability that an influenza infection is symptomatic (Table 2).

**Table 2:** Analyses and assumptions.

Analysis	Description/Assumptions
All scenarios	•Vaccinate those aged ≥60 y with TIV at 10% coverage in policies 0–6
Base case analysis	•Contact matrix describing interactions between age groups derived from all contacts
	•66% vaccine coverage in target age groups (except for those aged ≥60 y for policies 0–6)
	•Vaccine effectiveness derived from meta-analysis of trials in target age groups
	•Prior immunity levels derived from elicitation exercise
	•Flat priors for virus reproduction numbers
Sensitivity analysis 1	Contact matrix derived from physical contacts only
Sensitivity analysis 2	The prior age-specific probability of immunity to a given influenza subtype at the start of each influenza season is double that assumed in the base case analysis
Sensitivity analysis 3	VE estimates for LAIV are based on a meta-analysis of trials conducted mostly in Asia, leading to lower estimates compared to the base case assumption
Sensitivity analysis 4	Vaccine coverage assumed to be 50% in target age groups instead of 66%
Sensitivity analysis 5	Prior distributions for the basic reproduction numbers of different influenza subtypes are chosen to approximate posteriors from an analysis of seasonal influenza in a temperate country [2]
Sensitivity analysis 6	Prior distribution for the age-specific probability of immunity at the start of each influenza season approximates corresponding posterior distributions from England and Wales [2]
Sensitivity analysis 7	In those aged 2–12 y, probability of immunity at the start of each influenza season is three times higher than in the base case analysis
Sensitivity analysis 8	Probability that influenza infection is symptomatic taken to be one-quarter of the value in the baseline analysis (~17% instead of ~68%)

VE, vaccine efficacy.

### Modeling Framework

**Fig 1:**
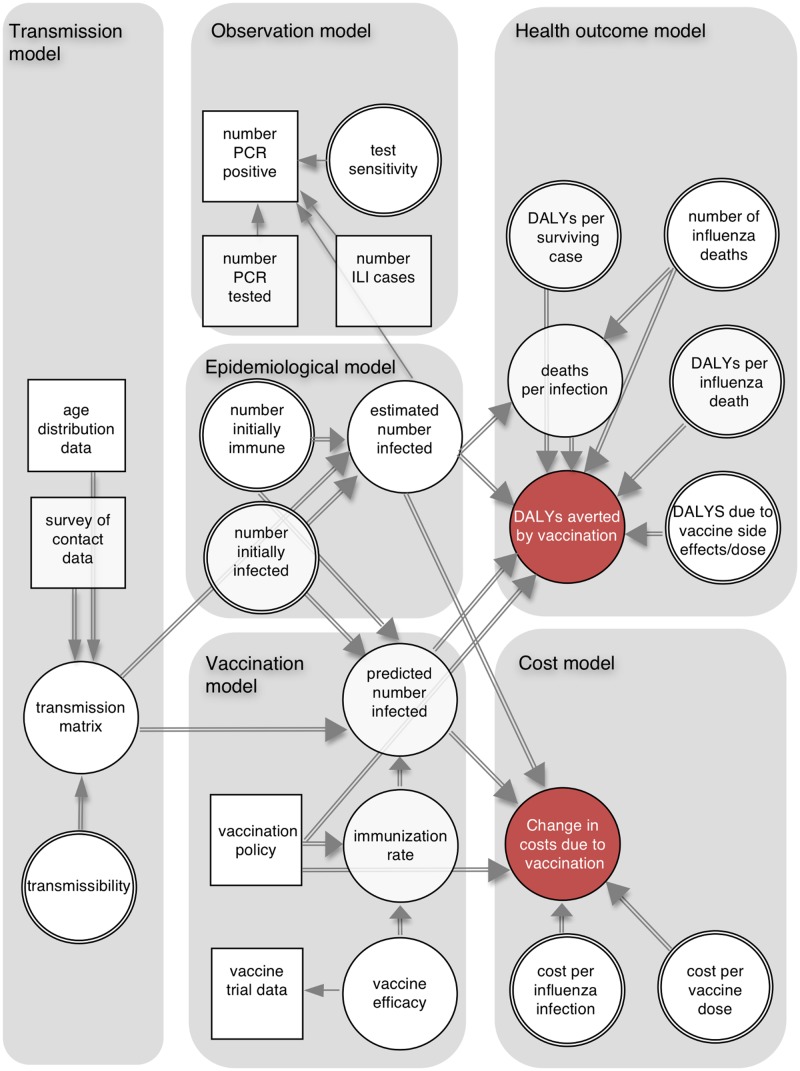
Schematic illustration of analytic framework. The directed acyclic graph illustrates the dependencies between model components. Squares represent data sources, and circles represent quantities about which we are uncertain (double circles indicate quantities for which we have external information about their values, which is represented by informative prior distributions). Single and double arrows indicate stochastic and deterministic relationships, respectively, and arrows point towards the dependent variable. ILI, influenza-like illness.

The cost-effectiveness analysis combined six connected components (Fig 1): a dynamic transmission model, which defined infectious contacts within and between different age groups; an epidemiological model, which used the transmission model to specify the number infected in each age group at each time point; an observation model, which related the output of the epidemiological model to influenza surveillance data; a vaccination model, which combined meta-analysis of vaccine trial data with the transmission model and vaccination scenarios; a health outcome model, which calculated the number of DALYs averted by each vaccination policy; and a cost model, which calculated costs from a societal perspective associated with the different policies.

Using this framework we calculated the following: (i) the probability distributions of the number of infections, DALYs averted, and deaths averted by each policy; (ii) the probability that each policy had the highest INB as a function of the cost-effectiveness threshold—this is shown by the cost-effectiveness acceptability curves (CEACs) [12]; (iii) the policy with the highest expected INB as a function of the cost-effectiveness threshold—the probability that a policy has the highest INB for a given threshold is shown on the cost-effectiveness acceptability frontier (CEAF) [12]; (iv) the expected value of perfect information (EVPI), which measures the expected monetary value of improved decision-making that would result from perfect information about model parameters [12]; (v) the expected value of partial perfect information (EVPPI), which is similar to EVPI but assumes perfect information about only one parameter at a time [13]; and (vi) the optimal policy as a function of vaccine effectiveness, vaccine cost, and the cost-effectiveness threshold (a threshold analysis).

Probability distributions for model parameters were obtained by combining prior distributions (derived from previous research and formal elicitation of expert opinion) with the likelihood of the observed data to obtain posterior distributions. All outcomes of interest can be expressed as summaries of functions of these posterior distributions. We also performed eight sensitivity analyses where we used alternative prior distributions or varied other modeling assumptions (Table 2).

### Details of Model Components

#### Transmission model

The transmission model was implemented as a system of coupled ordinary differential equations. For each of the three influenza subtypes (influenza A/H1N1, A/H3N2, and B), individuals within each of six age groups (<2 y, 2–5 y, 6–11 y, 12–17 y, 18–59 y, and ≥60 y) were partitioned into four compartments: susceptible and able to be infected with a circulating influenza subtypes; latently infected, but not yet infectious; currently infectious; and immune (not able to be infected with the same influenza subtype). Immunity may arise either through natural infection or effective vaccination. Potentially infectious contacts between age groups are accounted for using a matrix of contact patterns (the “mixing matrix”) empirically derived from a diary-based survey. In the base case analysis, we derived this matrix using all contacts (physical skin-to-skin contacts and nonphysical two-way spoken exchanges between people at the same location). In sensitivity analysis 1, we used only physical contacts.

#### Epidemiological model

We defined each influenza season to run from April 1 of a given year to March 31 of the following year. We assumed that at the start of each influenza season a proportion of people in each age group were immune to circulating influenza subtypes, and that a small proportion were already infected. Both proportions were age-dependent and influenza-subtype-specific, and uncertainty in their values was expressed in informative prior distributions, derived from an elicitation exercise (see below). Sensitivity analyses 2 and 6 made alternative immunity assumptions. Within each influenza season, we assumed that once individuals were infected with one influenza subtype, they remained immune to that subtype (i.e., were not able to be reinfected) but that immunity to other subtypes was not affected. Prior distribution assumptions for model parameters are given in Table 3.

**Table 3:** Prior distributions for epidemiology model used in the base case analysis.

Parameter	Prior Distribution; Mean (95% CrI)	Notes/Source for Prior
**Basic reproduction number (R0)**	Uniform [0.1, 5]^a^; 2.55 (0.22, 4.88)	Uninformative over plausible range
**Serial interval**	Shifted gamma^b^; 2.50 (2.31, 2.70)	[14]
**Probability of immunity at the start of influenza season**		
<2 y	Beta; 0.10 (0.05, 0.17)	Elicitation exercise
2–5 y	Beta; 0.13 (0.06, 0.21)	Elicitation exercise
6–11 y	Beta; 0.17 (0.14, 0.19)	Elicitation exercise
12–17 y	Beta; 0.17 (0.14, 0.19)	Elicitation exercise
18–59 y	Beta; 0.19 (0.13, 0.26)	Elicitation exercise
≥60 y	Beta; 0.19 (0.10, 0.30)	Elicitation exercise
**Probability of any symptoms given infection**	Beta; 0.67 (0.58, 0.74)	[15]
**Initial proportion in each age group infected**	Beta; 0.005 (<0.001, 0.03)	Elicitation exercise
**Sensitivity of laboratory confirmation test**	Beta; 0.5 (0.2, 0.8)	Weakly informative over plausible range

^a^Assigns equal probabilities to all values between 0.1 and 5, which includes the entire range of values with non-negligible probabilities from previous studies [2].

^b^The serial interval is the sum of the latent period (which has an expected value of 1 d) and the infectious period (which has a gamma-distributed prior with mean 1.5 and standard deviation 0.1).

CrI, credible interval.

#### Observation model

Model fitting was based on 4 y of data (April 2005–March 2009) derived from the national influenza surveillance network in Thailand. These data combine laboratory-confirmed cases of infection with influenza A/H1N1, A/H3N2, and B collected prospectively from sentinel sites throughout Thailand with national data on outpatients presenting with influenza-like illness (ILI) and inpatients with influenza pneumonia (IP) symptoms [16].

The laboratory-confirmed cases each month were assumed to follow a multinomial distribution, where the probability of a test yielding a positive result for each influenza subtype depended on the predicted number of infections with each influenza sybtype, the probability that an influenza infection in a given age group was reported as an ILI case, the test sensitivity, and the proportion of ILI cases caused by the given influenza subtype.

It was assumed that some age-specific proportion of people with influenza infections seek medical attention and are recorded as cases of ILI. We also assumed that the number of ILI and IP cases not due to influenza varies with season and age group. We took the minimum monthly number of ILI and IP cases for the given season and age group as an estimate of this quantity. A small (and known) number of people presenting with ILI are tested for virus positivity and classified either as infected with influenza A/H1N1, influenza A/H3N2, or influenza B, or as having no detectable influenza infection [14]. We assumed imperfect sensitivity for these tests, but perfect specificity.

#### Vaccination model

The vaccination model included a meta-analysis to determine vaccine efficacy (VE) in children and to derive a predictive distribution of vaccine effectiveness. In our base case analysis, we used data from randomized controlled trials of TIV and LAIV satisfying previously specified inclusion criteria [10], with the additional requirement that the median age of participants was between 2 and 15 y. These criteria required laboratory-confirmed influenza infection to be the primary end point, vaccine administration to be in accordance with recommendations, and the control group to receive a placebo or a vaccine other than influenza [10]. Trials meeting these criteria were sought in recent systematic reviews and through PubMed searches [10,17,18].

We defined VE as one minus the ratio of the probability of influenza infection if vaccinated to the probability of influenza infection if not vaccinated. For cluster-randomized trials we accounted for clustering effects by adjusting the effective sample size [19]. We considered outcomes only in those who received the intervention or control vaccine. This is more relevant than the intention to treat population, as the model requires an estimate of vaccine effectiveness in those who actually receive the vaccine and separately accounts for incomplete coverage. In sensitivity analysis 3, the VE for LAIV was estimated using only studies predominantly conducted in Asia without imposing any age restrictions.

When modeling vaccination policies it was assumed that in all cases vaccination started on May 1st and continued for 90 d. This date was chosen to coincide with the start of the new school year in Thailand and because it is shortly after the southern hemisphere influenza vaccine, which is recommended in Thailand, becomes available. April–June is also considered the optimal time for influenza vaccination in most southern and southeastern Asian countries lying north of the equator [20]. The number of children vaccinated per day was assumed to be constant within each age group and was chosen to ensure that target vaccine coverage was reached at 90 d after vaccination started. Amongst children initially susceptible when vaccinated, it was assumed that a proportion given by the vaccine effectiveness was successfully immunized and that vaccine effectiveness remained constant over a 12-mo period. Once vaccinated (with one or two doses depending on age group), children were not vaccinated again in the same year. In the base case analysis, vaccine coverage was 66% in target age groups. Sensitivity analysis 4 assumed 50% coverage.

#### Health outcome model

We estimated DALYs averted by vaccination policies 1 to 7 compared with policy 0. This analysis accounted for life years lost due to deaths resulting from influenza infection, DALYs lost due to nonfatal influenza infection (accounting both for hospitalized pneumonia and influenza cases and for nonhospitalized cases), and adverse events resulting from vaccination.

Estimates of age-specific mortality attributed to each influenza subtype in each of the 4 y were obtained from a separate Bayesian analysis (S4 Table within S1 Text) [21]. Many of those dying from influenza are likely to have co-morbidities that are associated with reduced life expectancy for reasons unrelated to influenza. The health outcome model accounted for this by using priors derived from previous estimates of life years lost as a result of influenza infections in Hong Kong accounting for underlying illness, adjusting for the shorter life expectancy in Thailand [22].

Estimates of DALYs lost due to hospitalized pneumonia and influenza cases were derived from Lugner et al. [23]. Estimates of DALYs lost due to nonhospitalized pneumonia cases and vaccine adverse events were obtained from Prosser et al. [24]. Further details are given in Table 4 and S5 Table within S1 Text.

**Table 4:** Prior distributions for health outcome and cost parameters and their sources.

Category	Parameter	Prior Distribution	Mean (95% CrI)	Source
**Outcome-related parameters**	**DALYs lost per symptomatic, non-medically attended case**	Gamma	0.005 (0.002, 0.009)	[24]
	**DALYs lost per case treated as an outpatient**	Gamma	0.008 (0.0002, 0.03)	[23]
	**DALYs lost per hospitalized case**	Gamma	0.022 (0.0005, 0.09)	[23]
	**DALYs lost per vaccine dose due to adverse events**	Gamma	3 × 10^-8^ (3 × 10^-9^, 1 × 10^-7^)	See S1 Text
	**Life years lost due to death from influenza infection**	Derived distribution^a^		[22]
	0–17 y		76 (62, 90)	
	18–59 y		9 (8, 10)	
	≥60 y		7 (5, 9)	
**Vaccination-related costs**	**Direct medical cost of vaccination**	Constant		
	TIV		11.3	See Methods
	LAIV		18.1	See Methods
	**Logistics cost**	Gamma	1.0 (0.7, 1.5)	[25]
	**Administrative cost**	Gamma		
	2–5 y		6.6 (4.8, 8.7)	[26]
	6–17 y		10.4 (5.8, 21.0)	[26]
	**Direct medical cost due to vaccine adverse events**	Gamma		See S1 Text
	2–5 y		0.029 (0.007, 0.084)	
	6–11 y		0.018 (0.003, 0.062)	
	12–17 y		0.005 (0.001, 0.017)	
	**Direct non-medical costs of vaccination (i.e., transportation and meals) due to administration at hospital/health service center for age group 2–5 y**	Gamma	15.4 (14.0, 16.7)	[26]
	**Direct non-medical costs due to vaccine adverse event**	Gamma		See S1 Text
	2–5 y		0.08 (0.02, 0.18)	
	6–11 y		0.04 (0.00, 0.12)	
	12–17 y		0.02 (0.00, 0.08)	
	**Indirect or time cost due to vaccine administration**	Gamma		[26]
	2–5 y		1.7 (0.9, 2.7)	
	6–11 y		0.4 (0.2, 0.7)	
	12–17 y		0.4 (0.2, 0.7)	
	**Indirect or time cost due to seeking treatment of adverse event**	Gamma		See S1 Text
	2–5 y		0.017 (0.004, 0.045)	
	6–11 y		0.009 (0.001, 0.029)	
	12–17 y		0.006 (0.0005, 0.021)	
**Total cost of influenza**	**Cost per symptomatic, non-medically attended case**	Gamma		See S1 Text and Methods
	<2 y		1.1 (1.0, 1.3)	
	2–5 y		0.8 (0.7, 0.9)	
	6–11 y		1.5 (1.2, 1.9)	
	12–17 y		0.2 (0.2, 0.3)	
	18–59 y		0.2 (0.2, 0.3)	
	≥60 y		0.2 (0.2, 0.3)	
	**Cost per symptomatic case treated as an outpatient**	Derived distribution^a^		
	<2 y		167 (42, 437)	
	2–5 y		166 (41, 447)	
	6–11 y		165 (40, 432)	
	12–17 y		167 (41, 429)	
	18–59 y		125 (49, 260)	
	≥60 y		124 (48, 251)	
	**Cost per symptomatic case treated as an inpatient**	Derived distribution^a^		[27]
	<2 y		752 (627, 902)	
	2–5 y		730 (687, 776)	
	6–11 y		694 (583, 823)	
	12–17 y		565 (546, 584)	
	18–59 y		1,043 (992, 1,098)	
	≥60 y		834 (723, 962)	

All cost parameters are given as international dollars per case.

^a^See S1 Text.

CrI, credible interval.

#### Cost model

We adopted the societal viewpoint, taking into consideration direct medical, direct non-medical, and indirect costs incurred from each policy option. The unit cost of outpatient care for influenza illness was derived from a cross-sectional study conducted in Thailand [27]. Unit costs of hospitalization and vaccine-related adverse events were derived from the National Hospital Database, containing information on inpatient care from all public and private hospitals throughout the country. The cost of medically attended Guillain-Barré syndrome was considered both in influenza infection and vaccination groups [28]. We assumed that individuals with symptomatic influenza who do not seek medical attention require only over-the-counter medication and do not need to absent themselves from school or the workplace (as employers and schools in Thailand typically require a medical certificate in the case of absence from work or school). Direct medical costs of vaccination were the summation of vaccine acquisition cost, supply chain and logistic costs, administration costs, and vaccine-related adverse event costs. The TIV acquisition cost of 201 baht (11.3 international dollars [I$]) per dose was derived from the Thai Government Pharmaceutical Organization’s average purchasing price between 2009 to 2012 (personal communication from Dr. Sit Thirapakpoomanunt, Government Pharmaceutical Organization, Thailand). Because LAIV is not currently available in Thailand, in the base case analysis we assumed the same LAIV/TIV price ratio as reported in the US Centers for Disease Control and Prevention’s vaccine price list [29]. This corresponded to an assumption that LAIV is 1.6 times more expensive than TIV, at 322 baht (I$18.1) per dose, and is broadly in agreement with studies in the US, Canada, and Germany [30–32].

Children aged 6–17 y were assumed to incur an additional administration cost for the school-based vaccination program. Only preschool children aged 2–5 y incurred the direct non-medical cost due to receiving the vaccine at a hospital or health service center, which includes expenses for food and transportation. All costs were adjusted to 2012 Thai baht using the Consumer Price Index [33]. For intercountry comparisons, costs were converted into international dollars using the purchasing power parity exchange rate of I$1 = 17.76 baht (year 2012 values) [34]. Prior distributions for cost parameters are summarized in Table 4. Full cost vectors used in the analysis are given in S6 Table within S1 Text.

#### Derivation of model priors

Informative prior distributions were obtained from published literature and formal expert opinion elicitations (Tables 3 and 4). Elicited priors were used for parameters that either had not been reliably estimated or had been estimated only in locations where their values were thought likely to differ substantially from those in Thailand. The elicitation procedure followed the Sheffield Elicitation Framework (SHELF, version 2.0) [35]. For our base case assumption for the proportion of influenza infections that are symptomatic, we based our prior on a review of human volunteer challenge studies that suggested that about two-thirds of infections are symptomatic [15]. In sensitivity analysis 8, we used a distribution that supported much lower values (between about 14% and 19%) chosen to be consistent with a cohort study in Viet Nam [36].

#### Model fitting

We estimated model parameters for influenza A/H1N1, A/H3N2, and B, obtaining independent estimates for each of the four seasons, assuming that infection with one virus subtype did not alter the risk of infection with another. Sampled outcomes from the four 1-y runs were combined to obtain final estimates of health benefits and costs associated with the vaccination policies.

Bayesian inference was performed using a Markov chain Monte Carlo approach using WinBUGS version 1.4, which was also used to calculate outcome measures of interest, accounting for parameter uncertainty [37]. The dynamic transmission model was implemented in Component Pascal as a hard-wired plug-in to WinBUGS using the BlackBox Component Builder (Oberon Microsystems). Full implementation details are presented in S1 Text, and model code is available at http://goo.gl/htrvrk.

## Results

### Vaccine Efficacy in School-Aged Children

For both TIV and LAIV, there were two trials evaluating VE in children within the target age range [38–41]. Meta-analysis gave VE estimates for TIV and LAIV of 48.5% (95% credible interval [CrI] 0.7%, 68.9%) and 90.0% (95% CrI 84.9%, 94.2%). When we considered only trials predominantly conducted in Asia, the estimated LAIV VE was 67.1% (95% CrI 59.2%, 73.7%) (S2 and S3 Tables within S1 Text) [42,43].

### Transmission and Epidemiological Models

**Fig 2:**
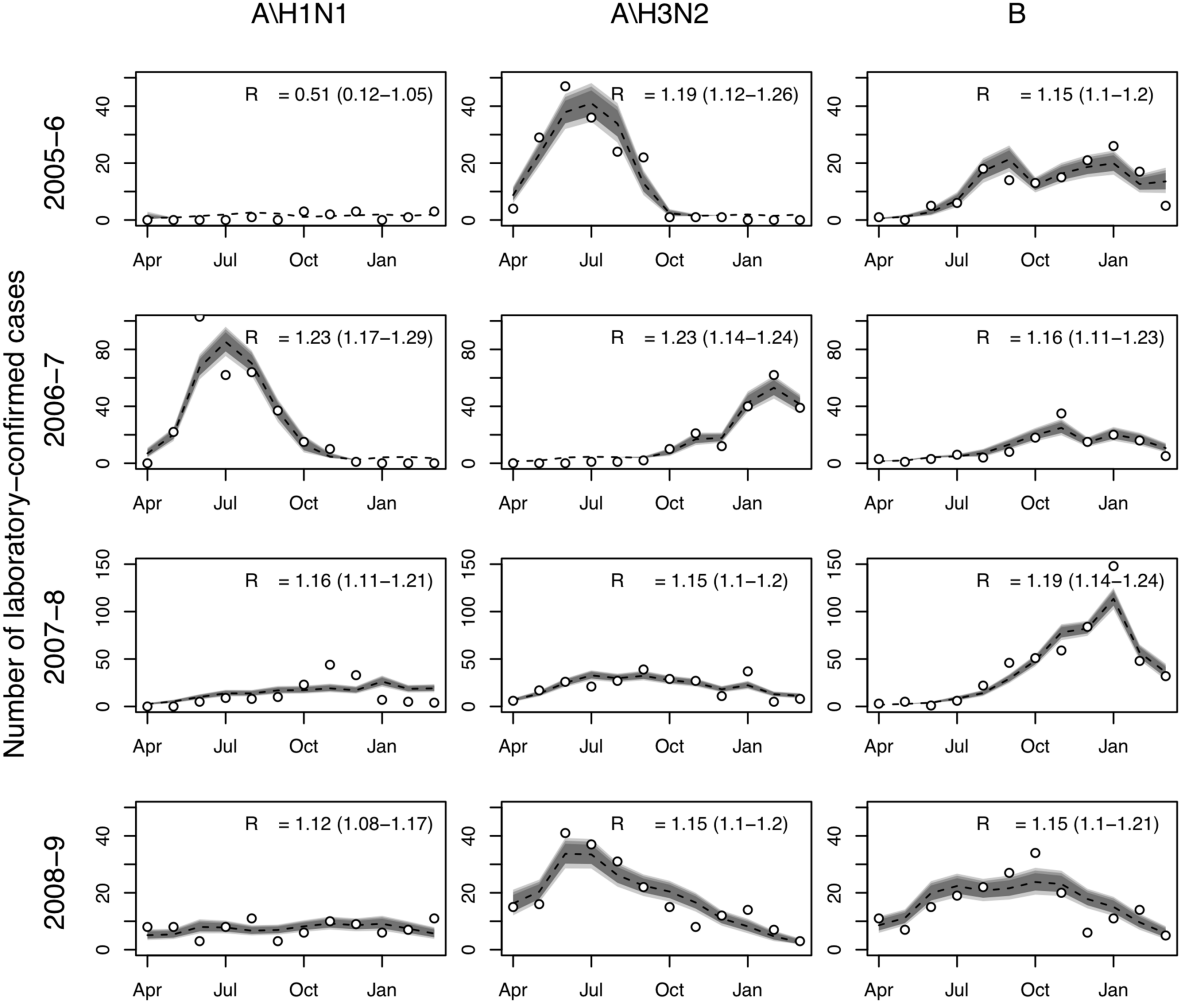
Model fits to laboratory-confirmed influenza surveillance data. Monthly numbers of laboratory-confirmed influenza cases (circles) and model predictions: median (broken line) and 95%, 90%, and 80% (gray shading) prediction intervals for the expected number of cases. Also shown is the estimated value of *R*_0_ and associated 95% CrI.

The transmission model gave good fits to observed influenza surveillance data for most years and subtypes, and under base case assumptions was consistent with subtype-specific pre-epidemic reproduction numbers between 1.1 and 1.3 (Fig 2). Note that temporal variation in the number of laboratory-confirmed influenza cases reflects both changes in positivity rates and changes in the number of isolates tested. For other epidemiological parameters, including immunity at the start of the influenza season, posterior distributions were similar to the priors (S7 Table within S1 Text). The predicted mean serological attack rate (95% CrI) with any of the three influenza subtypes in each of the four modeled years (April 2005–March 2009) was 17% (11%, 22%), 26% (22%, 31%), 19% (16%, 23%), and 13% (9%, 16%), respectively.

### Health Outcome and Cost Models

**Fig 3:**
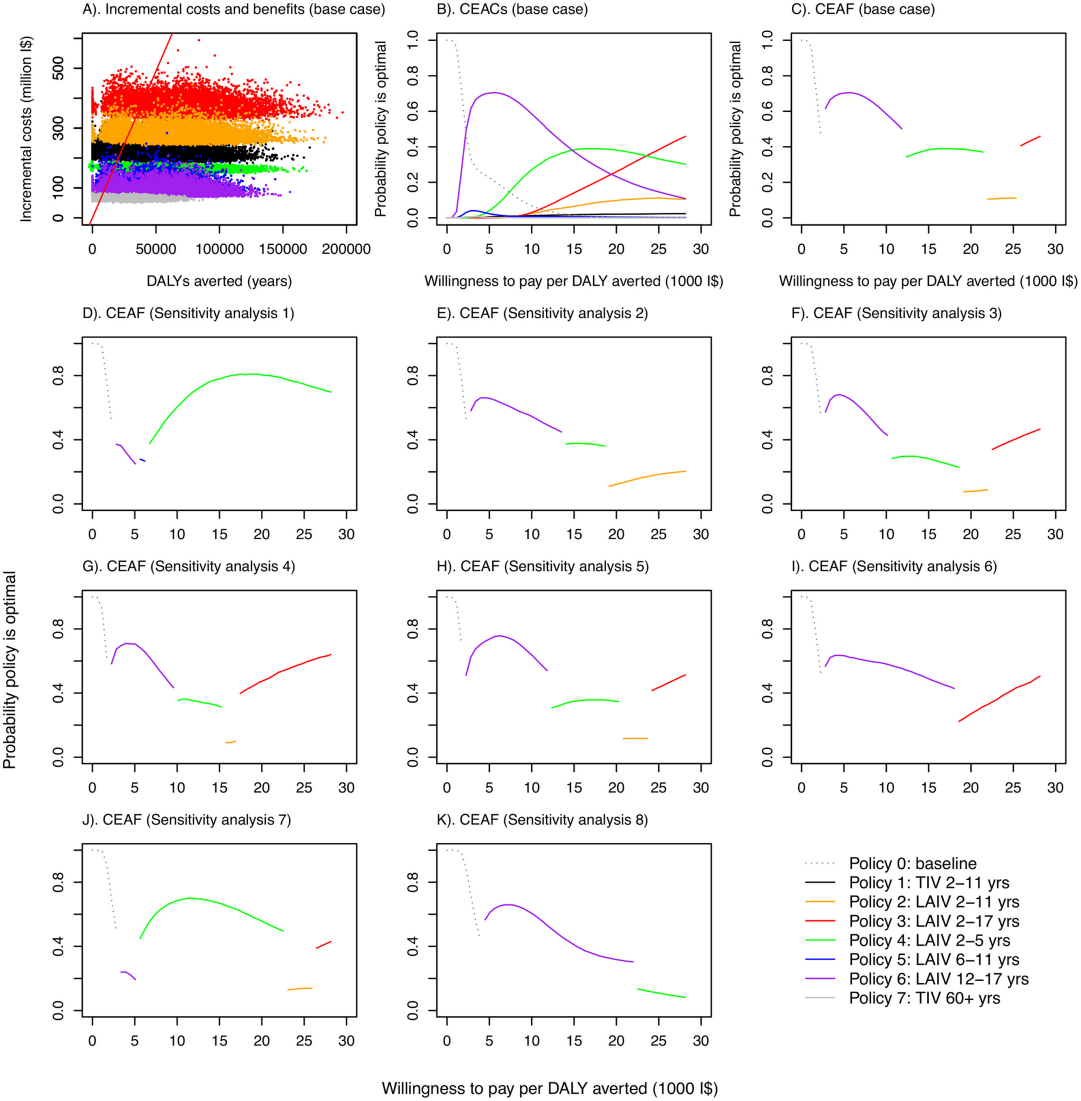
Cost-effectiveness plane, cost-effectiveness acceptability curves, and cost-effectiveness acceptability frontiers. (A) Cost-effectiveness plane showing samples from the posterior distributions of DALYs averted and incremental costs for the seven vaccination policies compared with no vaccination under base case assumptions. Points to the right of the solid red line (which corresponds to a threshold of I$10,000 per DALY averted) would be considered highly cost-effective compared with no vaccination in Thailand according to the WHO threshold [44]. (B) CEACs under base case assumptions. (C–K) CEAFs under base case assumptions and for eight sensitivity analyses.

All vaccination policies considered led to reductions in influenza cases and mortality compared to policy 0 (no childhood vaccination) (Table 5). The largest reductions were seen with policy 3 (vaccinating children aged 2–17 y with LAIV), and the smallest with policy 7 (increasing TIV coverage in those aged ≥60 y to 66%). None of the policies were cost-saving. The largest incremental costs were seen with policy 3, and the lowest with policy 7. Summarizing these results on the cost-effectiveness plane shows that all seven policies have a very high probability of being highly cost-effective at the WHO-recommended threshold (cost per DALY averted less than gross domestic product per capita [44]) compared with policy 0 (Fig 3A). There was, however, large uncertainty in the health benefits of vaccination. In part, this was explained by large annual variation in influenza dynamics.

**Table 5:** Model outcomes showing mean (95% CrI).

Policy	Total Cost (Millions of International Dollars)	Outcomes (Thousands of Cases)				Comparison with No Childhood Vaccination		
		Symptomatic Infections	Outpatient Visits	Inpatient Visits	Deaths			
						Incremental Cost (Millions of International Dollars)	DALYs Averted (Thousands)	ICER
Policy 0: no childhood vaccination	20 (16, 24)	8,560 (4,420, 14,383)	5.3 (0.0, 7.3)	3.3 (1.2, 8.0)	4.3(0.6, 9.2)			
Policy 1: vaccinate those aged 2–11 y with TIV	237 (214, 286)	3,572 (581, 10,651)	2.8(0.0, 7.3)	1.0 (0.4, 2.5)	1.9 (0.2, 6.0)	218 (193, 267)	49 (0, 107)	4,445
Policy 2: vaccinate those aged 2–11 y with LAIV	296 (273, 345)	2,375 (413, 5,200)	2.0 (0.0, 4.8)	0.6 (0.3, 0.8)	1.3 (0.1, 3.2)	276 (252, 325)	61 (12, 121)	4,529
Policy 3: vaccinate those aged 2–17 y with LAIV	392 (359, 451)	2,016 (330, 4,752)	1.8 (0.0, 4.5)	0.5 (0.3, 0.8)	1.1 (0.1, 2.9)	372 (339, 431)	65 (14, 128)	5,748
Policy 4: vaccinate those aged 2–5 y with LAIV	185 (175, 196)	2,863 (590, 5,759)	2.4 (0.0, 5.1)	0.7 (0.4, 1.0)	1.6 (0.2, 3.6)	165 (154, 177)	56 (10, 112)	2,961
Policy 5: vaccinate those aged 6–11 y with LAIV	126 (108, 175)	4,049 (1,648, 7,172)	3.0 (0.0, 5.6)	1.2 (0.6, 2.2)	2.1 (0.3, 4.6)	107 (86, 155)	45 (7, 91)	2,364
Policy 6: vaccinate those aged 12–17 y with LAIV	111 (95, 152)	3,542 (1,085, 6,562)	2.8 (0.0, 5.5)	1.0 (0.5, 1.5)	1.9 (0.2, 4.2)	91 (73, 133)	50 (8, 99)	1,841
Policy 7: increase vaccination coverage in those aged ≥60 y with TIV	85 (77, 94)	6,628 (3,272, 12,132)	4.3 (0.0, 7.3)	2.4 (1.0, 6.7)	2.9 (0.5, 6.8)	65 (57, 74)	23 (0, 54)	2,889

In polices 0–6, it was assumed that 10% of those aged 60 y and over were vaccinated with TIV.

ICER, incremental cost-effectiveness ratio.

Under base case assumptions, policies using LAIV are likely to have the highest INB provided willingness to pay to avert one DALY is not very low. The specific LAIV policy giving the greatest net benefit, however, depends on the precise cost-effectiveness threshold (Fig 3B). Increasing the cost-effectiveness threshold leads to increasing chances that progressively more costly LAIV policies have the highest INB. These more costly policies are those that include coverage in children 5 y and under, with costs increasing in line with added coverage for children in older age groups. Under base case assumptions, policy 6 (vaccinating those aged 12–17 y with LAIV, the least costly LAIV policy) was the most likely to have the highest INB for cost-effectiveness thresholds between about I$3,000 and I$15,000 per DALY averted, while policy 4 (LAIV for those aged 2–5 y) and policy 3 (LAIV for those aged 2–17 y) were the most likely to have the highest INB at higher thresholds. The CEAF shows that policy 3 becomes optimal only at a threshold above I$25,000 per DALY averted (Fig 3C). Under the base case assumptions, any of policies 1–7 is always preferred to policy 0 if the threshold is above I$2,000 per DALY averted.

Sensitivity analyses show that the finding that LAIV policies are cost-effective is robust to changes in assumptions about contact patterns (Fig 3D), assumptions about immunity (Fig 3E, 3I and 3J), studies used to estimate VE (Fig 3F), vaccine coverage in target age groups (Fig 3G), prior assumptions about reproduction numbers (Fig 3H), and the proportion of infections that are symptomatic (Fig 3K). In all of these sensitivity analyses, all increased vaccination policies were found to be likely to be cost-effective compared to no increased vaccination at a threshold value of I$10,000 per DALY averted, and LAIV policies were preferred to the TIV policy. The optimal age groups to vaccinate for a given threshold ceiling, however, were sensitive to the assumptions modified in the sensitivity analyses. Alternative assumptions also led to different estimates of epidemiological parameters. When baseline immunity was assumed to be considerably higher than that derived from the expert elicitation exercise (sensitivity analyses 2, 6, and 7), estimated reproduction numbers substantially increased, with estimates ranging from 1.3 to 2.2 for influenza A (except for A/H1N1 in 2005–2006), and from 1.3 to 1.6 for influenza B (S11, S17 and S19 Tables within S1 Text).

**Fig 4:**
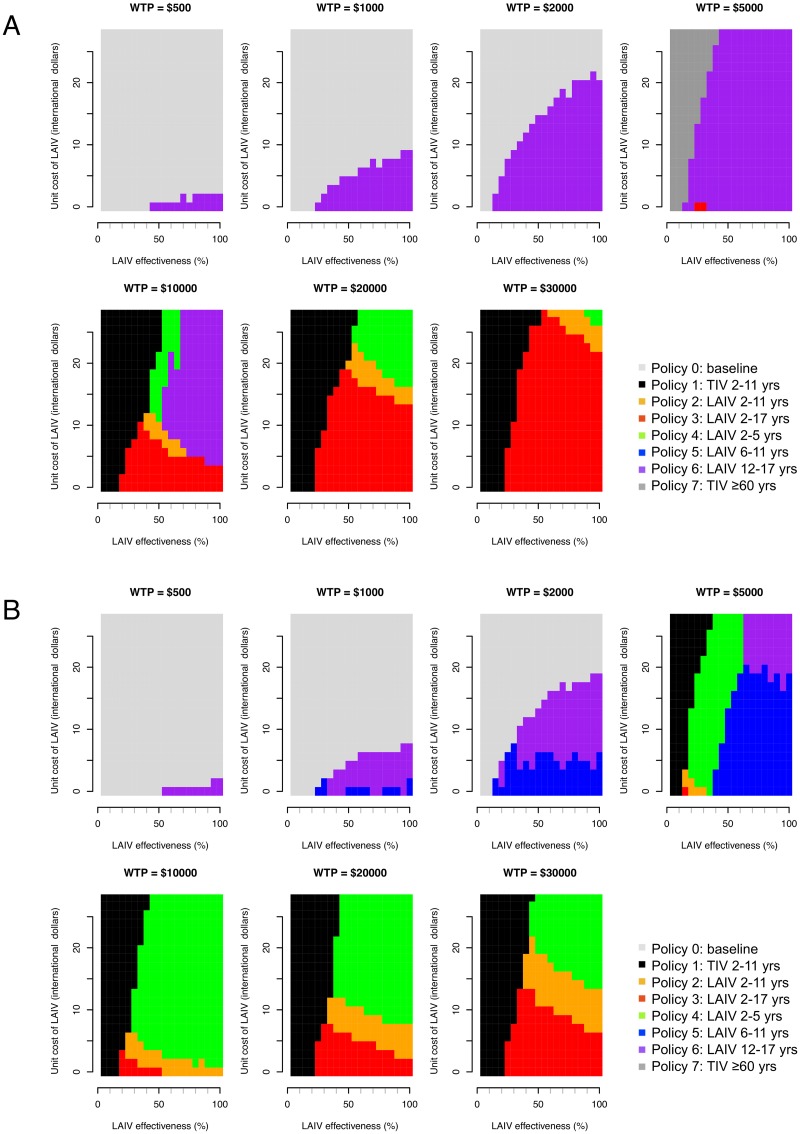
Threshold analysis. Figure shows how the optimal policy (defined as the policy that maximizes the INB) changes with LAIV effectiveness, unit cost of LAIV, and willingness to pay (WTP) per DALY averted (cost-effectiveness threshold). (A) Base case mixing matrix. (B) Contact matrix based on physical contacts only.

Effectiveness of LAIV with large-scale deployment may differ substantially from efficacy estimates from trials [45]. There is also large uncertainty concerning likely LAIV costs in Thailand. For these reasons we performed a threshold analysis to determine the optimal policy over a grid of values for LAIV effectiveness, LAIV unit cost, and cost-effectiveness threshold values (Fig 4). This analysis shows that for a threshold of I$1,000 per DALY averted, vaccination is cost-effective only if the unit cost of LAIV is below about I$10. In this case, under base case assumptions, vaccination of those aged 12–17 y (policy 6, the least costly LAIV policy) would be preferred to no vaccination if LAIV effectiveness is sufficiently high. If the threshold is I$5,000 or more, increased vaccination with TIV (policies 1 and 7) can become optimal if LAIV effectiveness is sufficiently low. For higher LAIV effectiveness values, policies 2, 3, 4, and 6 can all be optimal depending on vaccine effectiveness and cost values. Generally, the range of values for LAIV cost and effectiveness at which high-cost (and high-coverage) LAIV policies are optimal increases in line with the cost-effectiveness threshold. Interestingly, however, for threshold values of I$10,000 or more, at a given LAIV unit cost, as LAIV effectiveness increases, less costly LAIV vaccination policies are sometimes optimal. Sensitivity analysis using an alternative contact matrix (derived from only physical contacts) gives the same general picture, and LAIV and TIV policies are optimal over a similar range of parameter values, though in many cases the optimal age groups to vaccinate with LAIV differ (Fig 4B).

In the base case analysis, the EVPI reaches a maximum of about I$60 million at a cost-effectiveness threshold value of about I$25,000 per DALY averted (S7 Fig within S1 Text). This finding reflects the large uncertainty as to whether policy 2, 3, or 4 is optimal at this threshold value. At higher threshold values the EVPI declines, reflecting increased certainty that policy 3 is optimal. The EVPPI analysis shows that under base case assumptions and with a cost-effectiveness threshold value of about I$10,000 per DALY averted, perfect information about most parameters would not lead to improved decisions (S8 Table within S1 Text).

**Fig 5:**
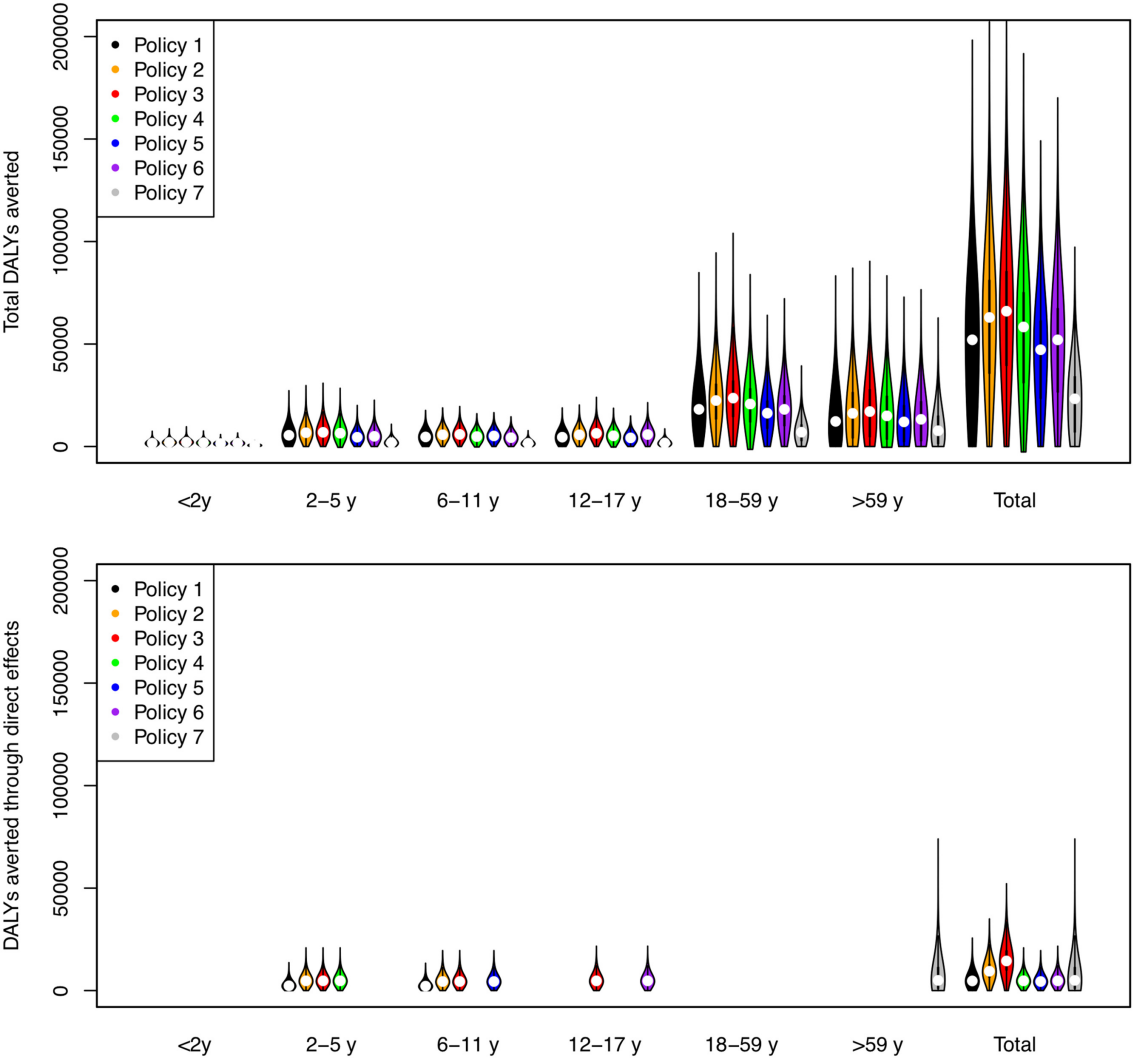
DALYs averted by vaccination policies in total and as a result of direct vaccine effects. The width of bars corresponds to the probability density, the central black line within each bar represents the interquartile range of the DALYs averted, and the white circle represents the median value. Note that all DALYs averted in those 18 y and over or under 2 y are by definition indirect in policies 1–6.

**Fig 6:**
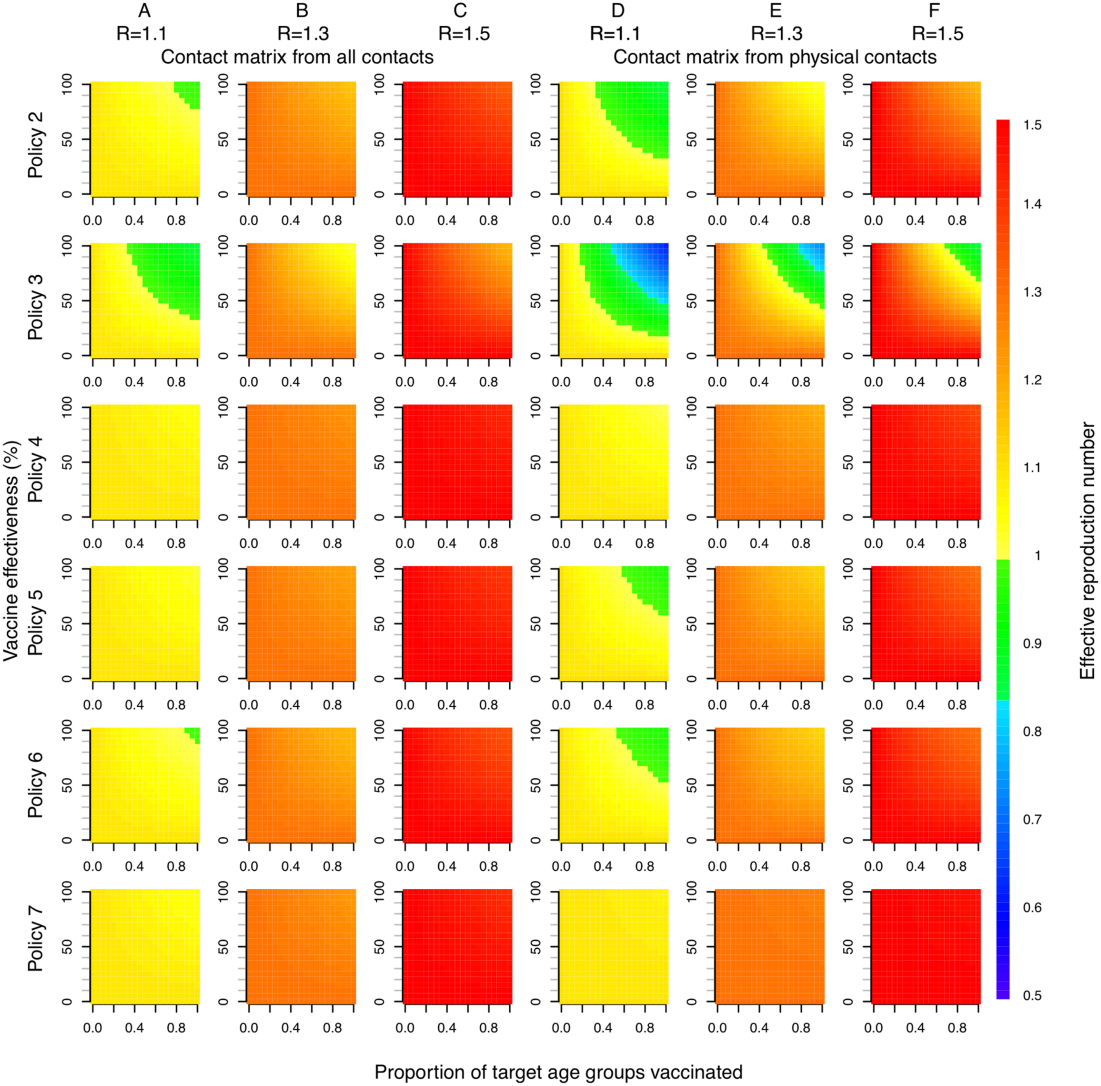
Effective reproduction number as a function of vaccination policy, coverage, and vaccine effectiveness. Color indicates effective reproduction number after vaccination. Columns A, B, and C use a contact matrix derived from all contacts recorded. Columns D, E, and F use a contact matrix derived from physical contacts only. In both cases, baseline immunity assumptions at the start of each influenza season are taken as the mean estimated for each of the six age groups when fitting to data under base case assumptions (21%, 25%, 33%, 33%, 38%, and 37%). *R* values in column headings indicate the assumed effective reproduction number prior to vaccination.

Direct benefits from vaccination represent only a small proportion of the total health benefits of vaccination, even when coverage extends to all children aged 2–17 y (Fig 5). Most of the health benefit comes from DALYs averted in older age groups. To gain further insight into these indirect effects, we consider how the effective reproduction number, *R* (the expected number of secondary infections caused by a typical infective individual), changes with different vaccination scenarios and model assumptions (Fig 6). This analysis shows that under base case mixing assumptions (Fig 6, columns A–C), vaccination alone is unlikely to reduce *R* to below one (achieving herd immunity) unless transmissibility is particularly low and both coverage and vaccine effectiveness are high. At intermediate levels of transmissibility (*R* = 1.3) under policy 3, vaccination can come close to reducing *R* to below one (the herd-immunity threshold below which major epidemics will not occur). While this would not prevent influenza epidemics, it would greatly curtail them, giving large indirect benefits. In contrast, if contact patterns between age groups are derived from physical contacts (Fig 6, columns D–F), we estimate much larger effects of vaccinating children and a much higher chance of achieving herd immunity. Under both mixing assumptions, vaccinating only those aged 2–5 y (policy 4) or only the elderly (policy 7) does not appreciably change *R*.

## Discussion

We found that, under plausible assumptions, an influenza vaccination program for children can represent good value for money in Thailand. All childhood vaccination policies considered would meet WHO criteria for being classed as highly cost-effective compared to no vaccination, and the LAIV was consistently preferred. This conclusion held even if LAIV effectiveness was assumed to be much lower than estimated from clinical trials. Most of the health benefits resulted from reduced mortality in older age groups, a finding in accordance with a previous evaluation of the impact of childhood influenza vaccination in England and Wales [2]. This finding contrasts with recent economic evaluations of other possible large-scale pediatric vaccination efforts, which found that neither pneumococcal conjugate vaccine nor rotavirus vaccine are likely to be cost-effective in Thailand [46,47].

The optimal age range of children to vaccinate was, however, sensitive both to assumptions about which there is considerable uncertainty and to the cost-effectiveness threshold. Under our base case assumptions, the policy of immunizing those aged 2–17 y with LAIV became cost-effective at a threshold value of approximately I$26,000 per DALY averted. This was also the only policy able to consistently attain herd immunity at levels of vaccine effectiveness estimated from clinical trials. The optimal LAIV policy was particularly sensitive to the data used to derive contact matrices describing interactions between age groups. In humid countries, such as Thailand, there are some reasons for thinking that droplet and contact transmission of influenza may be more important than aerosol transmission [48], suggesting that physical contacts (as used in sensitivity analysis 1) might be the most relevant. For now, however, this remains unclear.

Estimates of reproduction numbers for different influenza subtypes at the start of each season and under base case assumptions were mostly between 1.1 and 1.3, lower than typically reported in temperate countries [2]. The model gave generally good fits to surveillance data, and the model estimates of annual serological attack rates (17%, 26%, 19%, and 13% for the four influenza seasons of April 2005–March 2009) are consistent with estimates obtained from a recent sero-epidemiological study in Viet Nam which estimated that, over three successive influenza seasons (2007–2010), 21%, 26%, and 17% of people aged over 5 y acquired at least one influenza infection [36]. These findings provide reassurance that a simple age-structured transmission model is able to capture essential features of influenza dynamics in tropical Southeast Asia.

We are not aware of other studies evaluating the cost-effectiveness of childhood influenza vaccination in low- or middle-income countries. While there have been cost-effectiveness studies of childhood seasonal influenza vaccination in high-income countries, most have used only static models, neglecting the important indirect effects [1,30,49]. An important exception is a recent study by Pitman et al. which suggested that offering LAIV to all children aged 2–18 y is a very cost-effective policy in England and Wales, with an incremental cost-effectiveness ratio of less than £300 per quality-adjusted life year, a finding broadly in agreement with our own [50]. Our analysis, however, suggests that similar policies have the potential to have a particularly large impact on influenza epidemiology in Thailand and, potentially, other tropical developing countries. This is a consequence of the much higher proportion of the population represented by children in typical developing countries and the apparently lower levels of transmission in predominantly rural tropical populations. These two factors mean that by vaccinating children it might be possible to approach coverage levels necessary to prevent sustained person-to-person transmission of influenza. However, whether such a herd-immunity threshold can be achieved with mass influenza vaccination in practice is unclear. First, vaccine effectiveness may be lower in tropical Southeast Asia than in temperate countries, possibly as a result of greater antigenic diversity [51]. Second, a large-scale vaccination campaign has the potential to alter the competitive landscape between influenza subtypes, potentially resulting in selection for subtypes against which the vaccine has poor effectiveness. The chances of this happening depend on the circulating strains, the degree of cross-protection to these offered by the vaccine, the degree of prior immunity to circulating strains, and the importance of strain-transcending immunity in influenza dynamics [52]. These considerations suggest that vaccine effectiveness against influenza when used as part of a mass immunization campaign might be much lower than observed efficacy in randomized trials. At present we do not have a good enough theoretical understanding of the forces shaping influenza strain dynamics to make robust predictions of how important such considerations are [53].

The assumption that some proportion of those vaccinated are fully protected against infection and the rest fully unprotected is also a simplification, and variation between individuals in protection due to vaccination is an important determinant of the population-level impact of vaccination [54]. Vaccines may also have several components of effectiveness (reducing susceptibility, transmissibility, and progression to symptomatic infection) [3]. In the absence of reliable data to inform a model accounting for these complexities, we performed a threshold analysis where LAIV effectiveness ranged from 0% to 100%. This analysis showed that even with much lower vaccine effectiveness than assumed in the base case, childhood vaccination with LAIV would be likely to represent good value for money.

The new quadrivalent seasonal influenza vaccine with protection against two lineages of influenza B would be likely to provide somewhat greater health benefits than TIV in elderly people. Two modeling studies indicated that this vaccine has the potential to be cost-effective compared to TIV in high-income settings [55,56]. Currently, however, reliable efficacy data to inform an analysis in a middle-income setting are lacking, though in Thailand between 2004 and 2008, influenza B viruses of both Victoria and Yamagata lineages co-circulated, and about half of the circulating influenza B viruses may have been mismatched with the influenza B component of the annual vaccine [16]. This suggests that benefits from using the quadrivalent vaccine are likely. However, unless the difficulties in achieving even moderate vaccine coverage levels in the elderly in Thailand can be overcome, the impact of using a slightly more efficacious vaccine in the elderly is likely to be small.

Strengths of our study include the detailed accounting for health and economic impacts, the integrated, dynamic age-structured model, the coherent propagation of different sources of uncertainty through the model, and extensive sensitivity analyses. There are also a number of limitations. First, we did not explicitly account for waning immunity and antigenic drift: both are implicitly accounted for in the proportion initially immune. Similarly, we assumed that vaccination in one year confers protection against influenza strains circulating only that year, not subsequent years (though since this is a conservative assumption, it should not affect our conclusion that LAIV is likely to be cost-effective). We also neglected explicit consideration of births and deaths unrelated to influenza because we ran the model only over short time periods. More importantly, we lacked data concerning prior immunity to influenza in different age groups, and in our base case analysis, we relied on expert judgment. However, we performed additional sensitivity analyses that show that our conclusions are robust to alternative immunity assumptions. Another limitation is the assumption that influenza A/H1N1, A/H3N2, and B epidemics proceed independently. This is unlikely to be strictly true, but modeling such interactions would require either a detailed mechanistic understanding of the interactions between influenza subtypes or longer time series data to allow us to make inferences about such interactions. Both are currently lacking. Other potential refinements to the modeling approach include accounting for spatial and seasonal effects. Again, both would require much richer datasets than those currently available.

A further consequence of our imperfect understanding of the genetic and immunological forces shaping influenza evolution is that we cannot make reliable predictions about the long-term effects of vaccination. If prior immunity to circulating influenza strains in a given season is low (as our elicitation exercise suggested and previous modeling studies have assumed), then even an immunization program that achieves herd immunity would not be expected to have a large effect on the immunological profile in the unvaccinated population. Conversely, if there is significant cross-protection between even distantly related influenza strains, as other models of the evolutionary dynamics of influenza have posited [51], then immunization programs could result in large long-term reductions in immunity levels in unvaccinated age groups, which might have long-term consequences. Thus, apparently quite theoretical and unresolved questions about the forces shaping influenza strain dynamics could have large practical consequences for vaccination programs. Encouragingly, we note that in Japan, where most school children were vaccinated against influenza between 1962 and 1987, the policy was associated with a large sustained reduction in mortality attributed to influenza and has been estimated to have prevented between 30 and 50 deaths per 100,000 annually [8].

It is also unknown whether less frequent influenza infections in the elderly will lead to increased disease severity from influenza infections or other respiratory pathogens (such interactions between different respiratory pathogens have been reported in children [57]). If this is the case, our analysis (and all others we are aware of) may overestimate the reduction in burden of disease associated with influenza vaccination.

In summary, our findings suggest that seasonal influenza vaccination of children with LAIV is likely to represent good value for money in the Thai setting and potentially in many other developing economies. Fundamental uncertainties about influenza remain, however, and for this reason we believe proposals for large-scale community-based controlled trials of policies to vaccinate children against influenza are as relevant to low- and middle-income settings as they are to high-income countries [3]. The results of the present study were used to inform the development of the Health Promotion and Disease Prevention Benefit Package under the universal health coverage in Thailand. It was proposed that a school-based seasonal influenza vaccination program should be piloted in a few selected provinces in fiscal year 2016 before considering scale-up to a nationwide program. The design and evaluation of such pilot studies will need careful consideration, but prospective community-based household cohort studies in vaccinated and unvaccinated populations may be particularly valuable as part of this work [36].

## Supporting Information

S1 Text Supplementary data, methods, and results. (PDF)

## Abbreviations

CEAC: cost-effectiveness acceptability curve
CEAF: cost-effectiveness acceptability frontier
CrI: credible interval
DALY: disability-adjusted life year
EVPI: expected value of perfect information
EVPPI: expected value of partial perfect information
ILI: influenza-like illness
INB: incremental net benefit
I$: international dollars
IP: influenza pneumonia
LAIV: trivalent live-attenuated influenza vaccine
TIV: trivalent inactivated influenza vaccine
VE: vaccine efficacy
