# Supplementary material for: Seasonal Influenza Vaccination for Children in Thailand: A Cost-Effectiveness Analysis
Source: PLoS Med. 2015 May 26;12(5):e1001829. doi: 10.1371/journal.pmed.1001829 (PMC4444096; doi:10.1371/journal.pmed.1001829)
Supplement: S1 Text — (PDF) [file pmed.1001829.s001.pdf]

Supporting Information

This file contains five sections:

Section 1: Supplementary Data presents additional influenza data used in the analysis, not presented in the main text.
Section 2: Supplementary Methods, presents detailed methods describing the model assumptions and analysis.
Section 3: Supplementary Results, presents additional model results.
Section 4: References for Supplementary Information.
Section 5: Model code.

1. Supplementary Data

S1 Figure. Monthly influenza surveillance data by age group and the proportion of tests positive for the three different influenza types from Thailand, April 2005-Mar 2009. Data shown are laboratory-conformed influenza cases in hospital in-patients (IP), influenza like illness (ILI) in outpatients and the proportion of isolates tested that were PCR positive for A/H1N1, A/H3N2 and influenza B. IP and ILI are shown for the six age groups used in the analysis: less than two years (dark blue); 2-5 years (green); 6-11 years (yellow); 12-17 years (orange); 18-59 years (red); and ages 60 years and up (purple).

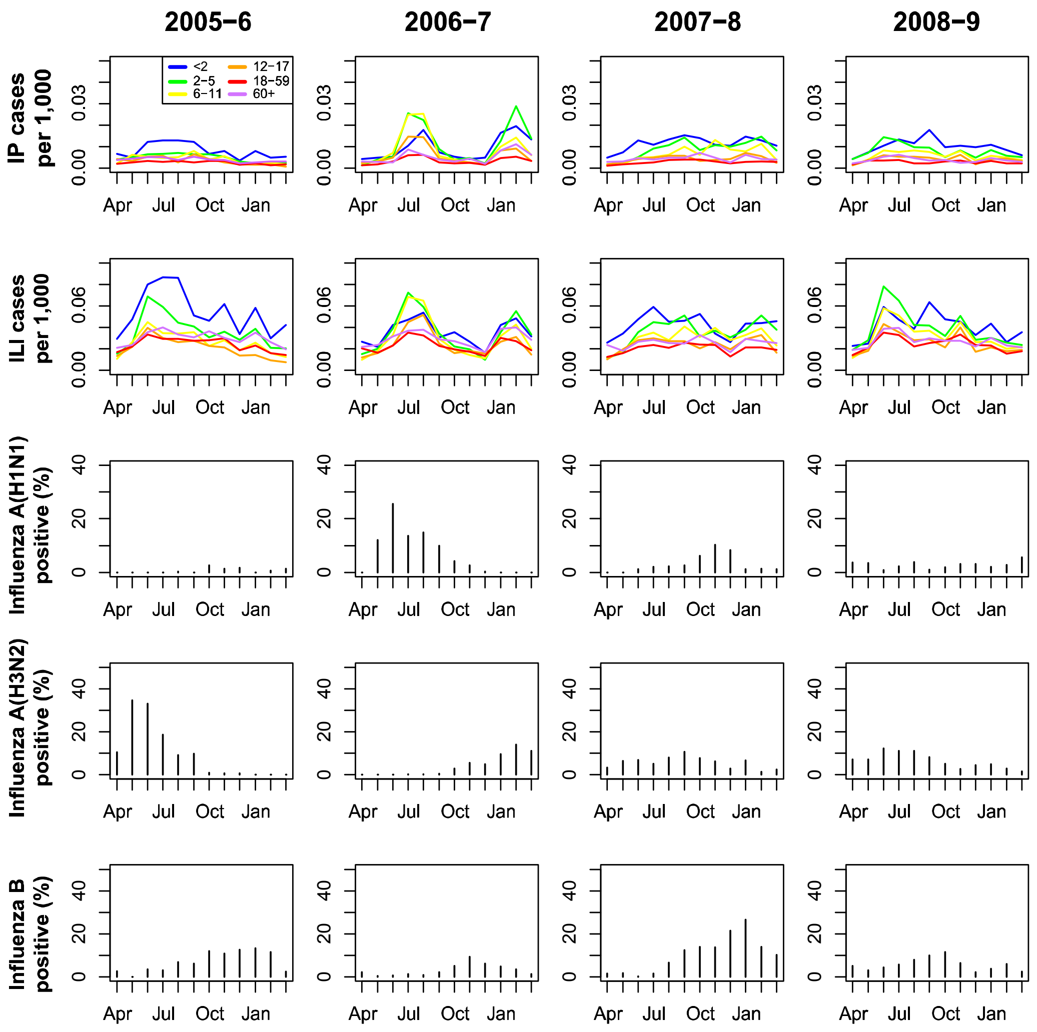

2. Supplementary Methods

2.1 Transmission and epidemiological model

The underlying transmission model consists a set of coupled ordinary differential equations

$$
\begin{aligned}
dS_i/dt &= -\lambda S_i \sum_j \beta_{ji} I_j/N_i - v_i(t) S_i/(N_i - V_i) \\
dS_i^v/dt &= -\lambda S_i^v \sum_j \beta_{ji} I_j/N_i + v_i(t)(1-f) S_i/(N_i - V_i) \\
dE_i/dt &= \lambda (S_i + S_i^v) \sum_j \beta_{ji} I_j/N_i - \theta E_i \\
dI_i/dt &= \theta E_i - \gamma I_i \\
dR_i/dt &= \gamma I_i + v_i(t) f S_i/(N_i - V_i) \\
dV_i/dt &= v_i(t)
\end{aligned}
$$

The model variables are:

S_i the number susceptible in age group *i* who have not been vaccinated;
$S^v{}_i$ the number susceptible in age group *i* who have been vaccinated but failed to develop immunity;
E_i the number latently infected (but not yet infectious) in age group *i* ;
I_i the number infectious in age group *i* ;
R_i the number immune in age group *i* ;
V_i the number in age group *i* who have been vaccinated (whether or not they develop immunity).

The model parameters are:

f vaccine effectiveness;
λ the transmission scaling parameter;
β_{ji} elements of the contact-matrix which are proportional to rate at which a given individual in age group *i* makes potentially infectious contact with members of age group *j*;
θ rate of progression from latently infected to infectious;
γ rate of recovery from infectious state;
$v_i(t)$ the time-varying vaccination rate in age group *i*.

2.2 Demographic data and contact data.

There were six age groups and population size estimates for these were obtained from the National Statistical Office of Thailand (S1 Table). Contact patterns between these age groups were derived from a diary-based contact survey in Thailand carried out by the The International Health Policy Program, Thailand (manuscript in preparation). In the base case analysis we used all contacts. In sensitivity analysis 1 we derived the matrix using data on only physical contacts.

S1 Table: Age groups and population sizes.

Age group	Population size (N)
< 2 y	1,637,070
2-5 y	4,000,962
6-11 y	6,131,617
12-17 y	6,227,226
18-59 y	38,377,518
≥60 y	5,792,970

2.3 Vaccination Model

We considered both fixed and random effect models to estimate vaccine efficacy. Because the random effects model did not show any improvement in model fit (based on the deviance information criterion), we used results from the fixed effects model in all subsequent analysis:

$c_{v,i} \sim \text{binomial}(p_{v,i}, n_{v,i})$
$c_{c,i} \sim \text{binomial}(p_{c,i}, n_{c,i})$
$\text{logit}(p_{c,i}) = m_i$
$\text{logit}(p_{v,i}) = m_i + \delta$
$\delta \sim \text{normal}(\mu_1, \sigma_1^2)$
$m_i \sim \text{normal}(\mu_2, \sigma_2^2)$
$\mu_1 \sim \text{normal}(0, 10^5)$
$\mu_2 \sim \text{normal}(0, 10^5)$
$1/\sigma_1^2 \sim \text{gamma}(0.001, 0.001)$
$1/\sigma_2^2 \sim \text{gamma}(0.001, 0.001)$
$\mu_{new} \sim \text{normal}(\mu_2, \sigma_2^2)$
$VE = (1-\exp(\delta))/(1+\exp(\mu_2 + \delta))$
$VE_{predict} = (1-\exp(\delta))/(1+\exp(\mu_{new} + \delta))$

Where:
the two parameters for the gamma distribution specify the shape and rate, respectively;
$c_{v,i}$ is the number of cases in the vaccine group in study *i*;
$c_{c,i}$ is the number of cases in the control group in study *i*;
$n_{v,i}$ is the number of patients in the vaccine group in study *i*;
$n_{c,i}$ is the number of patients in the control group in study *i*;
δ is the change in the log odds of influenza infection associated with the vaccine;
VE is the estimated vaccine efficacy;
$VE_{predict}$ is the predicted vaccine effectiveness in a new population.

When analysing data from cluster randomized trials, to account for clustering we used the above model, but first divided the terms $p_{v,i}$, $n_{v,i}$, $p_{c,i}$, $n_{c,i}$ by the design effect and rounded to the nearest integer. The design effect for a given study is equal to 1+ (mean.cluster.size -1)*ICC where the ICC is the intra-cluster correlation co-efficient.

S2 Table

Vaccine efficacy analysis: per protocol analysis results for trials in the target age range.

Trial	Vaccine	Age group	Number vaccinated	Number of cases amongst those vaccinated[1]	Number in control group	Number of cases in control group [1]	Efficacy mean (95% CrI)
Loeb et al. 2010 [2]	TIV	36 months to 15 years	502	41	445	79	53.9 (35.2, 69.0)
Clover et al. [3]	TIV	3 to 9 years, US	30	6	33	15	54.4 (9.8, 83.1)
Meta-analysis of TIV studies in children [4]							48.5 (0.7, 68.9)
Predictive TIV effectiveness							46.1 (0.002, 69.7)
Belshe et al. 1998	LAIV	15 to 71 months	849	10	410	74	93.4 (88.3, 96.8)
Belshe et al. 2000	LAIV	26 to 85 months	917	15	441	56	86.9 (78.2, 93.0)
Meta-analysis of LAIV studies in children							90.0 (84.9, 94.2)
Predictive LAIV effectiveness							89.3 (80.0, 94.2)

[1] Including only laboratory-confirmed cases
[2] In this study the numbers in the table are not directly reported, but can be derived from numbers reported in the paper. Our analysis accounts for cluster effects, with the reported intra-cluster correlation co-efficient (ICC) of 0.004.
[3] ICC was not reported for this study, but was assumed to be equal to that reported by Loeb et al (2010).
[4] Two other trials evaluated TIV in children in the target age ranges (Cowling *et al.*, Clin Inf Dis 2012 and Klick *et al.*, Vaccine 2013). These were excluded from the analysis as recommendations to use two doses for children under 9 who had not been vaccinated the previous season were not reported to have been followed.

S3 Table

Vaccine efficacy studies for LAIV used in sensitivity analysis 3, which made use only of studies conducted predominantly in Asia.

Trial	Vaccine	Age group and location	Number vaccinated	Number of cases amongst those vaccinated [1]	Number in control group	Number of cases in control group [1]	Efficacy mean (95% CrI)
Tam et al. 2007 (year 1)	LAIV	12 to <36 months	1653	81	1111	182	70.0 (61.7, 76.9)
Tam et al. 2007 (year 2)[2]	LAIV	12 to <36 months	503	26	494	59	56.0 (33.7, 73.0)
Lum et al. 2010	LAIV	11 to <24 months	765	23	385	32	62.6 (40.0, 79.0)
Meta-analysis of LAIV studies in Asia							67.1 (59.2, 73.7)
Predictive LAIV effectiveness							66.5 (55.9, 73.9)

[1] Including laboratory-confirmed cases of any influenza strain.
[2] Including only those who received the placebo in year 1.

2.4 Observation model

Model fitting assumed that the monthly number of PCR-confirmed cases of influenza A/H1N1, A/H3N2 and B followed a multinomial distribution where the denominator was the number of PCR tests in a given month. The model assumes that the probability of a test for influenza type *j* in month *i* giving a positive result is $p_{ij} = \max(0.01, \sigma^* I_{ij}/(N_i + \Sigma_j\ I_{ij})$. where σ is the sensitivity of the PCR test I_{ij} is the predicted number of ILI cases due to influenza type *j* in month *i*, and N_i is the number of ILI cases not due to influenza in month *i* . The contribution to I_{ij} from age group *k* is equal to the product of the predicted number of new infections with influenza type *j* in month *i* in age group *k* and the age-specific probability that each infection leads to a reported ILI case, ρ_i. These reporting probabilities (which we assume to be common to all influenza types) in turn are given as the ratio of the estimated number of ILI cases due to influenza in age group *k*, and the number of influenza infections predicted by the model occurring age group *k.* The estimated number of ILI cases due to influenza in a given age group was obtained by subtracting from the observed number of ILI cases the total number of ILI cases not due to influenza. The latter was taken as the sum of the expected number of ILI cases *not* caused by influenza A/H1N1, A/H3N2 or B in each month *i=1..12* (N_i)*.* Such infections may be caused by a variety of pathogens (e.g other influenza viruses, RSV, bacterial pathogens etc). In the absence of systematic surveillance data for such pathogens, we assumed that N_i varied by age group but was constant over each 12 month period within an age group; the age and year-specific value was then taken as the minimum monthly number of ILI cases in a given year and age group. The lower bound of p_{ij} of 0.01 was imposed primarily for numerical

reasons (to avoid multinomial probabilities of zero) and corresponds to the assumption that a minimum of 1% of swabs taken from ILI cases would be confirmed positive for a given influenza subtype even in the absence of circulating influenza virus (for example due to imported cases or false positives).

2.5 Health outcome and cost models

Age- type- and year-specific mortality due to influenza in Thailand was estimated using a Bayesian implementation of a previously described regression approach (Goldstein *et al.*, 2012), incorporating a number of refinements (Cooper *et al*, 2015). We used 10,000,000 iterations of each model, discarding the first 1,000,000 as burn-in. Summaries of posterior distributions obtained using this approach are shown below. In the decision-model we approximated these distributions using normal distributions with the same mean and variance, as the computational burden precluded simultaneous inference in a joint model. Any samples from these distributions with negative values were replaced with zero.

S4 Table: Age- subtype and year-specific seasonal influenza mortality estimated in Thailand. Figures give estimated number deaths per 100,000 person years in each age group.

Influenza type and year	Age <18 yrs		Age 18-59 yrs		Age >59 yrs	
	mean	SD	mean	SD	mean	SD
B 2005-6	1.40	3.20	-1.21	1.54	61.42	32.82
B 2006-7	1.37	2.89	-0.84	0.94	31.64	25.48
B 2007-8	4.19	5.17	-2.84	1.99	65.34	58.03
B 2008-9	2.95	3.86	-1.71	1.73	37.66	58.69
H1N1 2005-6	0.16	0.40	0.24	0.27	11.58	10.97
H1N1 2006-7	0.80	2.88	1.83	1.10	44.95	41.68
H1N1 2007-8	0.33	2.04	1.03	0.89	134.00	61.71
H1N1 2008-9	0.43	2.39	2.33	1.21	1.11	58.69
H3N2 2005-6	-1.45	3.66	0.39	1.60	2.72	34.32
H3N2 2006-7	2.21	2.57	1.68	1.08	21.46	27.41
H3N2 2007-8	-0.33	2.92	0.64	1.59	-3.15	34.22
H3N2 2008-9	1.79	3.25	2.49	1.72	15.89	33.15

S5 Table. Health outcome components.

Parameter	Prior distribution; mean [95% CI]	Sources
Number of school days absenteeism for non-hospitalized children aged 0-17 y	Gamma 3.3 [0.30, 9.75]	Simmerman et al., 2006
Number of work days absenteeism for non-hospitalized adults aged ≥18 y	Gamma 4.5 [0.96, 10.78]	Simmerman et al., 2006
Number of recovery days at home for hospitalized patients	Gamma 3.00 [0.08, 11.07]	(a)
Number of parents/relatives needed for attending medical care		
OPD visit		
0-17 y	1	(a)
≥18 y	0	(a)
Hospitalization		
0-17 y	1	(a)
≥18 y	1	(a)
Length of hospital stay (days)	Normal	
<2 y	3.44 [2.87, 4.01]	(b)
2-5 y	3.52 [3.23, 3.81]	(b)
6-11 y	3.03 [2.74, 3.32]	(b)
12-17 y	2.76 [2.64, 2.88]	(b)
18-59 y	2.59 [2.51, 2.67]	(b)
≥60 y	3.53 [3.23, 3.82]	(b)
Probability of medically-attended Guillain-Barré syndrome after influenza infection		
<19 y	Beta $5x10^{-8}$ [$9x10^{-9}$,$1x10^{-7}$]	Dieleman et al. 2007
19-59 y	Beta $1.9x10^{-7}$ [$1.7x10^{-7}$,$2.1x10^{-7}$]	Dieleman et al. 2007
≥60 y	Beta $3.6x10^{-7}$ [$2.7x10^{-7}$,$4.6x10^{-7}$]	Dieleman et al. 2007
Length of hospital stay for an episode of Guillain-Barré syndrome (days)	Gamma 16.78 [13.28,20.68]	Stow et al. 2009
Clinical data (per case of vaccination)		
Probability of medically-attended vaccine-related adverse events		
Injection site reaction	Beta	
2 y	0.003 [$7.4x10^{-5}$,0.011]	Neuzil et al. 2001
3-4 y	0.002 [$5.0x10^{-5}$,0.0074]	Neuzil et al. 2001
5-11 y	0.001 [$2.5x10^{-5}$,0.0037]	Neuzil et al. 2001
12-17 y	0.0003 [$7.5x10^{-6}$,0.0011]	Neuzil et al. 2001
Systemic reaction (fever)	Beta	
2 y	0.011 [0.00026, 0.041]	Belshe et al. 2000
3-4 y	0.009 [0.00022, 0.033]	Belshe et al. 2000
5-11 y	0.004 [0.00001, 0.015]	Belshe et al. 2000
12-17 y	0.0003 [$7.5x10^{-6}$,0.0011]	Belshe et al. 2000
Anaphylaxis	0.000001 [$2.5x10^{-8}$,$3.7x10^{-6}$]	Belshe et al. 2000
Relative risk for Guillain-Barré syndrome after seasonal influenza vaccination	Gamma 1.5 [0.6 to 3.9]	Dieleman et al. 2007
Length of hospital stay for an episode of anaphylaxis (days)	Gamma 1.71 [1.45, 2.00]	(b)
Disability weight (per episode)	Beta	
Symptomatic influenza infection	0.005 [0.002, 0.009]	Prosser et al. 2005
Influenza infection with OP visit	0.0078 [0.00019, 0.029]	Lugner et al. 2012
Influenza infection with hospitalization	0.0217 [0.00049, 0.080]	Lugner et al. 2012
Anaphylaxis	0.020 [0.006, 0.041]	Prosser et al. 2005
Guillain-Barré syndrome	0.141 [0.092, 0.199]	Prosser et al. 2005

(a) The authors' assumption.

(b) The Central office for Healthcare Information, Bangkok. Dataset of in-patient service utilization of year 2009.

S6 Table. Cost components.

Parameter	Prior distribution mean [95% CI]	Distribution	Sources
Influenza-related costs (Baht)			
OTC medication for pain and fever relief	Gamma		
< 2 y	20.25 [17.49, 23.21]	Gamma	(a)
2-5 y	14.30 [11.99, 16.81]	Gamma	(a)
6-11 y	26.92 [21.57,32.85]	Gamma	(a)
≥12 y	4.2 [3.36,5.13]	Gamma	(a)
Direct medical cost per OPD visit	373 [238, 538]	Gamma	Simmerman et al. 2006
Direct medical cost per hospitalization	Gamma		
< 2 y	7,448 [5,204, 10,088]	Gamma	(b)
2-5 y	7,122 [6381, 7882]	Gamma	(b)
6-11 y	6,466 [4,518,8,757]	Gamma	(b)
12-17 y	4,756 [4,446, 5,077]	Gamma	(b)
18-59 y	13,563 [12,644,14,514]	Gamma	(b)
≥60 y	8,923 [6,922, 11,174]	Gamma	(b)
Food cost per OPD visit	35.1 [14.7 to 57.9]	Gamma	Riewpaiboon 2011
Transportation cost per OPD visit	101.2 [59.3 to 157.9]	Gamma	Riewpaiboon 2011
Average daily income	371		World Bank
Vaccine-related costs (Baht)			
TIV, per dose	201		(c)
LAIV, per dose	322		(d)
Vaccine supply chain and logistics, per dose	18.5 [12.1, 26.2]	Gamma	PATH WHO 2011
Hospital-based vaccine administration cost, per dose	117.1 [85.3, 153.8]	Gamma	Riewpaiboon 2011
Additional cost of school-based vaccine administration, per dose	67.0 [1.7, 247.2]	Gamma	Riewpaiboon 2011
Direct medical cost for vaccine-related adverse events, per episode			
Injection site reaction	202.6 [101.2 to 287.8]	Gamma	Riewpaiboon 2011
Anaphylaxis			
< 6 y	1,215 [30.8, 4,482]	Gamma	
6-11 y	1,966 [1,780, 2,161]	Gamma	(b)
12-17 y	3,129 [2,303, 4,079]	Gamma	(b)
18-59 y	6,326 [4,510, 8,445]	Gamma	(b)
≥60 y	5,302 [3,606, 7,320]	Gamma	(b)
Guillain-Barré syndrome			
< 6 y	134,675 [38,894, 288,853]	Gamma	(b)
6-11 y	177,023 [89,847, 293,264]	Gamma	(b)
12-17 y	159,129 [82,934, 259,737]	Gamma	(b)
18-59 y	196,042 [150,031, 248,113]	Gamma	(b)
≥60 y	192,543 [141,332, 251,550]	Gamma	(b)
Injection site reaction	202.6 [101.2 to 287.8]	Gamma	(b)

(a) Center of Essential Information for All Health Officers, Ministry of Public Health, Thailand. Reference government purchasing price of drug and medical supplies. [cited 2013, Feb 15]; Available from: http://dmsic.moph.go.th/price.htm.

(b) The Central Office for Healthcare Information, Bangkok. Dataset of in-patient service utilization of year 2009.

(c) Average government purchasing price per dose, 2009-2012 (Ministry of Public Health, Thailand).

(d) Assumed to be 1.6 times higher than TIV.

2.7 Materials and procedure for elicitation of expert opinions

We used materials and protocols from the Sheffield Elicitation Framework (SHELF version 2, available from http://tonyohagan.co.uk/shelf) to elicit priors from a panel of subject-area experts, making use of both individual and group elicitation. The panel of experts comprised of four epidemiologists (all active researchers in the area) and six clinicians (all with relevant clinical experience in Thailand). Elicitation made use of the the *rpanel* package in *R* version 1.12.2 (Bowman *et al*., 2007; R Development Core Team, 2010). An example elicitation form is shown in Supplementary Figure 2.

S2 Figure: Example of an elicitation form.

**Quantity**	Attack rate (AR) of seasonal influenza
**Definition**	Proportion of persons in a population who experience influenza
**Evidence**	The Israel study estimated that the AR of seasonal influenza was between 5% to 15%. (ref.Barnea O, et al. Math BiosciEng2011)<br>The AR of seasonal influenza in the UK and France was between 10% to 20% (ref.Truscott J, et al. J R Soc Interface 2012)

**What percent of people in each age group have clinical cases of seasonal influenza**		**Age groups**				
		**0-23 months**	**2-4years**	**5-14 years**	**15-59 years**	**>=60 years**
**Plausible range**	A. Realistically, what do you think the lowest value could be?					
	A. Realistically, what do you think the highest value could be?					
**Median**	A. What would be the most likely number? (considering range fromA toB)					
**Lower and upper quartiles**	A. What would be the lower quartile? (considering range fromA toC)					
	A. What would be the lower quartile? (considering range fromC toB)					

3. **Supplementary Results**

3.1 Additional results from the base case model

S7 Table. Posterior distributions from base case analysis.

Parameter	Posterior mean (95% CrI)
R_0 for influenza A/ H1N1	2005-6: 0.52 (0.12, 1.07)<br>2006-7: 1.23 (1.17, 1.29)<br>2007-8: 1.16 (1.11, 1.22)<br>2008-9: 1.12 (1.08 1.17)
R_0 for influenza A/ H3N2	2005-6: 1.19 (1.13, 1.26)<br>2006-7: 1.18 (1.14, 1.24)<br>2007-8: 1.15 (1.10, 1.20)<br>2008-9: 1.15 (1.10, 1.20)
R_0 for influenza B	2005-6: 1.14 (1.10, 1.20)<br>2006-7: 1.16 (1.11, 1.22)<br>2007-8: 1.18 (1.13, 1.23)<br>2008-9: 1.15 (1.10, 1.21)
Serial interval influenza A/H1N1	2005-6: 2.50 (2.31, 2.71)<br>2006-7: 2.53 (2.34, 2.73)<br>2007-8: 2.40 (2.21, 2.60)<br>2008-9: 2.52 (2.32, 2.72)
Serial interval influenza A/H3N2	2005-6: 2.51 (2.33, 2.72)<br>2006-7: 2.52 (2.33, 2.72)<br>2007-8: 2.50 (2.32, 2.70)<br>2008-9: 2.53 (2.34, 2.73)
Serial interval influenza B	2005-6: 2.50 (2.31, 2.70)<br>2006-7: 2.48 (2.29, 2.68)<br>2007-8: 2.62 (2.42, 2.83)<br>2008-9: 2.48 (2.29, 2.68)
Probability of immunity at the start of influenza season	
< 2 y	A/H1N1 2005-9: 0.10 (0.05, 0.17)<br>A/H3N2 2005-9: 0.10 (0.05, 0.17)<br>B 2005-9: 0.10 (0.05, 0.17)
2-5 y	A/H1N1 2005-9: 0.13 (0.06, 0.21)<br>A/H3N2 2005-9: 0.13 (0.06, 0.21)<br>B 2005-9: 0.13 (0.06, 0.21)
6-11 y	A/H1N1 2005-9: 0.17 (0.14, 0.19)<br>A/H3N2 2005-9: 0.17 (0.14, 0.19)<br>B 2005-9: 0.17 (0.14, 0.19)

Parameter	Posterior mean (95% CrI)
12-17 y	A/H1N1 2005-9: 0.17 (0.14, 0.19)<br>A/H3N2 2005-9: 0.17 (0.14, 0.19)<br>B 2005-9: 0.17 (0.14, 0.19)
18-59 y	A/H1N1 2005-9: 0.20 (0.13, 0.27)<br>A/H3N2 2005-9: 0.19 (0.13, 0.26)<br>B 2005-9: 0.19 (0.13, 0.26)
≥60 y	A/H1N1 2005-9: 0.19 (0.10, 0.29)<br>A/H3N2 2005-9: 0.19 (0.10, 0.29)<br>B 2005-9: 0.19 (0.10, 0.29)
Proportion initially infected	Influenza A/H1N1<br>2005-6: 0.001 (1×10^{-6}, 0.007)<br>2006-7: 1×10^{-4} (7×10^{-5}, 3×10^{-4}),<br>2007-8: 3×10^{-5} (2×10^{-5}, 5×10^{-5}),<br>2008-9: 1×10^{-4} (6×10^{-5}, 2×10^{-4}),<br><br>Influenza A/H3N2<br>2005-6: 0.001 (5×10^{-4}, 0.002)<br>2006-7: 1×10^{-6} (1×10^{-7}, 3×10^{-6})<br>2007-8: 8×10^{-5} (6×10^{-5}, 1×10^{-4}),<br>2008-9: 3×10^{-4} (2×10^{-4}, 4×10^{-4}),<br><br>Influenza B<br>2005-6: 7×10^{-5} (4×10^{-5}, 1×10^{-4}),<br>2006-7: 3×10^{-5} (1×10^{-5}, 6×10^{-5}),<br>2007-8: 4×10^{-6} (2×10^{-6}, 6×10^{-6}),<br>2008-9: 1×10^{-4} (9×10^{-5}, 2×10^{-4}),
Sensitivity of laboratory confirmation test	0..34 (0.25, 0.44)
Probability of symptoms given infection	0.68 (0.59, 0.76)

S3 Figure. Estimated numbers of symptomatic cases of seasonal influenza infection under base case assumptions.

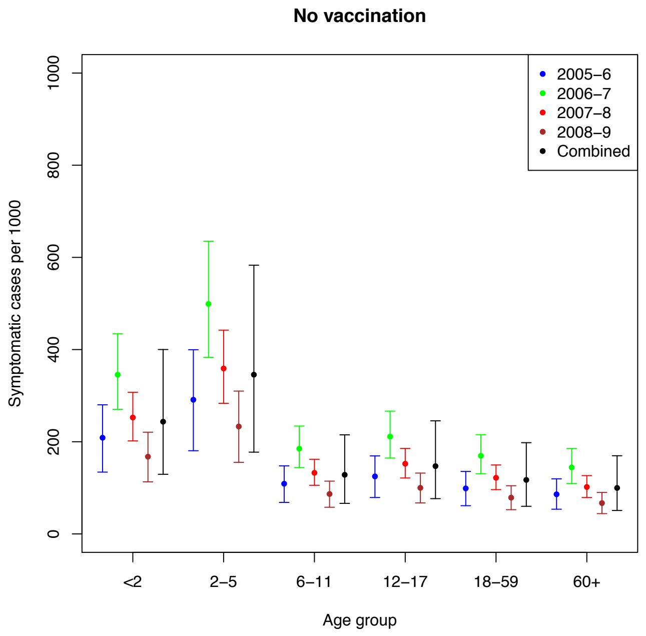

S4 Figure. Symptomatic cases averted under base case assumptions.

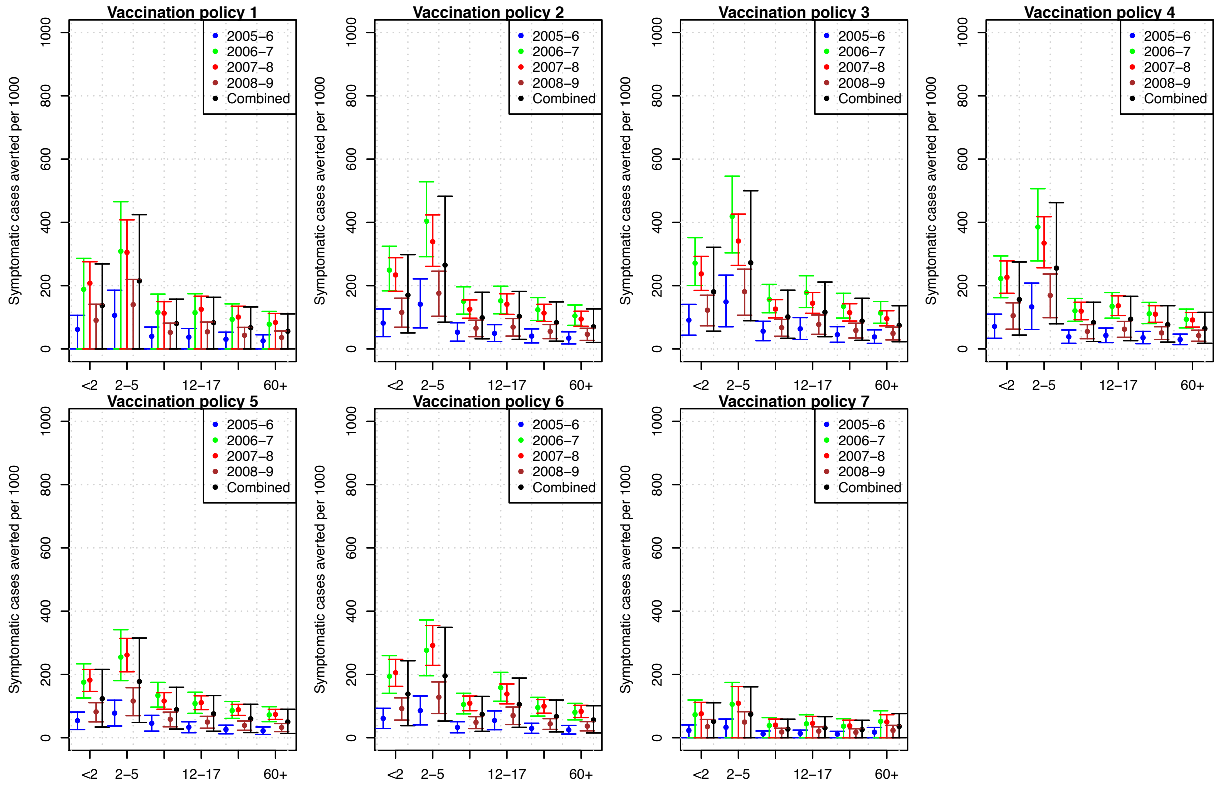

S5 Figure. Estimated number of deaths by age group due to influenza in the absence of vaccination against seasonal influenza under base case assumptions.

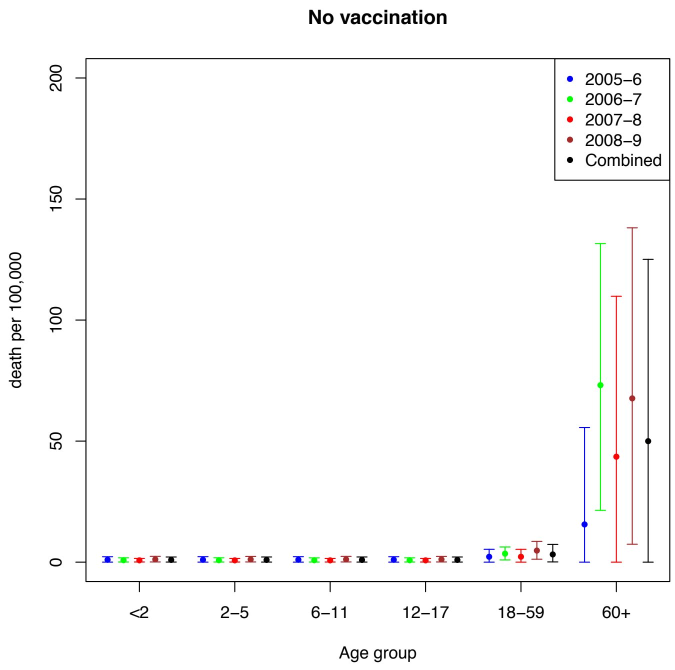

S6 Figure. Deaths averted under base case assumptions.

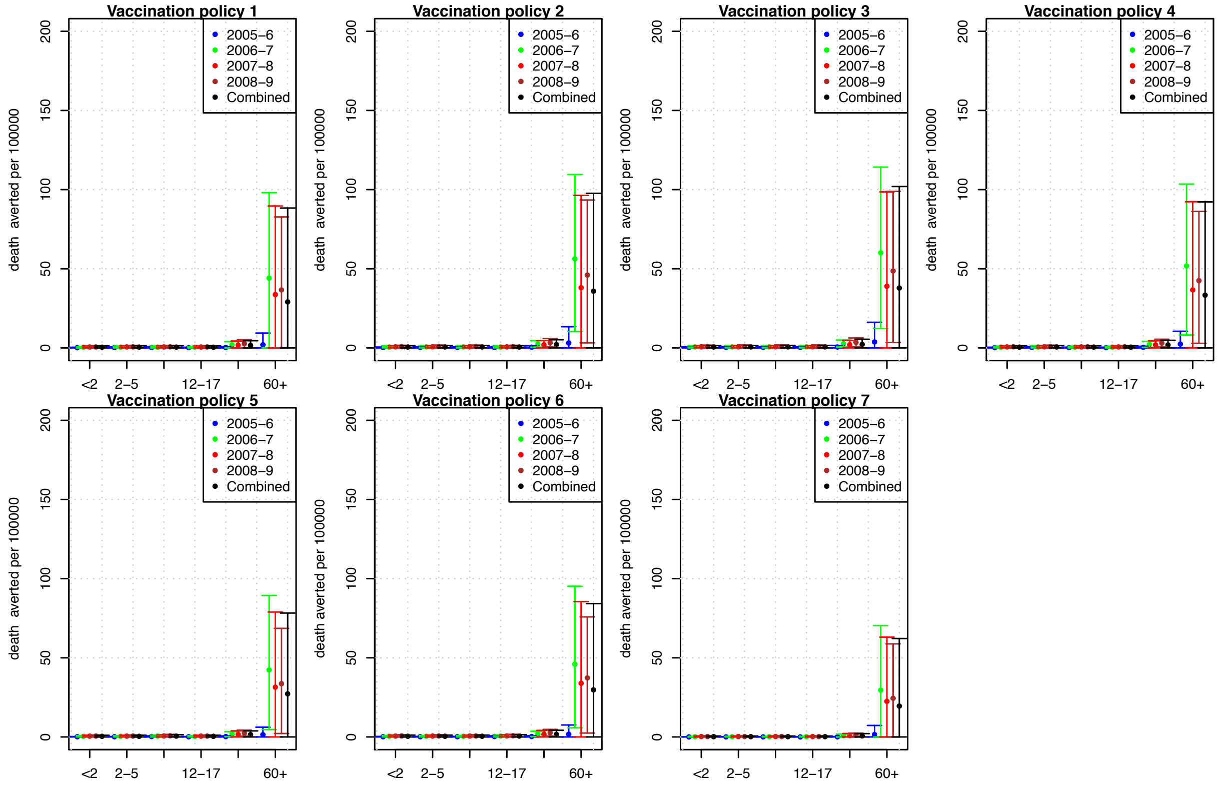

S7 Figure. Expected value of perfect information under base case assumptions.

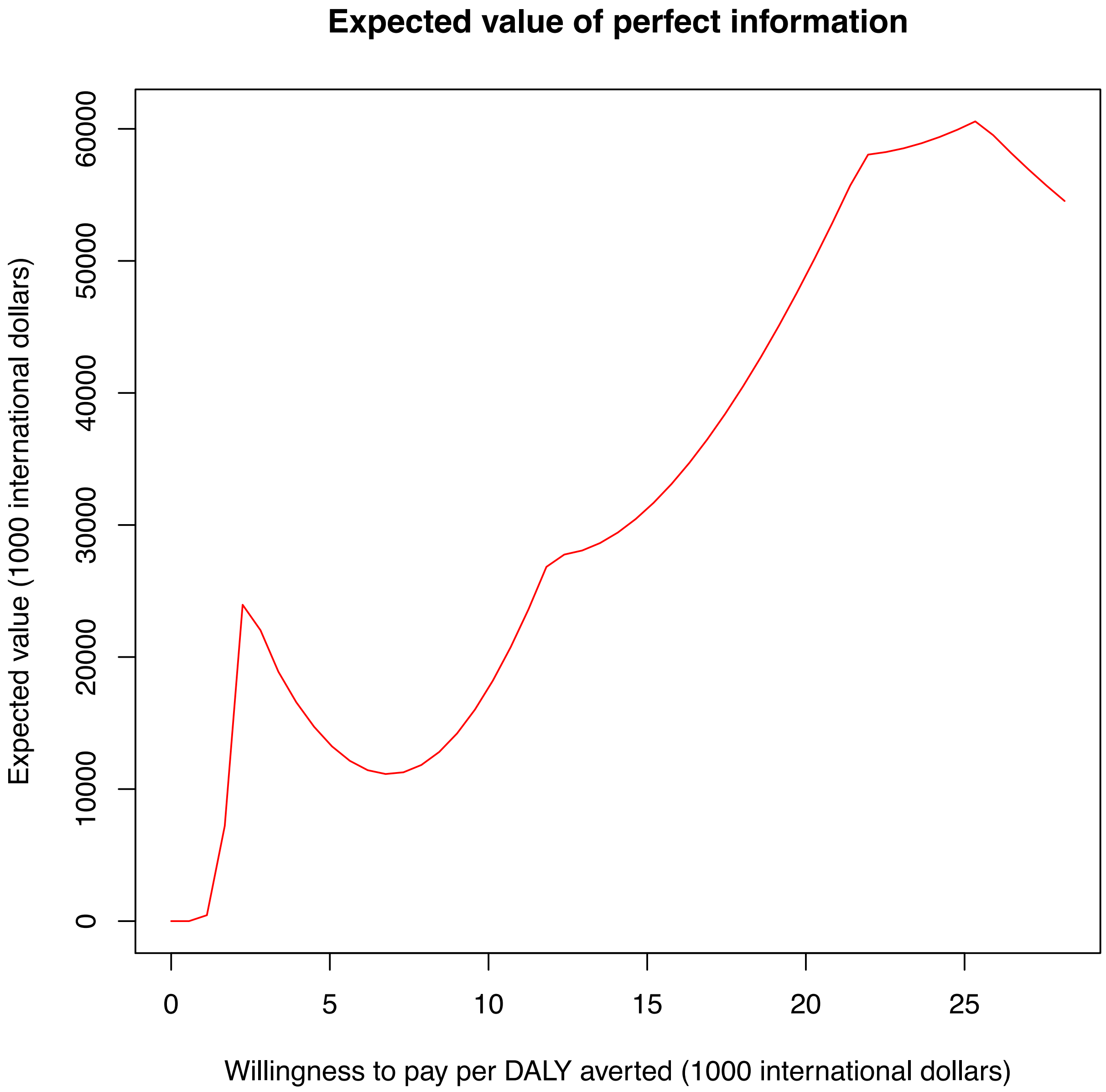

S8 Table. Expected value of partial perfect information (EVPPI) under base case model at a cost-effectiveness threshold of I$ 10,000 per DALY averted.

Parameter		EVPPI (international dollars)
Initial R of A/H1N1		4.9 million
Initial R of A/H3N2		0
Initial R of B		0
Serial interval A/H1N1		0
Serial interval A/H3N2		0
Serial interval B		0
PCR test sensitivity		5.5 million
Proportion immune A/H1N1		
	age group 1	0
	age group 2	0
	age group 3	0
	age group 4	0
	age group 5	0
	age group 6	0
Proportion immune A/H3N2		
	age group 1	0
	age group 2	0
	age group 3	0
	age group 4	0
	age group 5	0
	age group 6	0
Proportion immune B		
	age group 1	0
	age group 2	0
	age group 3	0
	age group 4	0
	age group 5	0
	age group 6	0

3.2 Additional results from sensitivity analysis 1 (contact matrix using physical contacts only).

S9 Table. Posterior distributions from sensitivity analysis 1 (contact matrix using physical contacts only).

Parameter	Posterior mean (95% CrI)
R_0 for influenza A/ H1N1	2005-6: 0.47 (0.12, 0.95)<br>2006-7: 1.11 (1.06, 1.16)<br>2007-8: 1.04 (1.00, 1.09)<br>2008-9: 1.01 (0.98, 1.05)
R_0 for influenza A/ H3N2	2005-6: 1.07 (1.02, 1.13)<br>2006-7: 1.07 (1.03, 1.11)<br>2007-8: 1.03 (1.00, 1.08)<br>2008-9: 1.03 (0.99, 1.07)
R_0 for influenza B	2005-6: 1.03 (0.99, 1.07)<br>2006-7: 1.04 (1.01, 1.09)<br>2007-8: 1.07 (1.03, 1.11)<br>2008-9: 1.03 (1.00, 1.08)
Serial interval influenza A/H1N1	2005-6: 2.50 (2.31,2.71)<br>2006-7: 2.53 (2.34, 2.74)<br>2007-8: 2.40 (2.21, 2.60)<br>2008-9: 2.52 (2.32, 2.72)
Serial interval influenza A/H3N2	2005-6: 2.51 (2.33, 2.71)<br>2006-7: 2.52 (2.32, 2.73)<br>2007-8: 2.50 (2.32, 2.70)<br>2008-9: 2.53 (2.34, 2.73)
Serial interval influenza B	2005-6: 2.50 (2.31,2.71)<br>2006-7: 2.48 (2.29, 2.68)<br>2007-8: 2.62 (2.42, 2.84)<br>2008-9: 2.48 (2.30, 2.68)
Probability of immunity at the start of influenza season	
< 2 y	A/H1N1 2005-9: 0.10 (0.05, 0.17)<br>A/H3N2 2005-9: 0.10 (0.05, 0.17)<br>B 2005-9: 0.10 (0.05, 0.18)
2-5 y	A/H1N1 2005-9: 0.13 (0.07, 0.21)<br>A/H3N2 2005-9: 0.13 (0.06, 0.20)<br>B 2005-9: 0.13 (0.07, 0.21)
6-11 y	A/H1N1 2005-9: 0.17 (0.14, 0.19)<br>A/H3N2 2005-9: 0.17 (0.14, 0.19)<br>B 2005-9: 0.17 (0.14, 0.19)
12-17 y	A/H1N1 2005-9: 0.17 (0.14, 0.19)<br>A/H3N2 2005-9: 0.17 (0.14, 0.19)<br>B 2005-9: 0.17 (0.14, 0.19)

Parameter	Posterior mean (95% CrI)
18-59 y	A/H1N1 2005-9: 0.20 (0.13, 0.27)<br>A/H3N2 2005-9: 0.19 (0.13, 0.26)<br>B 2005-9: 0.19 (0.13, 0.26)
≥60 y	A/H1N1 2005-9: 0.19 (0.10, 0.30)<br>A/H3N2 2005-9: 0.19 (0.10, 0.30)<br>B 2005-9: 0.19 (0.10, 0.29)
Proportion initially infected	Influenza A/H1N1<br>2005-6: 0.001 (4×10^{-7}, 0.006)<br>2006-7: 0.0001 (5×10^{-5}, 2×10^{-4})<br>2007-8: 2×10^{-5} (2×10^{-5}, 4×10^{-5})<br>2008-9: 8×10^{-5} (5×10^{-5}, 1×10^{-4})<br><br>Influenza A/H3N2<br>2005-6: 0.0008 (0.0004, 0.001)<br>2006-7: 9×10^{-7} (7×10^{-8}, 3×10^{-6})<br>2007-8: 7×10^{-5} (5×10^{-5}, 9×10^{-5})<br>2008-9: 8×10^{-5} (5×10^{-5}, 1×10^{-4})<br><br>Influenza B<br>2005-6: 6×10^{-5} (3×10^{-5}, 9×10^{-5})<br>2006-7: 3×10^{-5} (8×10^{-6}, 5×10^{-5})<br>2007-8: 3×10^{-6} (1×10^{-6}, 5×10^{-6})<br>2008-9: 1×10^{-4}(8×10^{-5}, 2×10^{-4})
Sensitivity of laboratory confirmation test	0.34 (0.25, 0.45)
Probability of symptoms given infection	0.68 (0.59, 0.76)

S10 Table. Model outcomes showing mean (95% CrI) under sensitivity analysis 1.

Total cost (million int $)	Outcomes (1000 cases): Symptoms	Outcomes (1000 cases): Outpatient visits	Outcomes (1000 cases): In-patient visits	Outcomes (1000 cases): Deaths	Comparison with no childhood vaccination: Incremental cost (million int $)	Comparison with no childhood vaccination: DALYs averted (1000s)	Comparison with no childhood vaccination: ICER
No childhood vaccination							
20 (16,24)	7241 (3804, 12100)	5.3 (0.0,7.3)	3.3 (1.2,8.0)	4.3 (0.6, 9.2)			
Policy 1: Vaccinate those aged 2-11 years with TIV							
237 (213, 287)	2315 (317, 7255)	2.2 (0.0,6.7)	0.7 (0.3,2.5)	1.4 (0.1, 4.5)	205 (180, 245)	53 (1, 107)	3873
Policy 2: Vaccinate those aged 2-11 years with LAIV							
296 (272, 345)	1562 (250, 3777)	1.6 (0.0,4.4)	0.4 (0.3, 0.8)	1.9 (0.1, 2.7)	264 (239, 313)	61 (13, 119)	4306
Policy 3: Vaccinate those aged 2-17 years with LAIV							
392 (359, 450)	1419 (225, 3527)	1.5 (0.0,4.2)	0.4 (0.2, 0.7)	0.9 (0.1, 2.6)	360 (326, 418)	63 (15, 123)	5717
Policy 4: Vaccinate those aged 2-5 years with LAIV							
184 (175, 195)	1822 (299, 4163)	1.9 (0.0,4.7)	0.5 (0.3, 0.8)	1.2 (0.1, 3.0)	153 (141, 165)	58 (12, 114)	2622
Policy 5: Vaccinate those aged 6-11 years with LAIV							
126 (107, 175)	2689 (654, 5185)	2.5 (0.0,5.3)	0.8 (0.5, 1.1)	1.7 (0.2, 3.9)	94 (73, 144)	49 (9, 96)	1923
Policy 6: Vaccinate those aged 12-17 years with LAIV							
111 (95, 152)	2968 (915, 5466)	2.7 (0.0,5.4)	1.0 (0.5, 1.5)	1.8 (0.2,4.1)	79 (61, 122)	46 (8, 91)	1719
Policy 7: Increase vaccination coverage in those aged 60 years and over with TIV							
86 (77, 96)	6500 (3401, 11077)	4.8 (0, 7.3)	2.9 (1.1, 7.4)	3.3 (0.5, 7.3)	54 (47,63)	12 (0, 30)	4500

S8 Figure. Model fits to laboratory-confirmed influenza surveillance data under sensitivity analysis 1. Monthly numbers of PCR-confirmed influenza cases (circles) and model predictions: median (broken line), and 95%, 90% and 80% (gray shading) prediction intervals for the expected number of cases.

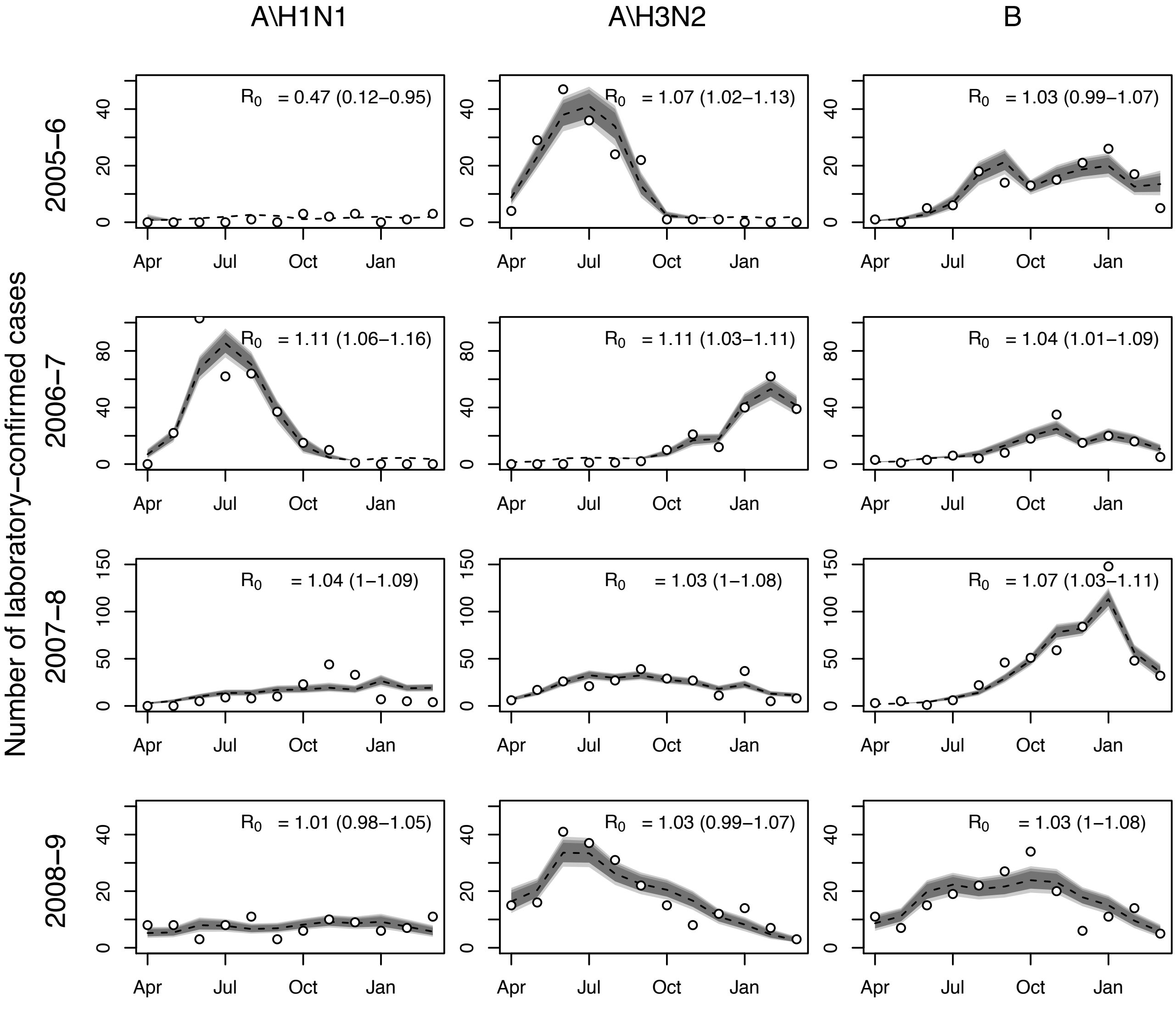

S9 Figure. Cost-effective Acceptability Curves under sensitivity analysis 1.

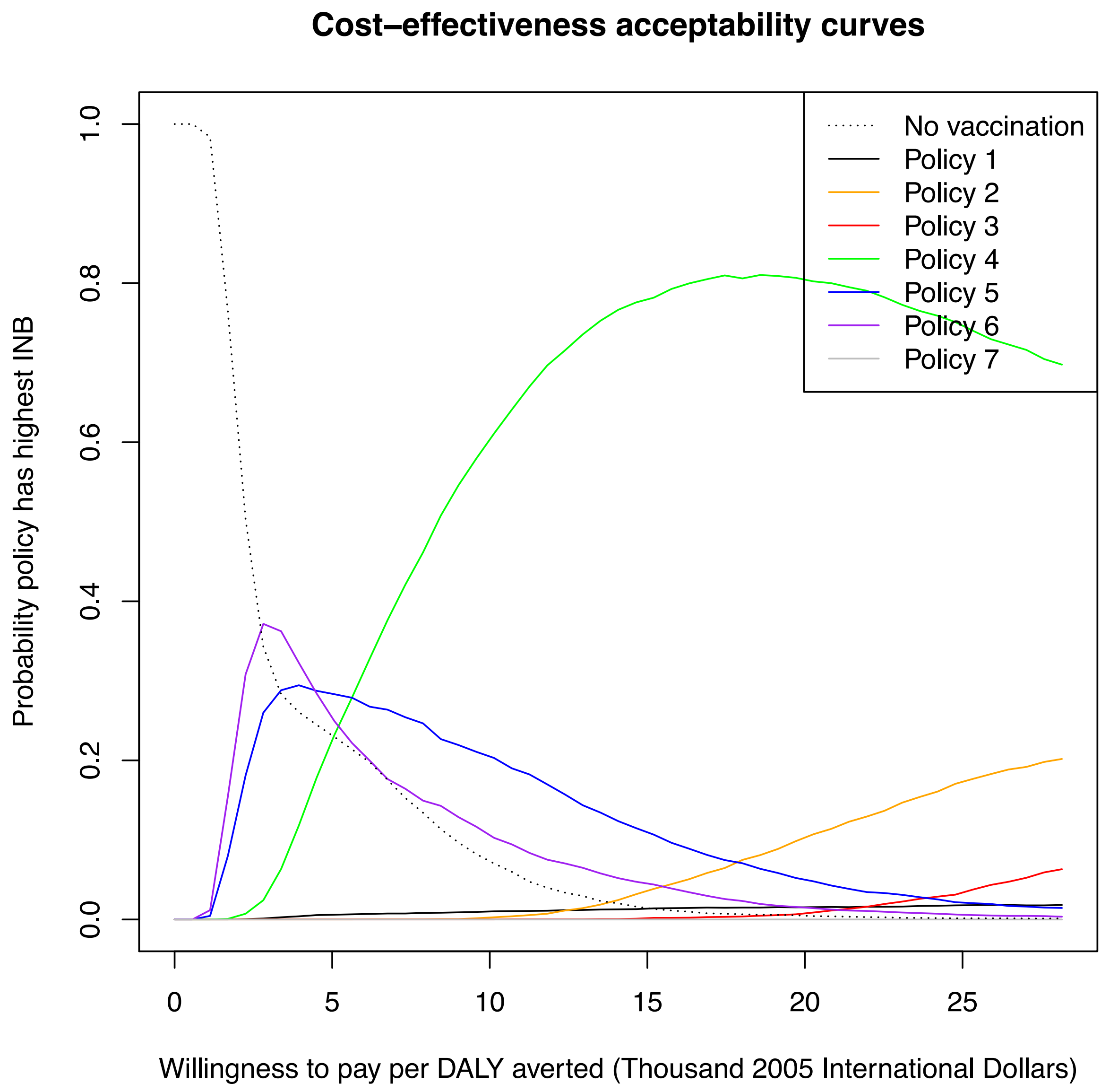

S10 Figure. Estimated numbers of symptomatic cases of seasonal influenza infection under sensitivity analysis 1.

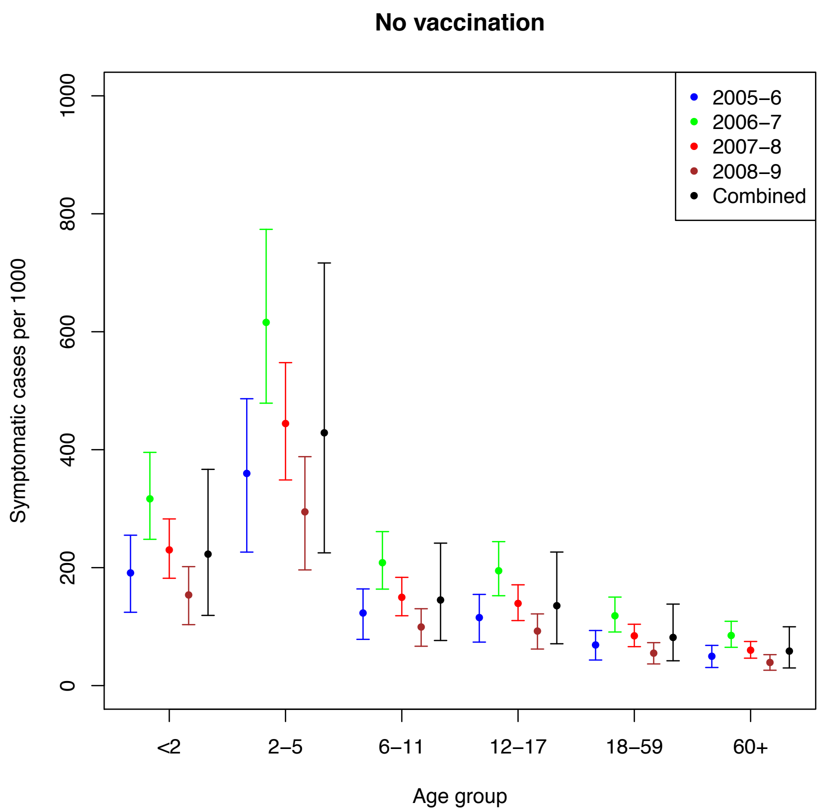

S11 Figure. Symptomatic cases averted under sensitivity analysis 1

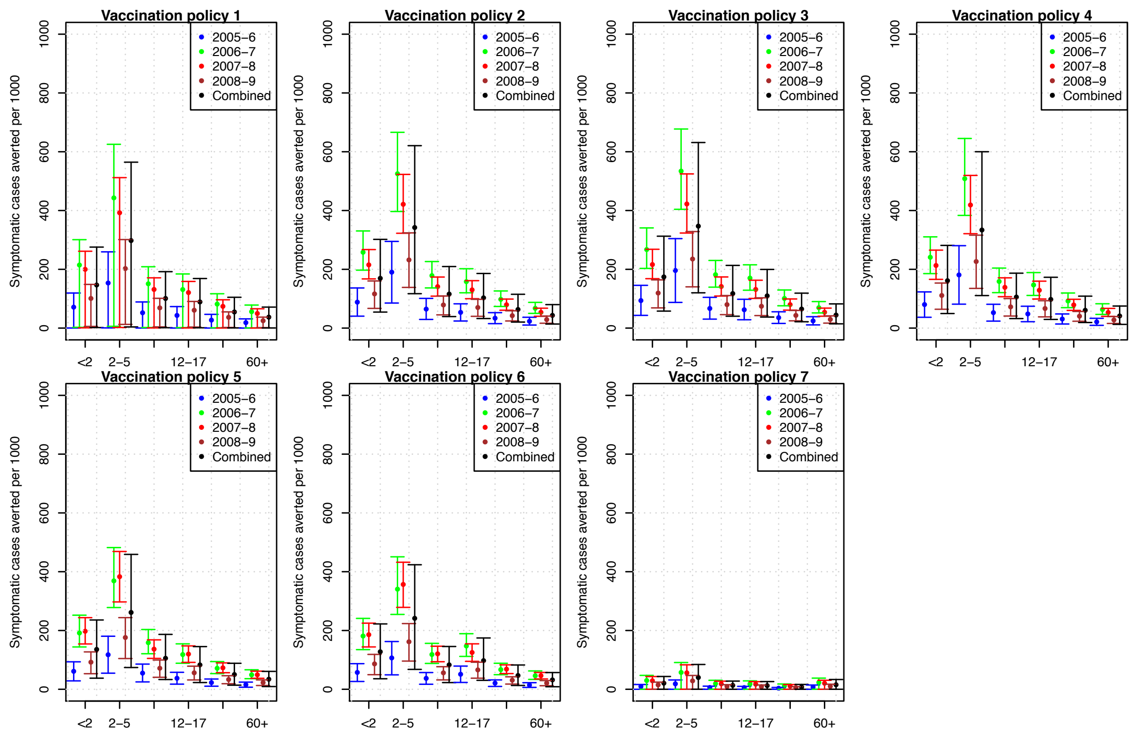

S12 Figure. Estimated number of deaths by age group due to influenza in the absence of vaccination against seasonal influenza under sensitivity analysis 1.

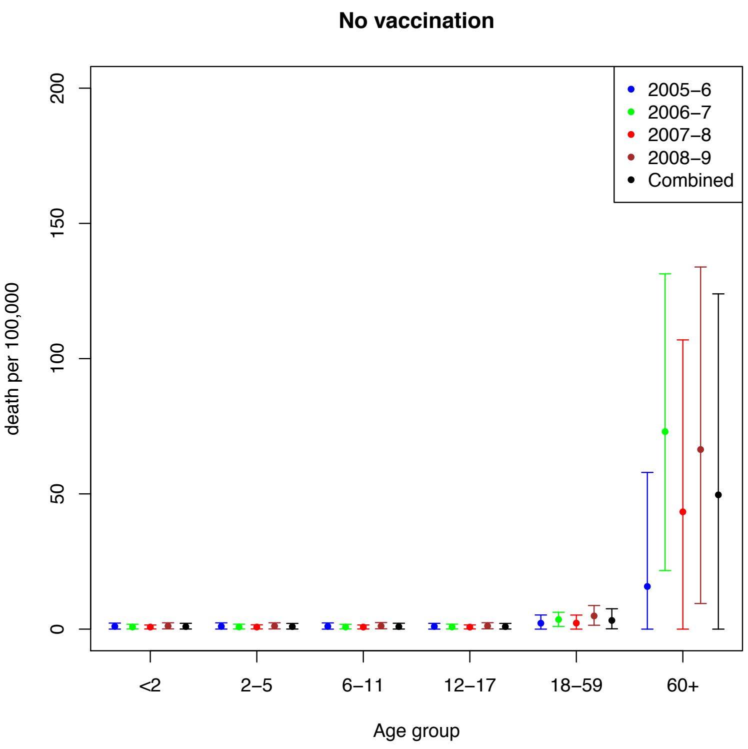

S13 Figure. Deaths averted under sensitivity analysis 1.

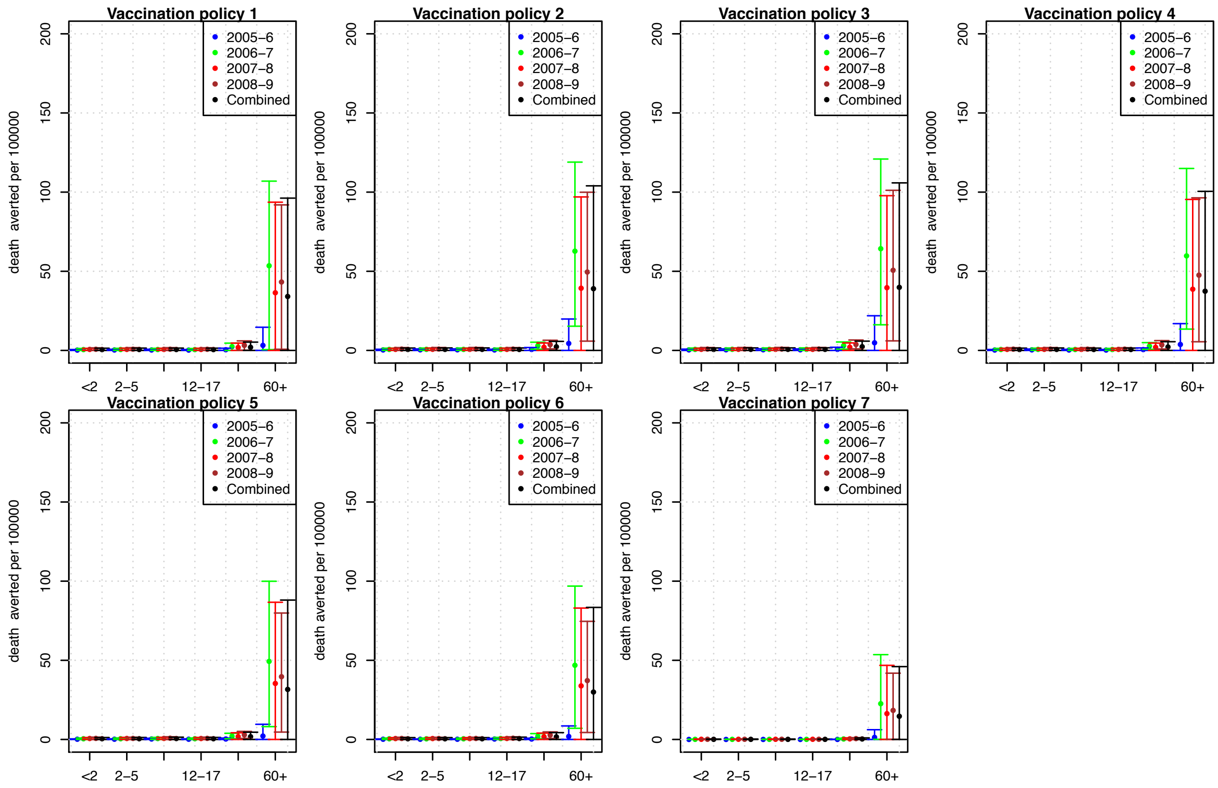

S14 Figure. DALYs averted as a result of direct and indirect vaccine effects under sensitivity analysis 1.

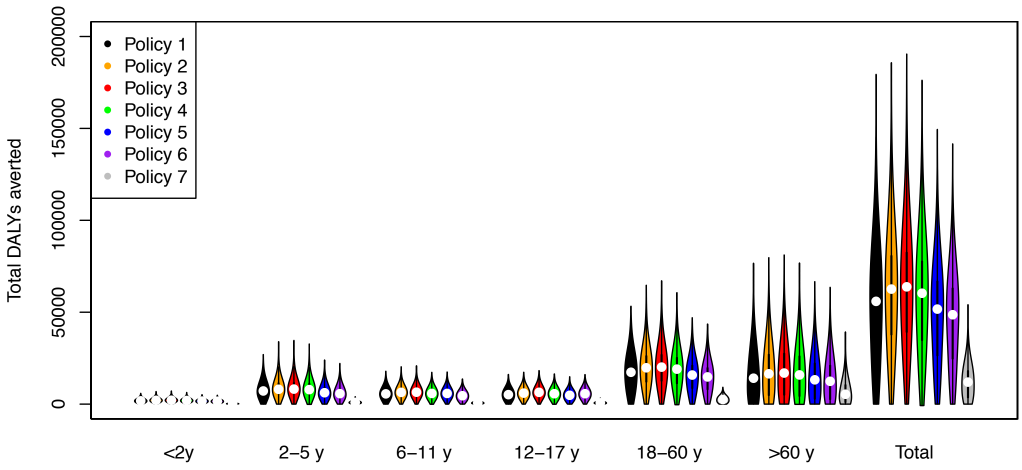

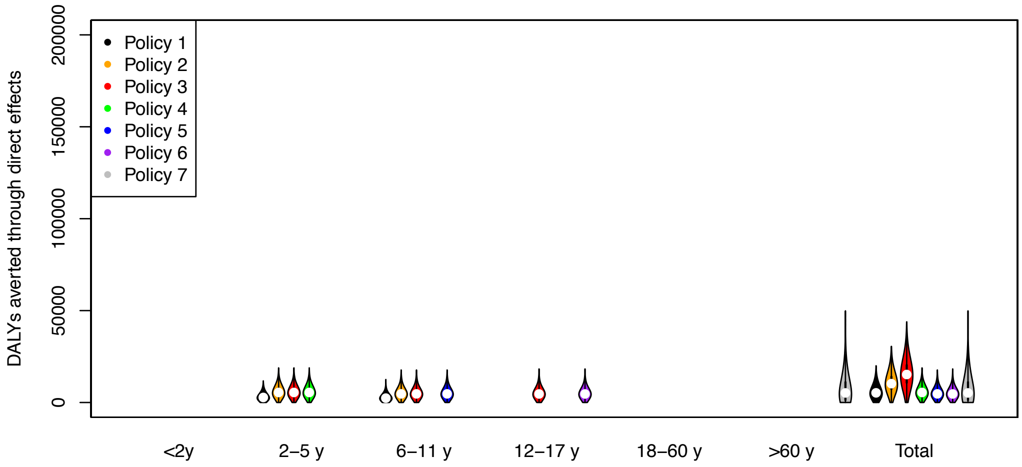

S15 Figure. Expected value of perfect information under sensitivity analysis 1.

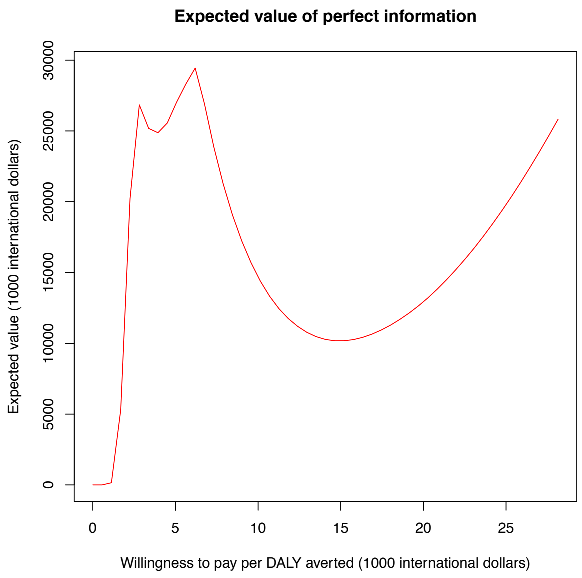

3.2 Additional results from sensitivity analysis 2

S11 Table. Posterior distributions from sensitivity analysis 2 (double probability of immunity prior to epidemic start).

Parameter	Posterior mean (95% CrI)
R_0 for influenza A/ H1N1	2005-6: 0.62 (0.13, 1.31)<br>2006-7: 1.56 (1.44, 1.73)<br>2007-8: 2.16 (1.97, 2.34)<br>2008-9: 1.45 (1.32, 1.59)
R_0 for influenza A/ H3N2	2005-6: 1.49 (1.34, 1.67)<br>2006-7: 1.50 (1.37, 1.66)<br>2007-8: 1.53 (1.40, 1.67)<br>2008-9: 1.46 (1.33, 1.61)
R_0 for influenza B	2005-6: 1.46 (1.31, 1.63)<br>2006-7: 1.60 (1.42, 1.82)<br>2007-8: 1.45 (1.36, 1.56)<br>2008-9: 1.53 (1.39, 1.69)
Serial interval influenza A/H1N1	2005-6: 2.50 (2.32,2.70)<br>2006-7: 2.53 (2.36, 2.72)<br>2007-8: 2.24 (2.34, 2.60)<br>2008-9: 2.52 (2.34, 2.71)
Serial interval influenza A/H3N2	2005-6: 2.52 (2.34, 2.72)<br>2006-7: 2.52 (2.34, 2.70)<br>2007-8: 2.51 (2.33, 2.69)<br>2008-9: 2.52 (2.34, 2.74)
Serial interval influenza B	2005-6: 2.50 (2.31,2.70)<br>2006-7: 2.48 (2.29, 2.67)<br>2007-8: 2.61 (2.41, 2.81)<br>2008-9: 2.49 (2.29, 2.70)
Probability of immunity at the start of influenza season	
< 2 y	A/H1N1 2005-9: 0.21 (0.10, 0.35)<br>A/H3N2 2005-9: 0.21 (0.10, 0.35)<br>B 2005-9: 0.21 (0.10, 0.35)
2-5 y	A/H1N1 2005-9: 0.28 (0.13, 0.51)<br>A/H3N2 2005-9: 0.25 (0.13, 0.41)<br>B 2005-9: 0.26 (0.13, 0.42)
6-11 y	A/H1N1 2005-9: 0.33 (0.29, 0.38)<br>A/H3N2 2005-9: 0.33 (0.29, 0.38)<br>B 2005-9: 0.33 (0.29, 0.38)
12-17 y	A/H1N1 2005-9: 0.33 (0.29, 0.38)<br>A/H3N2 2005-9: 0.33 (0.29, 0.38)<br>B 2005-9: 0.33 (0.29, 0.38)
18-59 y	A/H1N1 2005-9: 0.48 (0.27 0.87)<br>A/H3N2 2005-9: 0.38 (0.26, 0.50)<br>B 2005-9: 0.39 (0.23, 0.58)

Parameter	Posterior<br>mean (95% CrI)
≥60 y	A/H1N1 2005-9: 0.40 (0.20, 0.67)<br>A/H3N2 2005-9: 0.37 (0.19, 0.58)<br>B 2005-9: 0.38 (0.20, 0.61)
Proportion initially infected	Influenza A/H1N1<br>2005-6: 0.001 (4×10^{-7}, 0.007)<br>2006-7: 0.0001 (5×10^{-5}, 2×10^{-4})<br>2007-8: 9×10^{-7} (4×10^{-9}, 4×10^{-6})<br>2008-9: 8×10^{-5} (5×10^{-5}, 1×10^{-4})<br><br>Influenza A/H3N2<br>2005-6: 9×10^{-4} (4×10^{-4}, 0.001)<br>2006-7:1×10^{-6} (5×10^{-8}, 3×10^{-6})<br>2007-8: 5×10^{-5} (3×10^{-5}, 8×10^{-5})<br>2008-9: 2×10^{-4} (2×10^{-4}, 3×10^{-4})<br><br>Influenza B<br>2005-6: 6×10^{-5} (2×10^{-6}, 4×10^{-5})<br>2006-7: 2×10^{-5} (5×10^{-8}, 3×10^{-6})<br>2007-8 2×10^{-6} (1×10^{-6}, 4×10^{-6}):<br>2008-9: 1×10^{-4} (7×10^{-5}, 2×10^{-4})
Sensitivity of laboratory confirmation test	0.33 (0.24, 0.46)
Probability of symptoms given infection	0.66 (0.58, 0.75)

S12 Table. Model outcomes showing mean (95% CrI) under sensitivity analysis 2.

Total cost (million int $)	Symptoms	Outpatient visits	In-patient visits	Deaths	Incremental cost (million int $)	DALYs averted (1000s)	ICER
	Outcomes (1000 cases)				Comparison with no childhood vaccination		
No childhood vaccination							
19 (16,24)	7173 (3392, 11654)	5.3 (0.0,7.3)	3.3 (1.2,8.0)	4.3 (0.6,9.1)			
Policy 1: Vaccinate those aged 2-11 years with TIV							
237 (214, 287)	2808 (405, 8422)	2.7 (0.0,7.3)	1.0 (0.4,4.8)	1.8 (0.1, 5.6)	230 (205, 280)	47 (0, 111)	4926
Policy 2: Vaccinate those aged 2-11 years with LAIV							
296 (273, 345)	1807 (247, 4109)	2.0 (0.0,4.7)	0.5 (0.2, 0.8)	1.3 (0.0, 3.2)	288 (264, 338)	57 (11, 111)	5015
Policy 3: Vaccinate those aged 2-17 years with LAIV							
392 (359, 452)	1543 (182, 3773)	1.7 (0.0,4.5)	0.4 (0.2, 0.8)	1.1 (0.0, 2.9)	385 (351, 446)	61 (12, 116)	6343
Policy 4: Vaccinate those aged 2-5 years with LAIV							
185 (175, 196)	2286 (547, 4543)	2.3 (0.0,5.1)	0.8 (0.4, 1.4)	1.6 (0.2, 3.5)	178 (166, 190)	52 (9, 101)	3436
Policy 5: Vaccinate those aged 6-11 years with LAIV							
126 (108, 175)	3327 (1265, 5855)	2.9 (0.0,5.6)	1.2 (0.6, 2.5)	2.0 (0.1, 4.6)	119 (98, 168)	43 (6, 86)	2796
Policy 6: Vaccinate those aged 12-17 years with LAIV							
111 (95, 153)	2916 (1115, 5363)	2.8 (0.0,5.5)	1.0 (0.5, 1.9)	1.8 (0.1, 4.2)	104 (85, 146)	46 (7, 93)	2239
Policy 7: Increase vaccination coverage in those aged 60 years and over with TIV							
85 (76, 95)	5784 (2555, 9762)	4.4 (0, 7.3)	2.6 (1.0, 7.5)	3.0 (0.6, 6.7)	78 (70, 87)	19 (0,46)	4172

S16 Figure. Model fits to laboratory-confirmed influenza surveillance data under sensitivity analysis 2 . Monthly numbers of PCR-confirmed influenza cases (circles) and model predictions: median (broken line), and 95%, 90% and 80% (gray shading) prediction intervals for the expected number of cases.

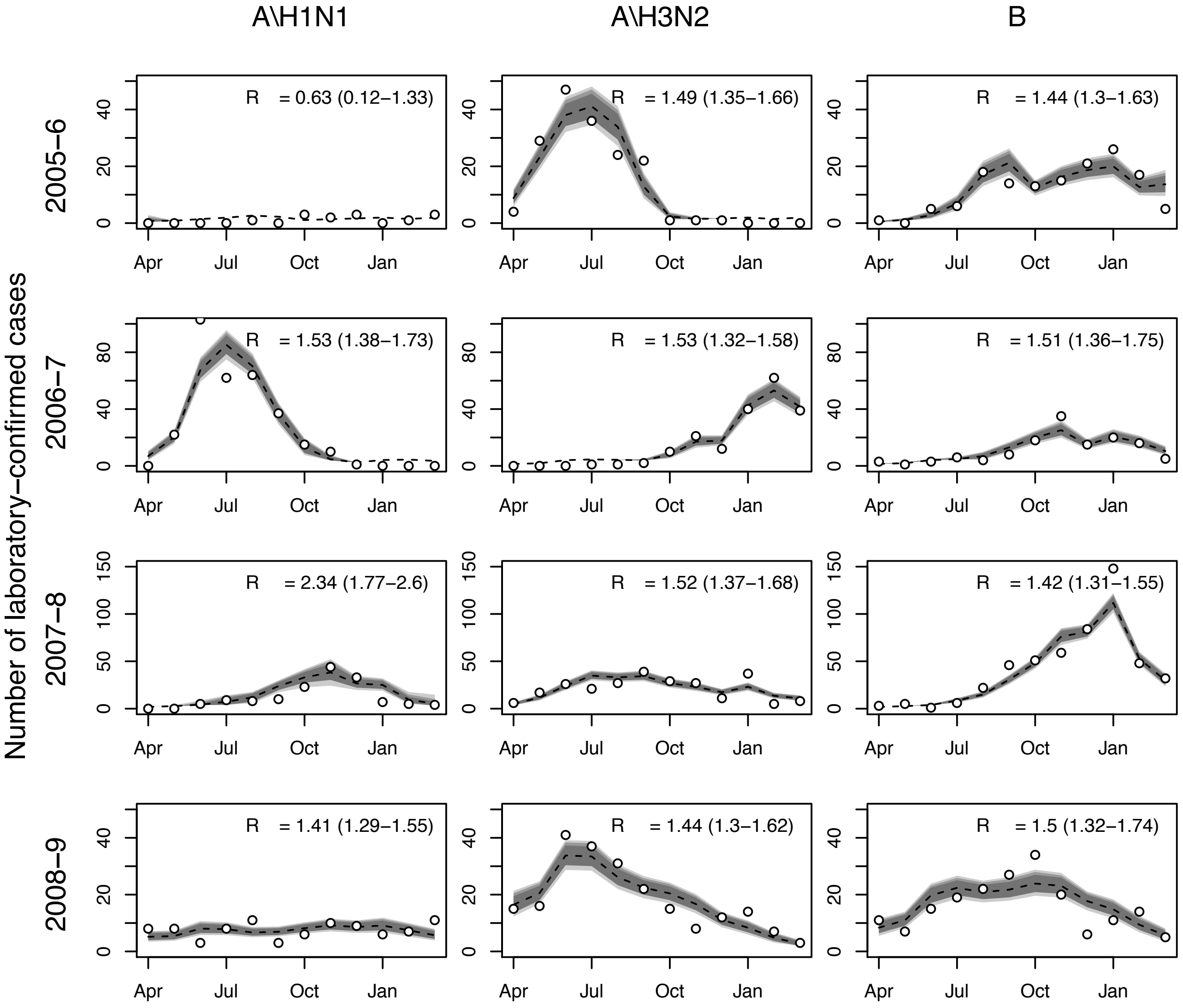

S17 Figure. Cost-effective Acceptability Curves under sensitivity analysis 2.

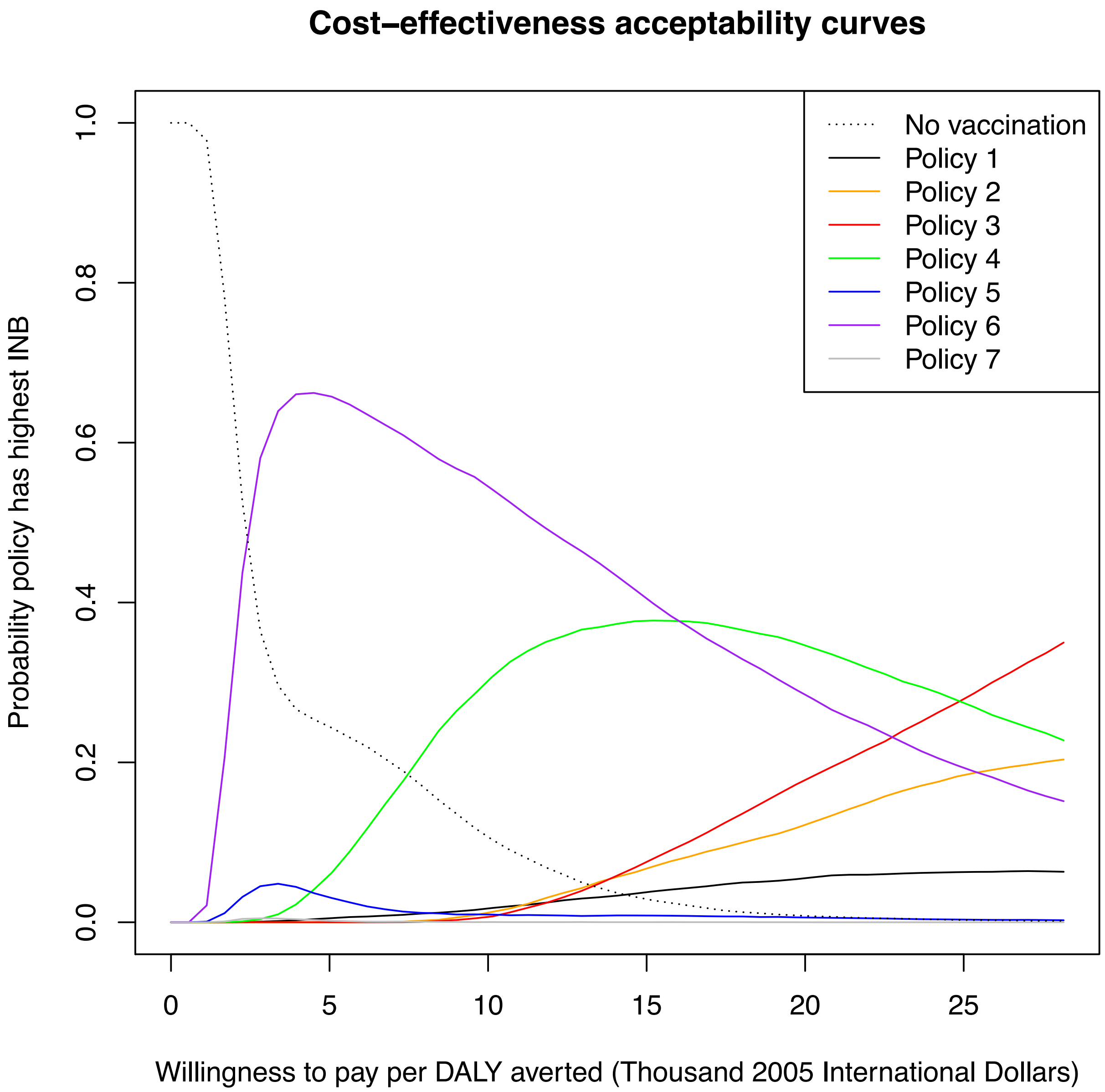

S18 Figure. Estimated numbers of symptomatic cases of seasonal influenza infection under sensitivity analysis 2.

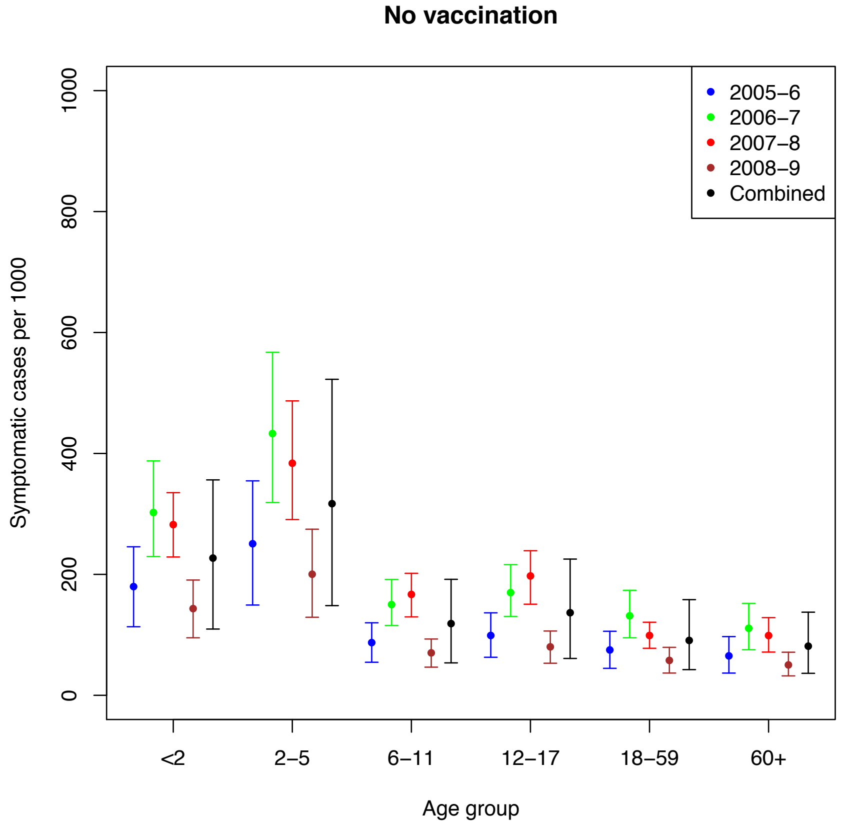

S19 Figure. Symptomatic cases averted under sensitivity analysis 2.

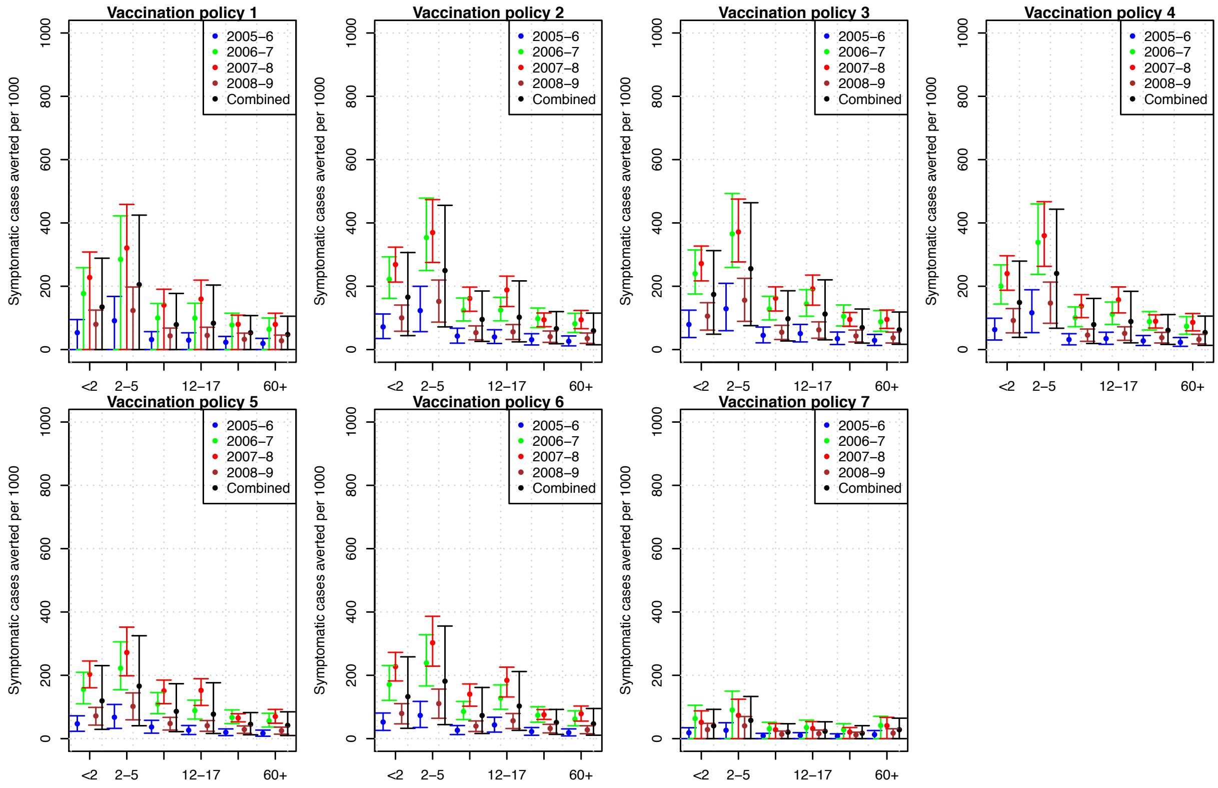

S20 Figure. Estimated number of deaths by age group due to influenza in the absence of vaccination against seasonal influenza under sensitivity analysis 2.

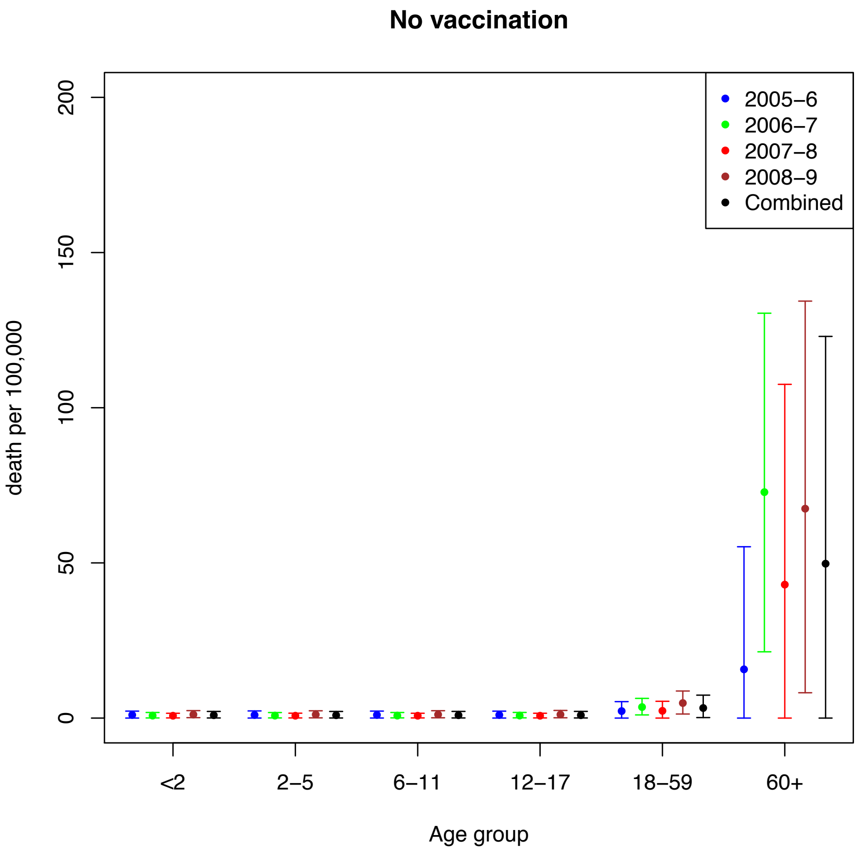

S21 Figure. Deaths averted under sensitivity analysis 2.

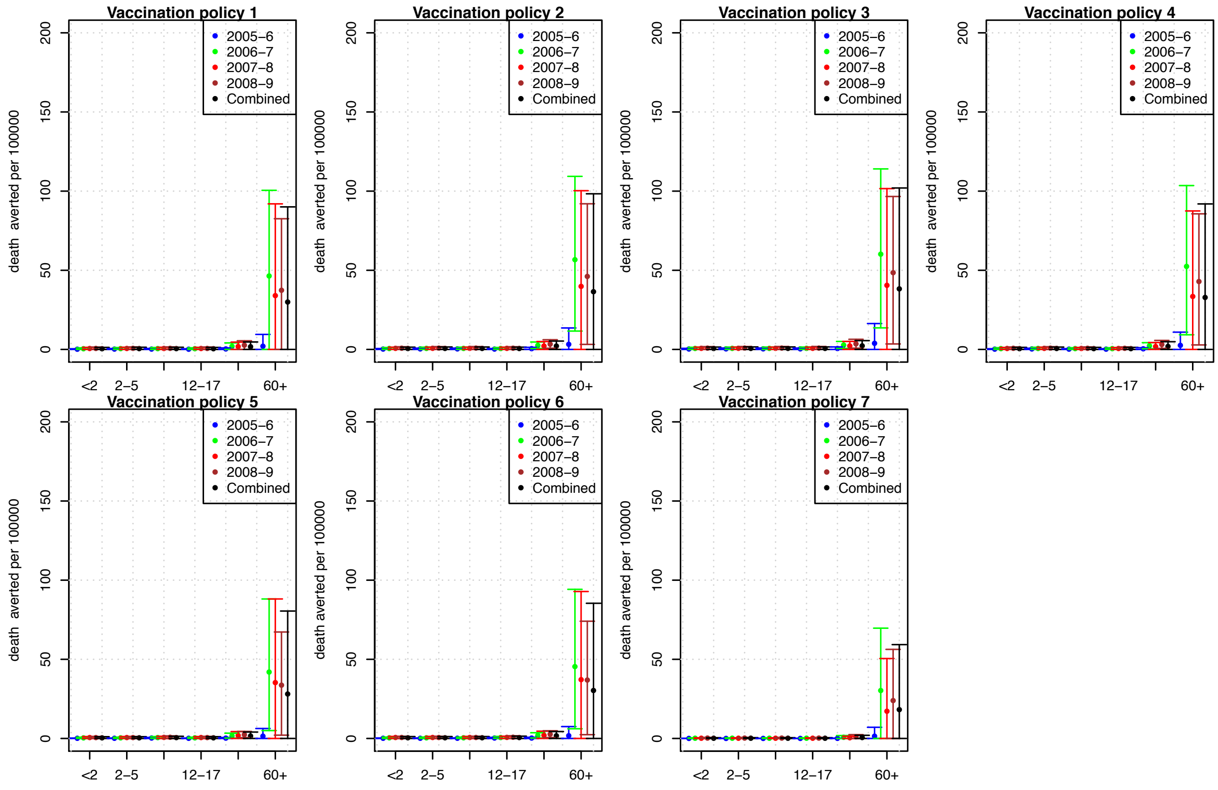

S22 Figure. DALYs averted as a result of direct and indirect vaccine effects under sensitivity analysis 2.

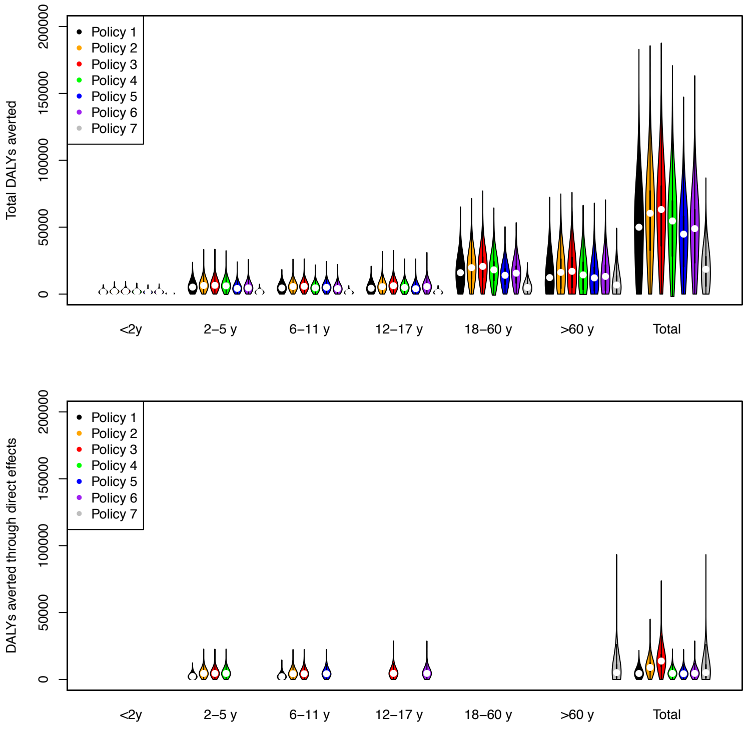

S23 Figure. Expected value of perfect information under sensitivity analysis 2.

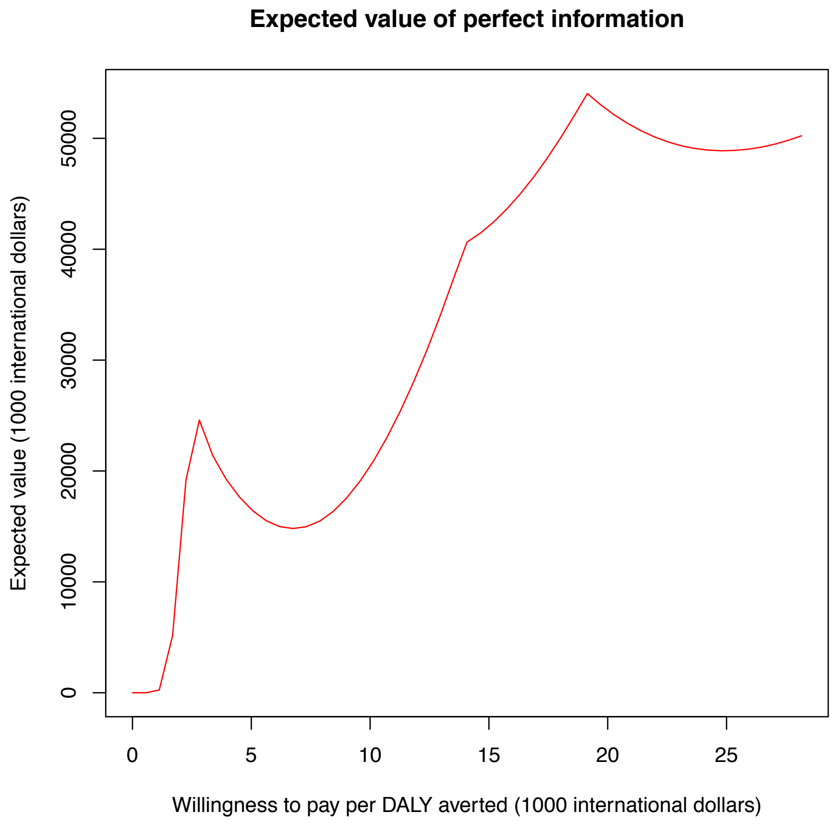

3.3 Additional results from sensitivity analysis 3

Note that in this sensitivity analysis only the vaccine assumptions change from the base case, so all model results which are not affected by the vaccine are the same as those in the base case model.

S13 Table. Model outcomes showing mean (95% CrI) under sensitivity analysis 3.

Total cost (million int $)	Outcomes (1000 cases)				Comparison with no childhood vaccination		
	Symptoms	Outpatient visits	In-patient visits	Deaths	Incremental cost (million int $)	DALYs averted (1000s)	ICER
No childhood vaccination							
20 (16,23)	8576 (4445, 14427)	5.3 (0.0,7.3)	3.3 (1.2,8.0)	4.3 (0.6,9.2)			
Policy 1: Vaccinate those aged 2-11 years with TIV							
237 (214, 287)	3484 (584, 9073)	2.7 (0.0, 7.3)	0.9 (0.4, 2.5)	1.8 (0.2, 5.3)	218 (193, 268)	50 (0, 106)	4366
Policy 2: Vaccinate those aged 2-11 years with LAIV							
296 (273, 346)	2641 (504, 5447)	2.2 (0.0,4.9)	0.6 (0.4, 0.9)	1.5 (0.2, 3.5)	276 (252, 327)	58 (12, 114)	4747
Policy 3: Vaccinate those aged 2-17 years with LAIV							
392 (359, 453)	2226 (382, 4967)	1.9 (0.0,4.6)	0.5 (0.3, 0.8)	1.2 (0.1, 3.1)	372 (339, 434)	63 (14, 122)	5952
Policy 4: Vaccinate those aged 2-5 years with LAIV							
185 (175, 196)	3232 (830, 6135)	2.6 (0.0,5.3)	0.8 (0.5, 1.2)	1.7 (0.2, 3.9)	166 (154, 178)	52 (9, 104)	3168
Policy 5: Vaccinate those aged 6-11 years with LAIV							
127 (109, 176)	4627 (2242, 7961)	3.2 (0.0,5.8)	1.4 (0.7, 3.1)	2.3 (0.4, 5.1)	107 (87, 156)	40 (7, 80)	2691
Policy 6: Vaccinate those aged 12-17 years with LAIV							
112 (96, 153)	4022 (1595, 7131)	3.0 (0.0,5.6)	1.2 (0.6, 2.3)	2.0 (0.3, 4.6)	92 (74, 134)	45 (8, 90)	2028
Policy 7: Increase vaccination coverage in those aged 60 years and over with TIV							
85 (76, 94)	6606 (3289, 11666)	4.3 (0.9, 7.3)	2.4 (1.0, 6.7)	2.8 (0.5, 6.6)	65 (57, 74)	23 (0, 54)	2844

S24 Figure. Cost-effective Acceptability Curves under sensitivity analysis 3.

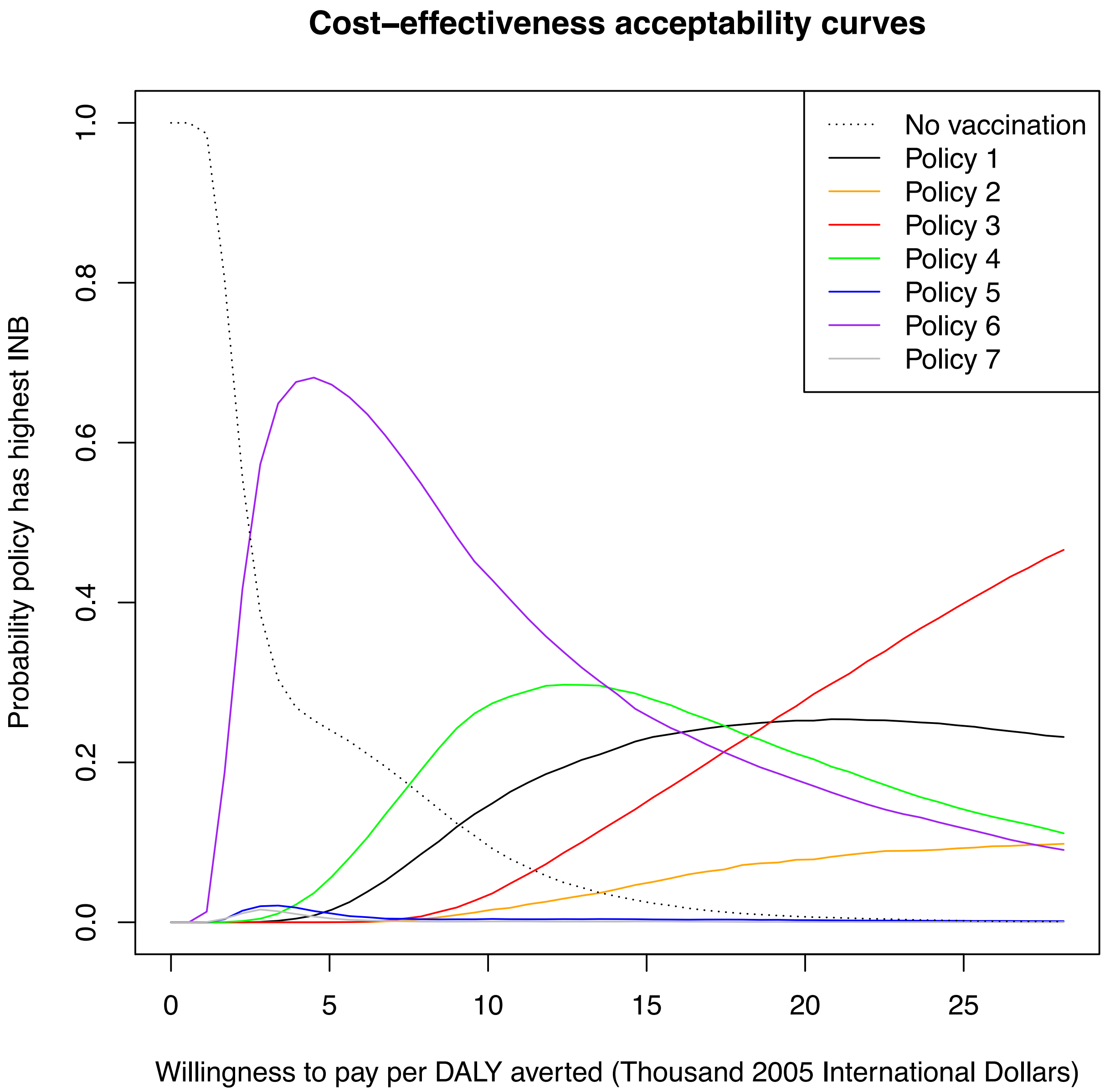

S25 Figure. Estimated numbers of symptomatic cases of seasonal influenza infection under sensitivity analysis 3.

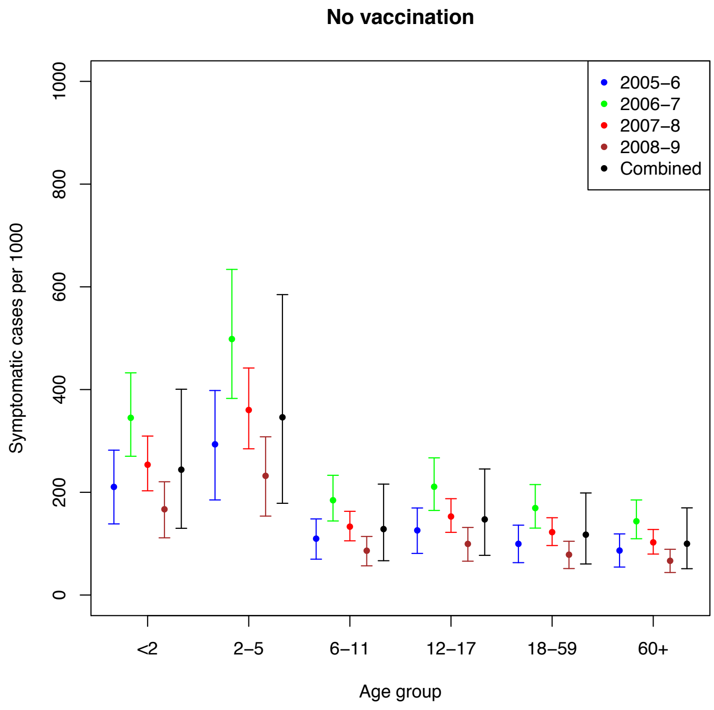

S26 Figure. Symptomatic cases averted under sensitivity analysis 3.

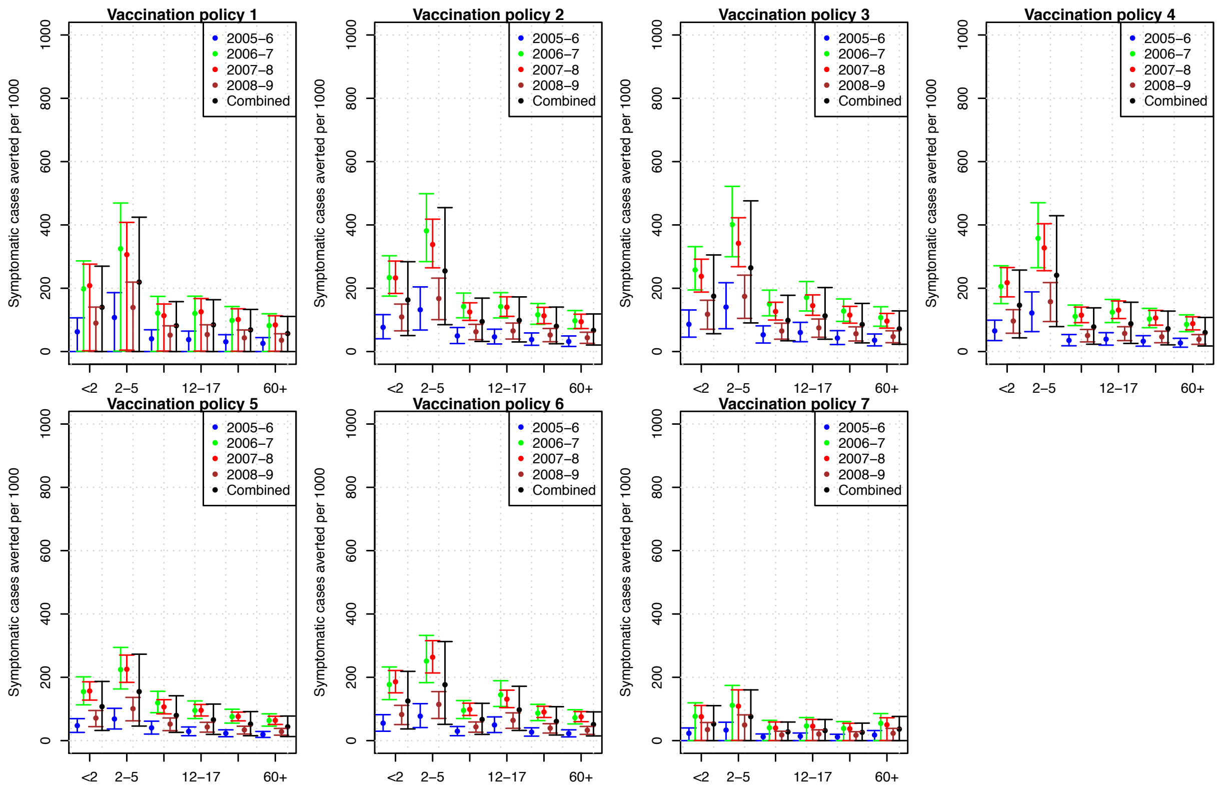

S27 Figure. DALYs averted as a result of direct and indirect vaccine effects under sensitivity analysis 3.

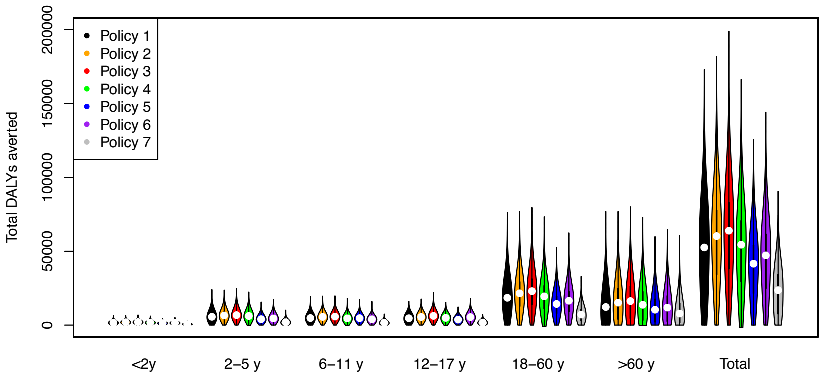

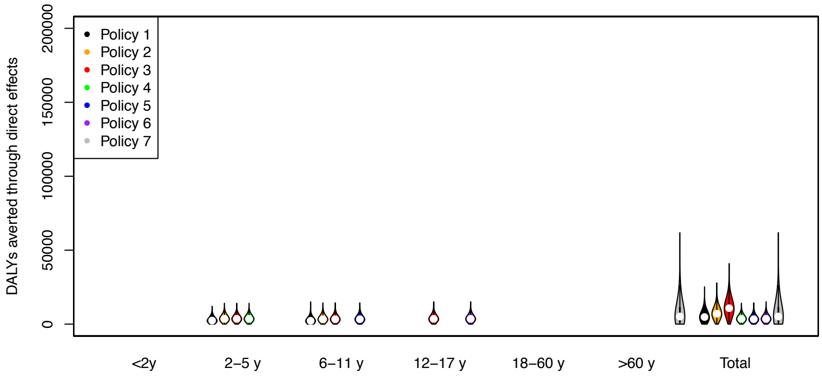

S28 Figure. Expected value of perfect information under sensitivity analysis 3.

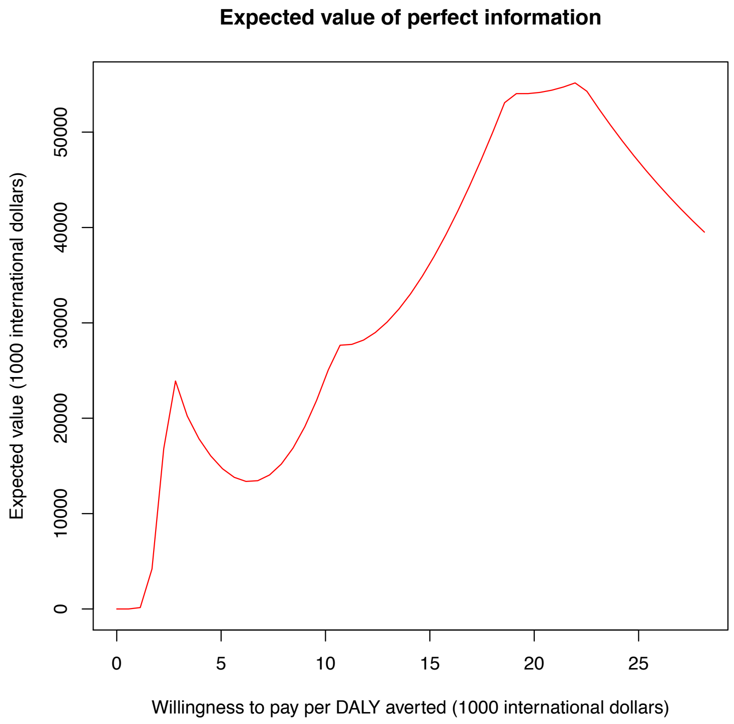

3.4 Additional results from sensitivity analysis 4

Note that in this sensitivity analysis only the vaccine assumptions change from the base case, so all model results which are not affected by the vaccine are the same as those in the base case model.

S14 Table. Model outcomes showing mean (95% CrI) under sensitivity analysis 4.

	Outcomes (1000 cases)				Comparison with no childhood vaccination		
Total cost (million int $)	Symptomatic infections	Outpatient visits	In-patient visits	Deaths	Incremental cost (million int $)	DALYs averted (1000s)	ICER
No childhood vaccination							
20 (16,24)	8563 (4445, 14331)	5.3 (0.0,7.3)	3.3 (1.2,8.0)	4.3 (0.6, 9.2)			
Policy 1: Vaccinate those aged 2-11 years with TIV							
197 (177, 238)	3984 (784,10204)	3.0 (0.0,7.3)	1.1 (0.5,5.1)	2.1 (0.2, 6.0)	177 (157, 219)	45 (0, 99)	3976
Policy 2: Vaccinate those aged 2-11 years with LAIV							
244 (225, 285)	2653 (499, 5462)	2.2 (0.0, 4.9)	0.6 (0.4, 0.9)	1.5 (0.1, 3.6)	224 (204, 266)	58 (11, 113)	3880
Policy 3: Vaccinate those aged 2-17 years with LAIV							
317 (291, 365)	2246 (381, 5010)	1.9 (0.0,4.7)	0.5 (0.3, 0.8)	1.3 (0.1, 3.2)	297 (270, 344)	62 (13, 121)	4790
Policy 4: Vaccinate those aged 2-5 years with LAIV							
154 (154, 163)	3227 (789, 6114)	2.6 (0.0,5.3)	0.8 (0.5, 1.1)	1.7 (0.2, 4.0)	134 (124, 144)	52 (9, 104)	2586
Policy 5: Vaccinate those aged 6-11 years with LAIV							
106 (91, 157)	4588 (2209, 7828)	3.2 (0.0,5.8)	1.4 (0.7, 3.0)	2.3 (0.3, 5.2)	86 (69, 128)	40 (6, 81)	2164
Policy 6: Vaccinate those aged 12-17 years with LAIV							
89 (76, 122)	3997 (1548, 7096)	3.0 (0.0,5.6)	1.2 (0.6, 2.2)	2.1 (0.2, 4.7)	69 (54, 103)	45 (7, 91)	1524
Policy 7: Increase vaccination coverage in those aged 60 years and over with TIV							
85 (76, 95)	6648 (3312, 11850)	4.3 (0, 7.3)	2.4 (1.0, 7.2)	2.9 (0.5, 6.9)	65 (57, 75)	22 (0, 54)	2952

S29 Figure. Cost-effective Acceptability Curves under sensitivity analysis 4.

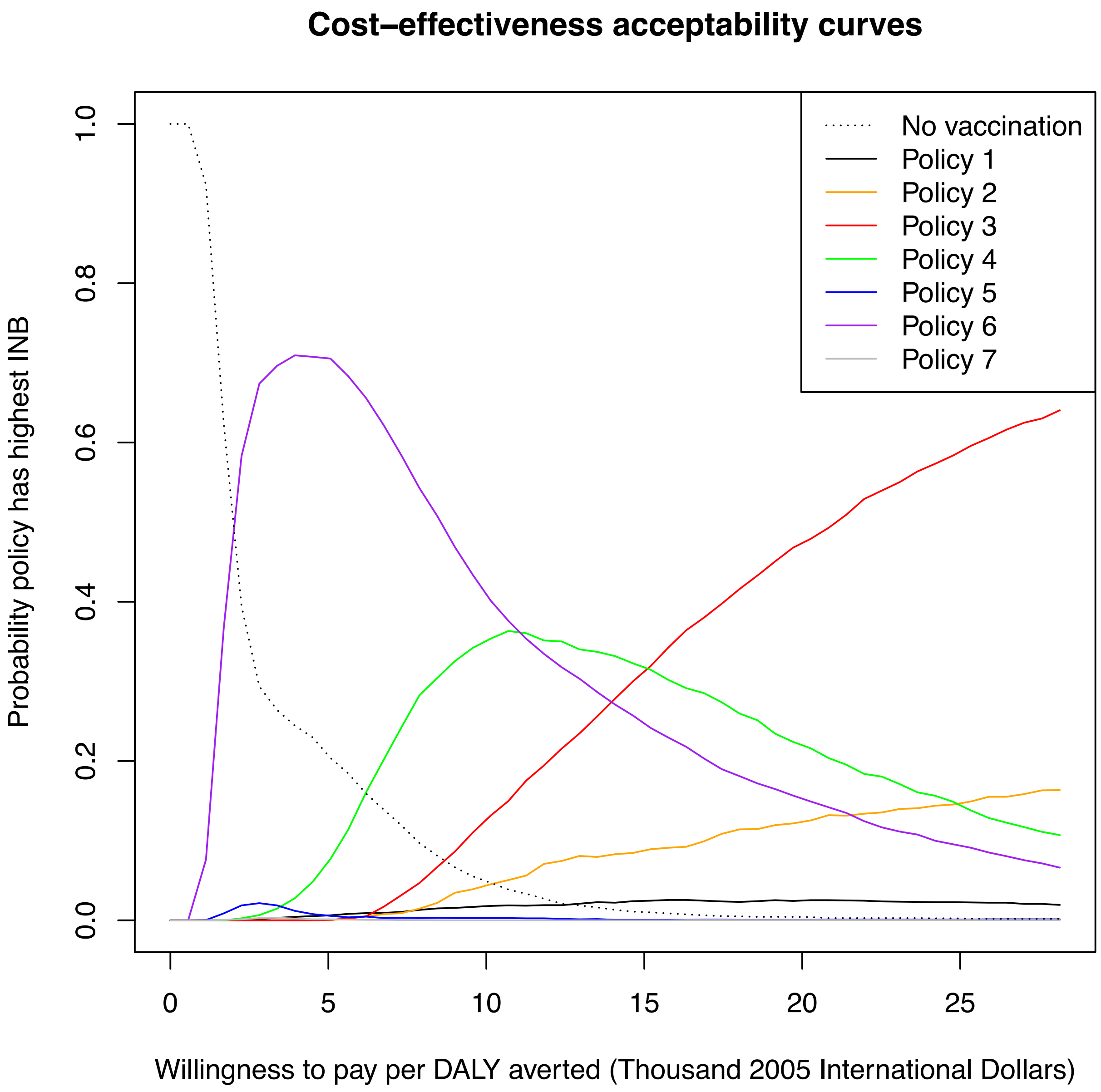

S30 Figure. Symptomatic cases averted under sensitivity analysis 4.

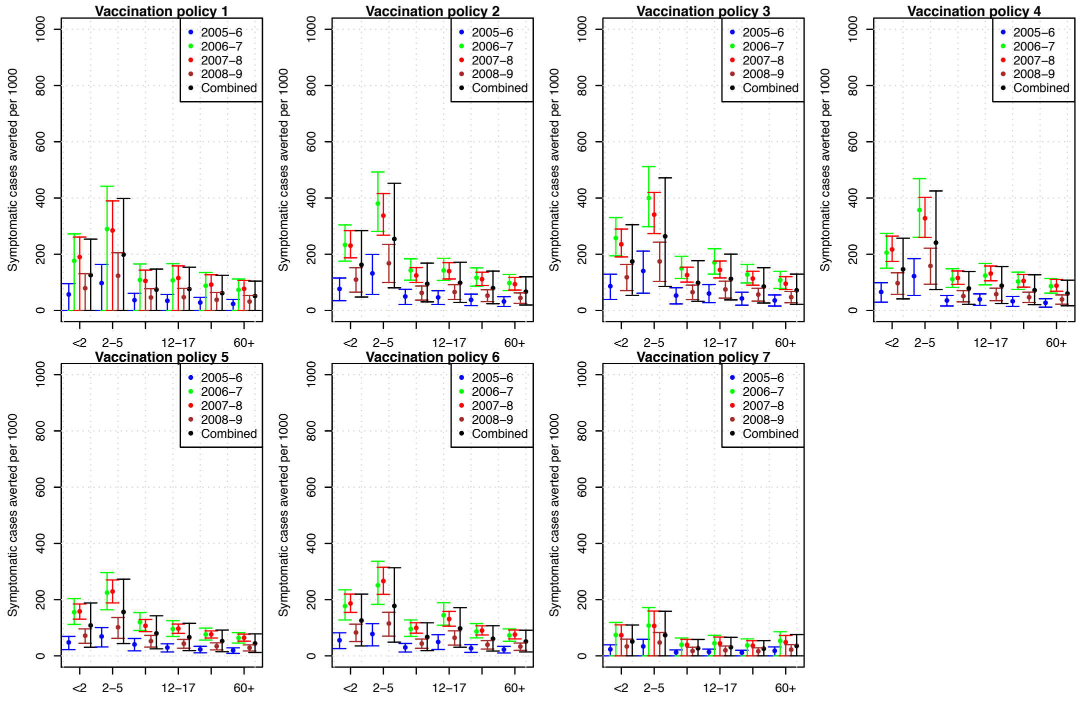

S31 Figure. Deaths averted under sensitivity analysis 4.

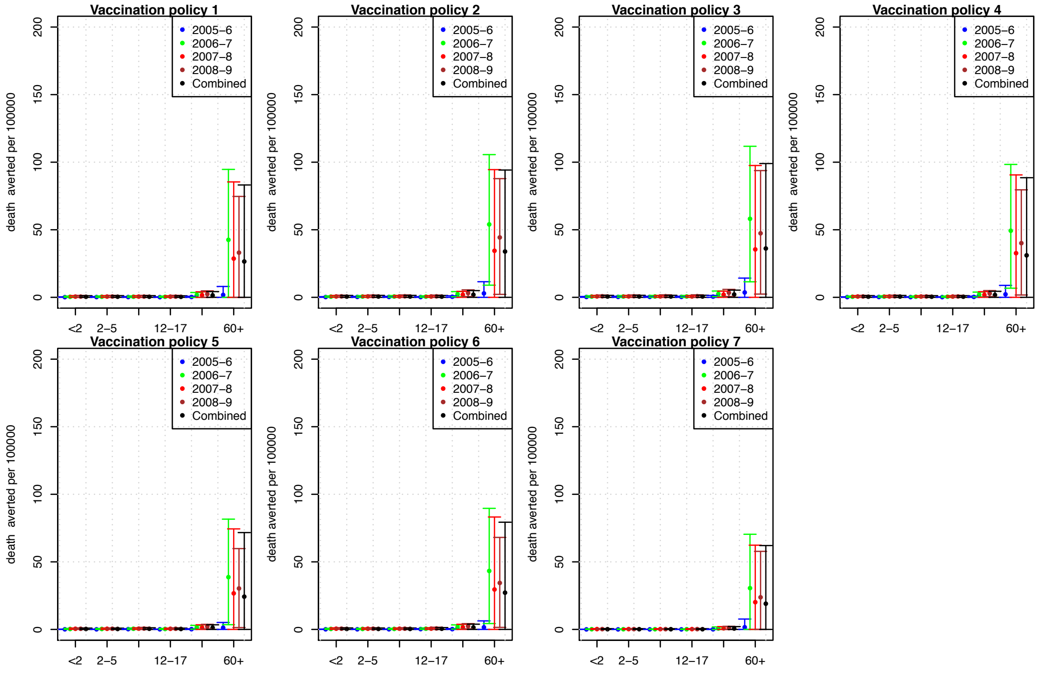

S32 Figure. DALYs averted as a result of direct and indirect vaccine effects under sensitivity analysis 4.

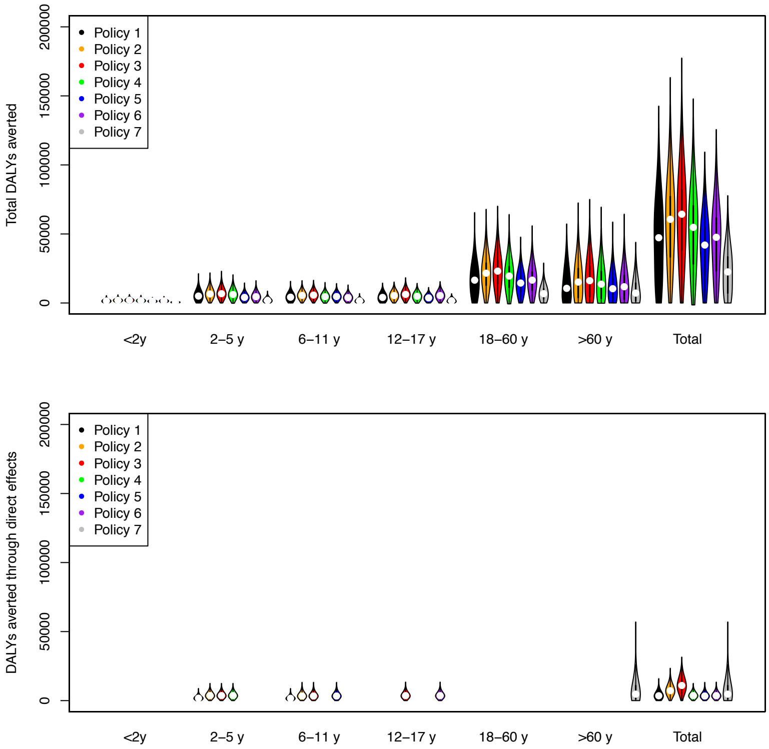

S33 Figure. Expected value of perfect information under sensitivity analysis 4.

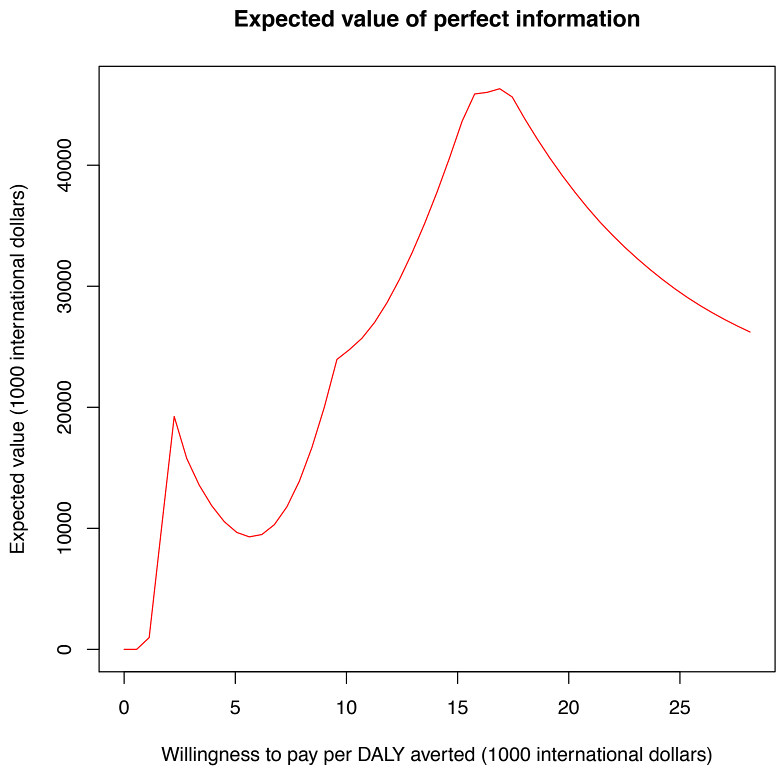

3.5 Additional results from sensitivity analysis 5

S15 Table. Posterior distributions from sensitivity analysis 5

Parameter	Posterior mean (95% CrI)
R_0 for influenza A/H1N1	2005-6: 1.11 (1.01, 1.19)<br>2006-7: 1.25 (1.19, 1.31)<br>2007-8: 1.18 (1.12, 1.25)<br>2008-9: 1.14 (1.09 1.20)
R_0 for influenza A/H3N2	2005-6: 1.22 (1.15, 1.29)<br>2006-7: 1.20 (1.15, 1.27)<br>2007-8: 1.17 (1.12 1.23)<br>2008-9: 1.16 (1.11, 1.21)
R_0 for influenza B	2005-6: 1.17 (1.12, 1.23)<br>2006-7: 1.18 (1.13, 1.25)<br>2007-8: 1.20 (1.15, 1.26)<br>2008-9: 1.17 (1.12, 1.23)
Serial interval influenza A/H1N1	2005-6: 2.50 (2.31, 2.70)<br>2006-7: 2.54 (2.35, 2.75)<br>2007-8: 2.40 (2.21, 2.60)<br>2008-9: 2.52 (2.32 2.72)
Serial interval influenza A/H3N2	2005-6: 2.52 (2.33, 2.72)<br>2006-7: 2.52 (2.33, 2.73)<br>2007-8: 2.51 (2.32, 2.71)<br>2008-9: 2.53 (2.34, 2.74)
Serial interval influenza B	2005-6: 2.51 (2.32, 2.71)<br>2006-7: 2.48 (2.29, 2.67)<br>2007-8: 2.64 (2.43, 2.86)<br>2008-9: 2.48 (2.30, 2.68)
Probability of immunity at the start of influenza season	
< 2 y	A/H1N1 2005-9: 0.11 (0.05, 0.18)<br>A/H3N2 2005-9: 0.11 (0.05, 0.18)<br>B 2005-9: 0.11 (0.05, 0.18)
2-5 y	A/H1N1 2005-9: 0.14 (0.07, 0.22)<br>A/H3N2 2005-9: 0.13 (0.07, 0.22)<br>B 2005-9: 0.13 (0.07, 0.22)
6-11 y	A/H1N1 2005-9: 0.17 (0.14, 0.19)<br>A/H3N2 2005-9: 0.17 (0.14, 0.19)<br>B 2005-9: 0.17 (0.14, 0.19)
12-17 y	A/H1N1 2005-9: 0.17 (0.14, 0.19)<br>A/H3N2 2005-9: 0.17 (0.14, 0.19)<br>B 2005-9: 0.17 (0.14, 0.19)
18-59 y	A/H1N1 2005-9: 0.21 (0.15, 0.29)<br>A/H3N2 2005-9: 0.21 (0.14, 0.28)<br>B 2005-9: 0.21 (0.14, 0.29)

Parameter	Posterior mean (95% CrI)
≥60 y	A/H1N1 2005-9: 0.19 (0.10, 0.30)<br>A/H3N2 2005-9: 0.19 (0.10, 0.30)<br>B 2005-9: 0.19 (0.10, 0.30)
Proportion initially infected	Influenza A/H1N1<br>2005-6: 1×10^{-4} (1×10^{-8}, 3×10^{-4})<br>2006-7: 1×10^{-4} (6×10^{-5}, 2×10^{-4}),<br>2007-8: 3×10^{-5} (2×10^{-5}, 4×10^{-5}),<br>2008-9: 1×10^{-4} (6×10^{-5}, 1×10^{-4}),<br><br>Influenza A/H3N2<br>2005-6: 0.001 (4×10^{-4}, 0.001)<br>2006-7: 1×10^{-6} (4×10^{-8}, 3×10^{-6})<br>2007-8: 8×10^{-5} (5×10^{-5}, 1×10^{-4})<br>2008-9: 3×10^{-4} (2×10^{-4}, 4×10^{-4})<br><br>Influenza B<br>2005-6: 6×10^{-5} (3×10^{-5}, 1×10^{-4}),<br>2006-7: 2×10^{-5} (7×10^{-6}, 5×10^{-5}),<br>2007-8: 3×10^{-6} (2×10^{-6}, 5×10^{-6}),<br>2008-9: 1×10^{-4} (8×10^{-5}, 2×10^{-4}),
Sensitivity of laboratory confirmation test	0.34 (0.25, 0.44)
Probability of symptoms given infection	0.68 (0.59, 0.76)

S16 Table. Model outcomes showing mean (95% CrI) under sensitivity analysis 5.

Total cost (million int $)	Outcomes (1000s)				Comparison with no childhood vaccination		
	Symptomatic infections	Outpatient visits	In-patient visits	Deaths	Incremental cost (million int $)	DALYs averted (1000s)	ICER
No childhood vaccination							
20 (16,24)	8972 (4827, 14963)	5.3 (0.0,7.3)	3.3 (1.2,8.0)	4.3 (0.6,9.2)			
Policy 1: Vaccinate those aged 2-11 years with TIV							
237 (214, 285)	3581 (575, 9514)	2.6 (0.0,7.1)	0.9 (0.4, 2.5)	1.8 (0.2, 5.3)	217 (193, 265)	52 (0, 109)	4175
Policy 2: Vaccinate those aged 2-11 years with LAIV							
295 (273, 343)	2453 (407, 5436)	1.9 (0.0,4.6)	0.5 (0.3, 0.8)	1.2 (0.1, 3.1)	276 (252, 324)	63 (16, 122)	4368
Policy 3: Vaccinate those aged 2-17 years with LAIV							
391 (359, 451)	2059 (324, 4907)	1.7 (0.0,4.2)	0.4 (0.3, 0.7)	1.1 (0.1, 2.7)	372 (338, 432)	67 (18, 130)	5536
Policy 4: Vaccinate those aged 2-5 years with LAIV							
185 (175, 196)	2988 (589, 6061)	2.3 (0.0,5.0)	0.7 (0.4, 0.9)	1.5 (0.2, 3.5)	165 (153, 177)	58 (13, 112)	2866
Policy 5: Vaccinate those aged 6-11 years with LAIV							
126 (108, 173)	4208 (1631, 7408)	2.9 (0.0,5.5)	1.1 (0.6, 2.2)	2.0 (0.3, 4.5)	106 (85, 153)	47 (10, 92)	2269
Policy 6: Vaccinate those aged 12-17 years with LAIV							
111 (95, 153)	3675 (1066, 6803)	2.7 (0.0,5.3)	0.9 (0.5, 1.5)	1.8 (0.2, 4.1)	91 (73, 133)	52 (11, 101)	1774
Policy 7: Increase vaccination coverage in those aged 60 years and over with TIV							
85 (77, 95)	6913 (3535, 1229)	4.3 (0.0, 7.3)	2.4 (1.0, 6.4)	2.8 (0.4, 6.6)	65 (57, 74)	24 (9, 55)	2745

S34 Figure. Model fits to laboratory-confirmed influenza surveillance data under sensitivity analysis 5. Monthly numbers of PCR-confirmed influenza cases (circles) and model predictions: median (broken line), and 95%, 90% and 80% (gray shading) prediction intervals for the expected number of cases.

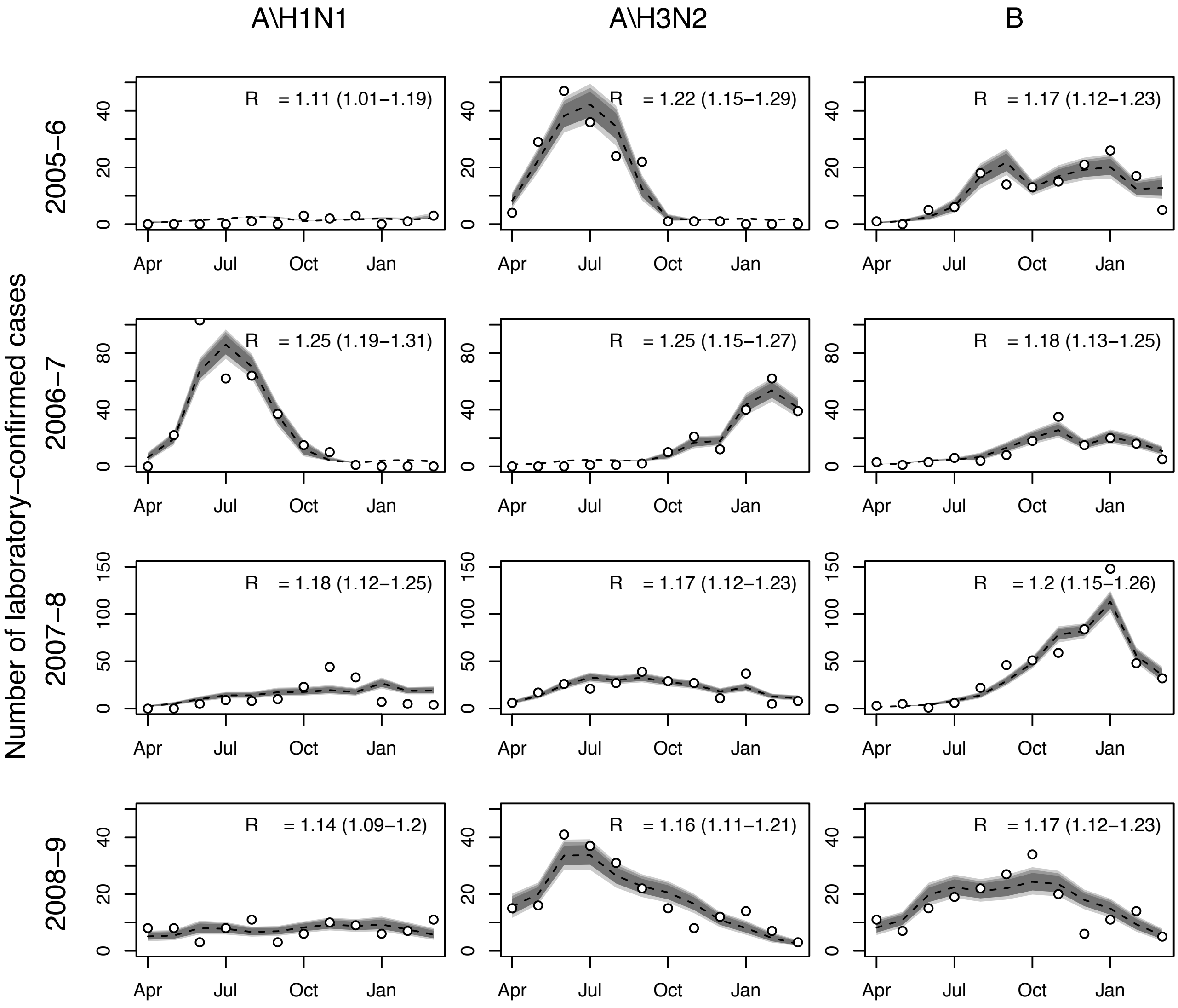

S35 Figure. Cost-effective Acceptability Curves under sensitivity analysis 5.

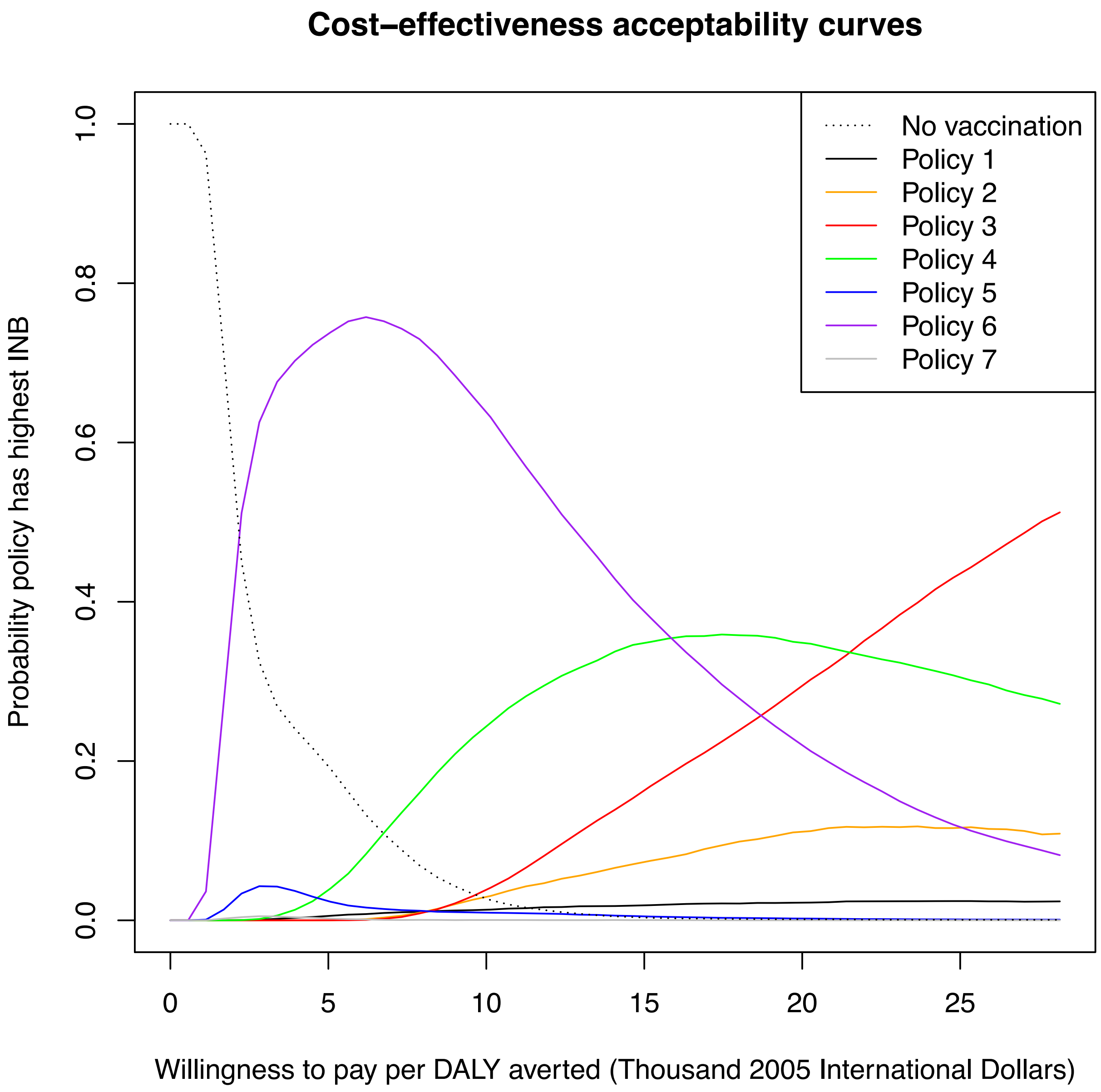

S36 Figure. Estimated numbers of symptomatic cases of seasonal influenza infection under sensitivity analysis 5.

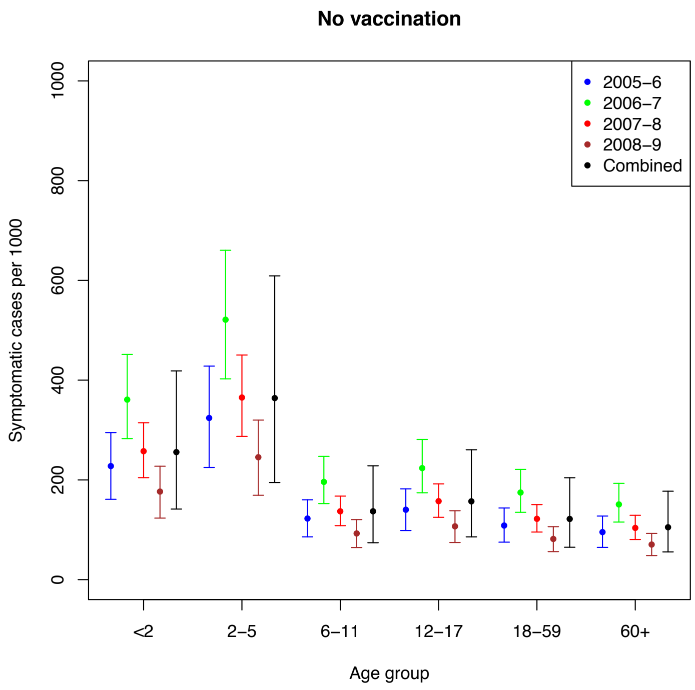

S37 Figure. Symptomatic cases averted under sensitivity analysis 5

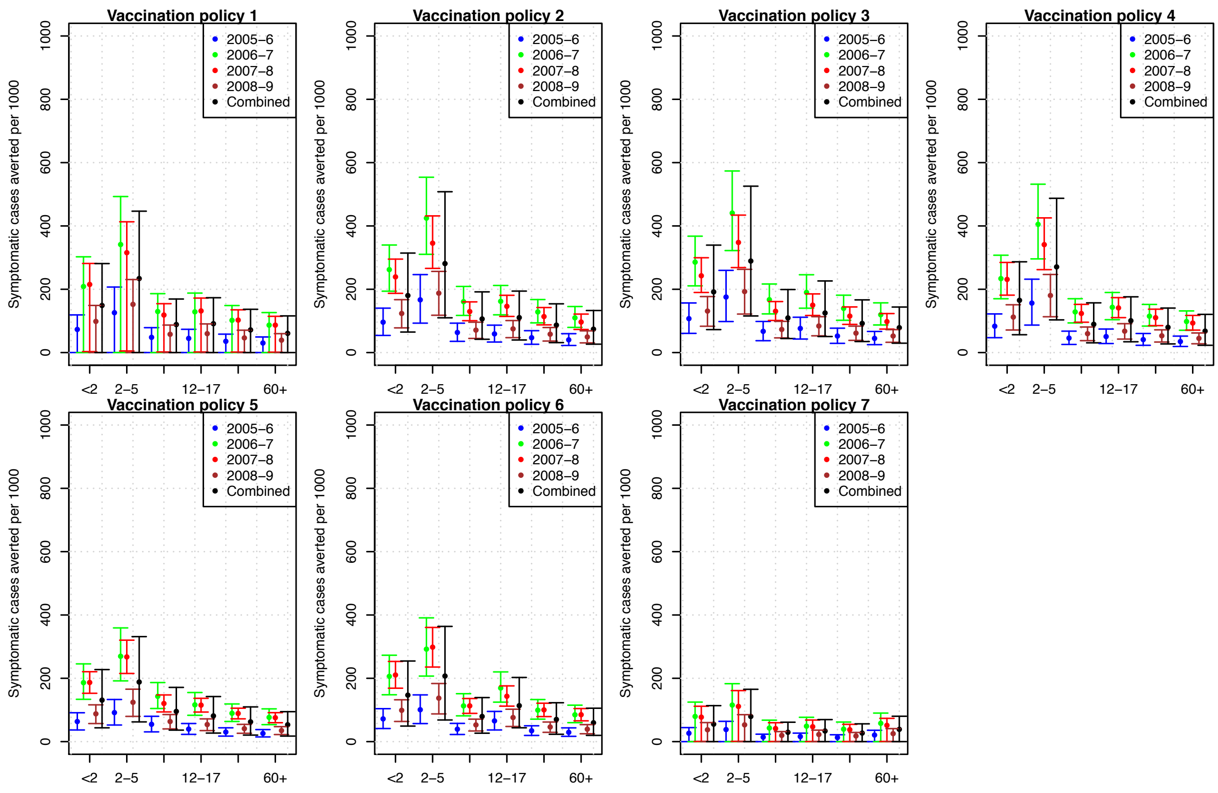

S38 Figure. Estimated number of deaths by age group due to influenza in the absence of vaccination against seasonal influenza under sensitivity analysis 5 .

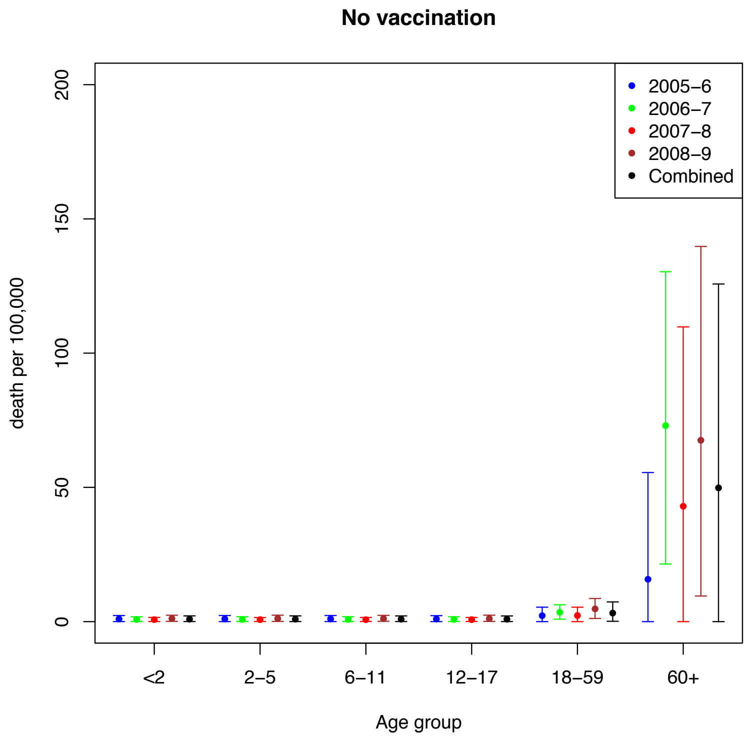

S39 Figure. Deaths averted under sensitivity analysis 5.

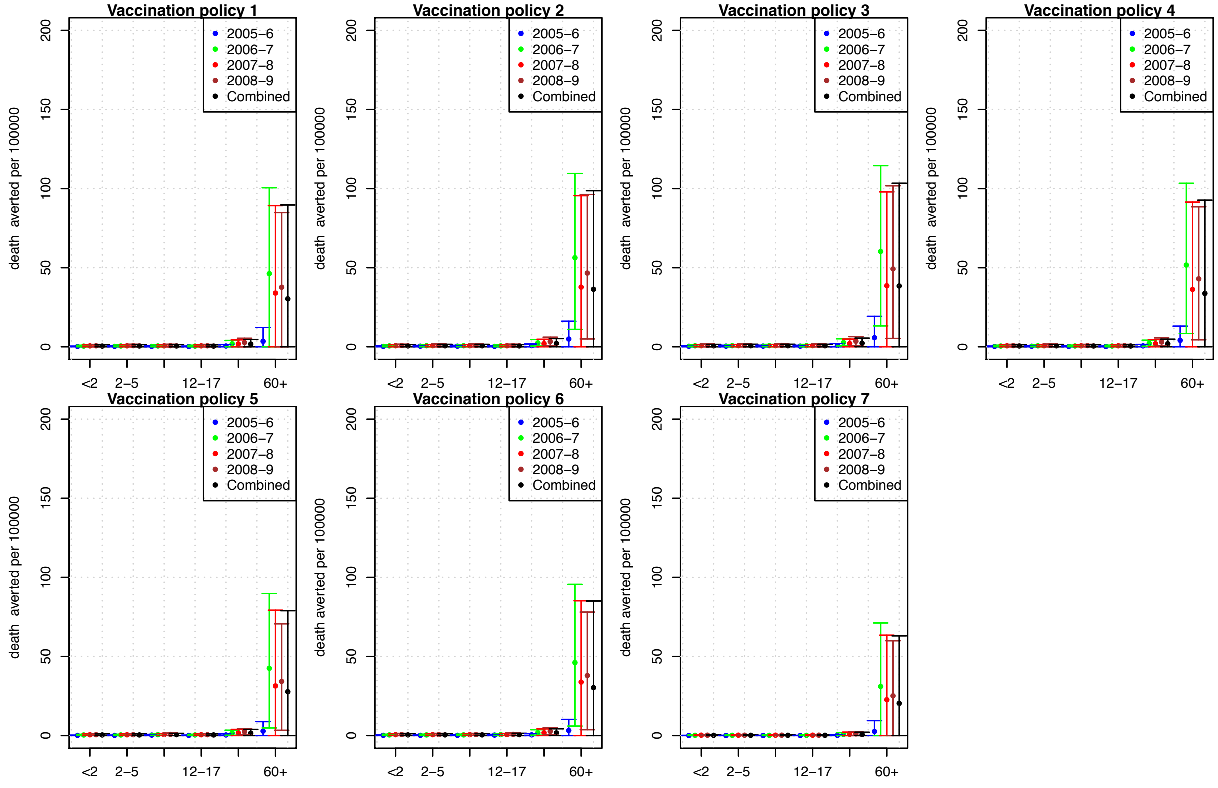

S40 Figure. DALYs averted as a result of direct and indirect vaccine effects under sensitivity analysis 5.

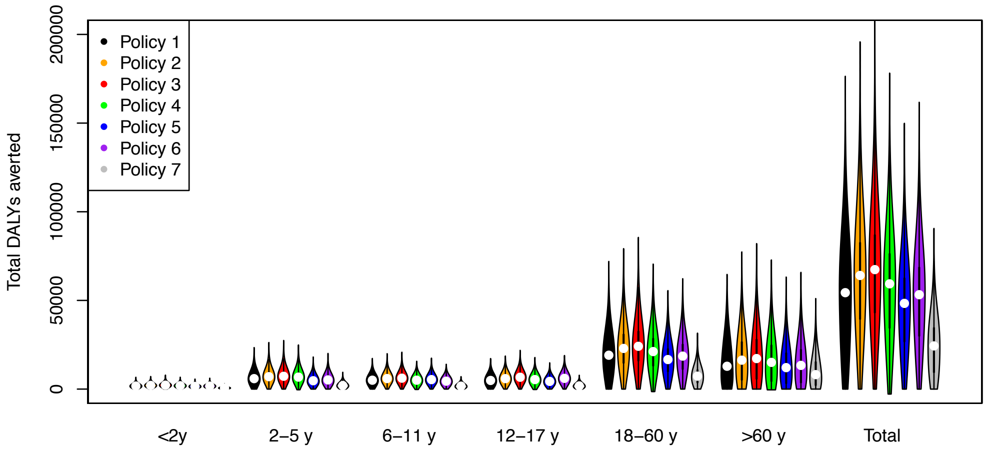

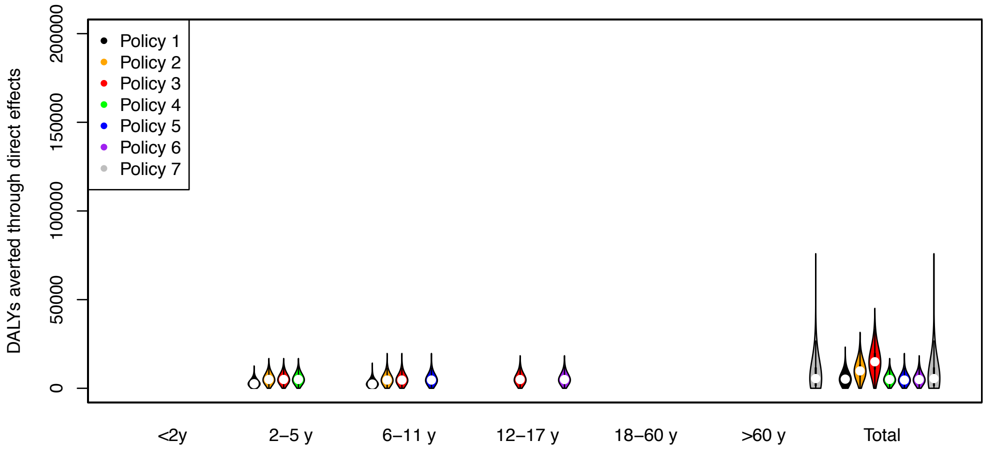

S40 Figure. Expected value of perfect information under sensitivity analysis 5.

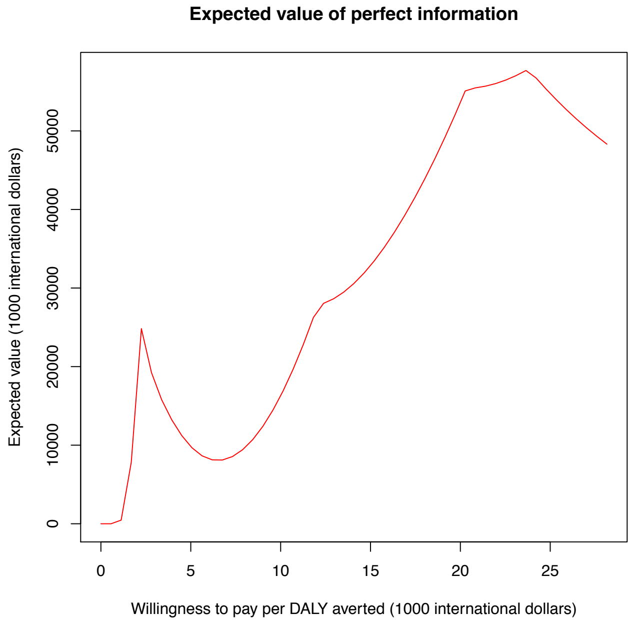

3.6 Additional results from sensitivity analysis 6

S17 Table. Posterior distributions from sensitivity analysis 6 (prior for age-specific immunity at start of each influenza season derived from Baguelin *et al.* 2013).

Parameter	Posterior mean (95% CrI)
R_0 for influenza A/ H1N1	2005-6: 0.59 (0.13, 1.24)<br>2006-7: 1.38 (1.23, 1.57)<br>2007-8: 2.27 (2.01, 2.67)<br>2008-9: 1.33 (1.19 1.47)
R_0 for influenza A/ H3N2	2005-6: 1.38 (1.20, 1.66)<br>2006-7: 1.38 (1.21, 1.61)<br>2007-8: 1.50 (1.27, 1.75)<br>2008-9: 1.28 (1.16, 1.45)
R_0 for influenza B	2005-6: 1.41 (1.15, 1.60)<br>2006-7: 1.48 (1.23, 1.65)<br>2007-8: 1.34 (1.22, 1.52)<br>2008-9: 1.37 (1.17, 1.55)
Serial interval influenza A/H1N1	2005-6: 2.50 (2.31, 2.70)<br>2006-7: 2.53 (2.34, 2.72)<br>2007-8: 2.43 (2.26, 2.63)<br>2008-9: 2.52 (2.33 2.72)
Serial interval influenza A/H3N2	2005-6: 2.51 (2.32, 2.71)<br>2006-7: 2.52 (2.33, 2.71)<br>2007-8: 2.49 (2.32, 2.68)<br>2008-9: 2.52 (2.34, 2.73)
Serial interval influenza B	2005-6: 2.50 (2.31, 2.70)<br>2006-7: 2.48 (2.29, 2.67)<br>2007-8: 2.60 (2.41, 2.81)<br>2008-9: 2.49 (2.30, 2.69)
Probability of immunity at the start of influenza season	
< 2 y	A/H1N1 2005-9: 0.34 (0.14, 0.57)<br>A/H3N2 2005-9: 0.33 (0.15, 0.56)<br>B 2005-9: 0.34 (0.15, 0.56)
2-5 y	A/H1N1 2005-9: 0.39 (0.15, 0.70)<br>A/H3N2 2005-9: 0.34 (0.15, 0.55)<br>B 2005-9: 0.34 (0.15, 0.57)
6-11 y	A/H1N1 2005-9: 0.38 (0.14, 0.69)<br>A/H3N2 2005-9: 0.33 (0.13, 0.55)<br>B 2005-9: 0.33 (0.13, 0.56)
12-17 y	A/H1N1 2005-9: 0.29 (0.09, 0.55)<br>A/H3N2 2005-9: 0.33 (0.14, 0.55)<br>B 2005-9: 0.32 (0.14, 0.56)
18-59 y	A/H1N1 2005-9: 0.38 (0.09, 0.87)<br>A/H3N2 2005-9: 0.26 (0.08, 0.50)<br>B 2005-9: 0.28 (0.07, 0.45)

Parameter	Posterior mean (95% CrI)
≥60 y	A/H1N1 2005-9: 0.40 (0.06, 0.81)<br>A/H3N2 2005-9: 0.33 (0.05, 0.72)<br>B 2005-9: 0.35 (0.06, 0.74)
Proportion initially infected	Influenza A/H1N1<br>2005-6: 0.001 (9×10^{-7}, 0.007)<br>2006-7: 1×10^{-4} (6×10^{-5}, 2×10^{-4}),<br>2007-8: 3×10^{-7} (4×10^{-10}, 2×10^{-6}),<br>2008-9: 8×10^{-5} (5×10^{-5}, 1×10^{-4}),<br><br>Influenza A/H3N2<br>2005-6: 0.001 (4×10^{-4}, 0.002)<br>2006-7: 9×10^{-7} (5×10^{-8}, 3×10^{-6})<br>2007-8: 5×10^{-5} (3×10^{-5}, 7×10^{-5})<br>2008-9: 2×10^{-4} (2×10^{-4}, 3×10^{-4})<br><br>Influenza B<br>2005-6: 5×10^{-5} (3×10^{-5}, 9×10^{-5}),<br>2006-7: 2×10^{-5} (4×10^{-6}, 5×10^{-5}),<br>2007-8: 2×10^{-6} (1×10^{-6}, 4×10^{-6}),<br>2008-9: 1×10^{-4} (7×10^{-5}, 2×10^{-4}),
Sensitivity of laboratory confirmation test	0.35 (0.25, 0.46)
Probability of symptoms given infection	0.67 (0.58, 0.75)

S18 Table. Model outcomes showing mean (95% CrI) under sensitivity analysis 6.

Total cost (million int $)	Outcomes (1000s)				Comparison with no childhood vaccination		
	Symptomatic infections	Outpatient visits	In-patient visits	Deaths	Incremental cost (million int $)	DALYs averted (1000s)	ICER
No childhood vaccination							
19 (16,24)	7871 (3936, 13124)	5.3 (0.0,7.3)	3.3 (1.2,8.0)	4.3 (0.6, 9.2)			
Policy 1: Vaccinate those aged 2-11 years with TIV							
237 (214, 286)	3547 (1105, 8963)	2.8 (0.0,7.2)	1.2 (0.5,5.4)	2.1 (0.3, 5.7)	218 (194, 267)	44 (0, 94)	4991
Policy 2: Vaccinate those aged 2-11 years with LAIV							
295 (273, 343)	2342 (452, 4929)	2.1 (0.0,4.9)	0.6 (0.3, 1.1)	1.5 (0.2, 3.5)	276 (252, 325)	56 (10, 109)	4914
Policy 3: Vaccinate those aged 2-17 years with LAIV							
392 (358, 450)	1854 (176, 4394)	1.8 (0.0,4.6)	0.5 (0.2, 0.8)	1.2 (0.0, 3.1)	373 (338, 431)	62 (12, 120)	6017
Policy 4: Vaccinate those aged 2-5 years with LAIV							
185 (175, 196)	3012 (1373, 5522)	2.5 (0.0,5.3)	1.0 (0.5, 2.2)	1,9 (0.4, 4.0)	166 (154, 178)	49 (8, 96)	3406
Policy 5: Vaccinate those aged 6-11 years with LAIV							
126 (108, 174)	4167 (1944, 7260)	3.1 (0.0,5.8)	1.4 (0.6, 3.8)	2.4 (0.4, 5.1)	107 (88, 154)	39 (6, 78)	2782
Policy 6: Vaccinate those aged 12-17 years with LAIV							
112 (95, 154)	3391 (713, 6523)	2.9 (0.0,5.7)	1.0 (0.5, 2.4)	1.9 (0.0, 4.7)	92 (73, 134)	47 (7, 97)	1976
Policy 7: Increase vaccination coverage in those aged 60 years and over with TIV							
84 (76, 95)	6335 (2936, 10868)	4.3 (0.6, 7.3)	2.6 (1.0, 7.5)	3.3 (0.6, 6.8)	66 (58, 74)	19 (0, 49)	3410

S42 Figure. Model fits to laboratory-confirmed influenza surveillance data under sensitivity analysis 6. Monthly numbers of PCR-confirmed influenza cases (circles) and model predictions: median (broken line), and 95%, 90% and 80% (gray shading) prediction intervals for the expected number of cases.

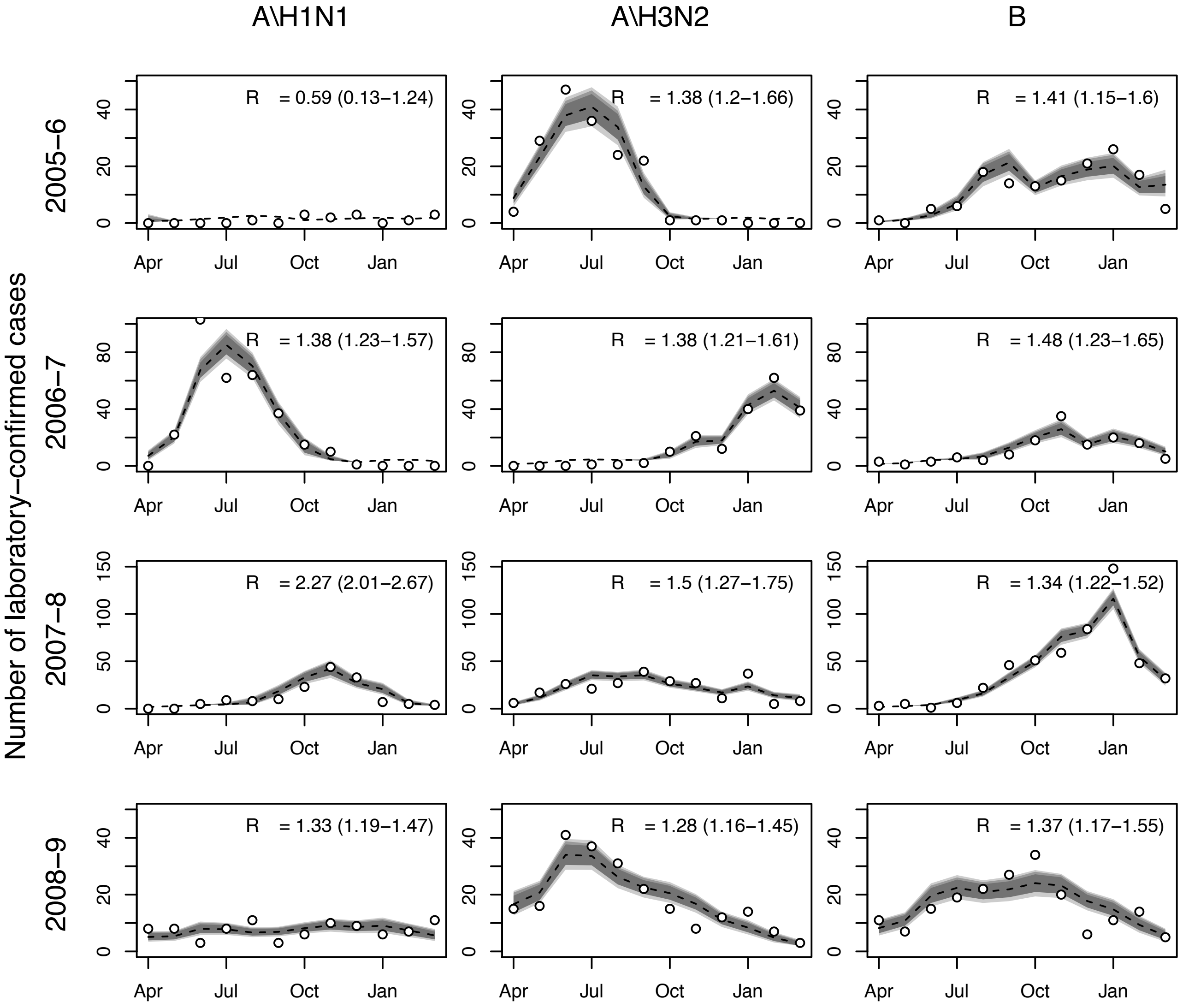

S43 Figure. Cost-effective Acceptability Curves under sensitivity analysis 6.

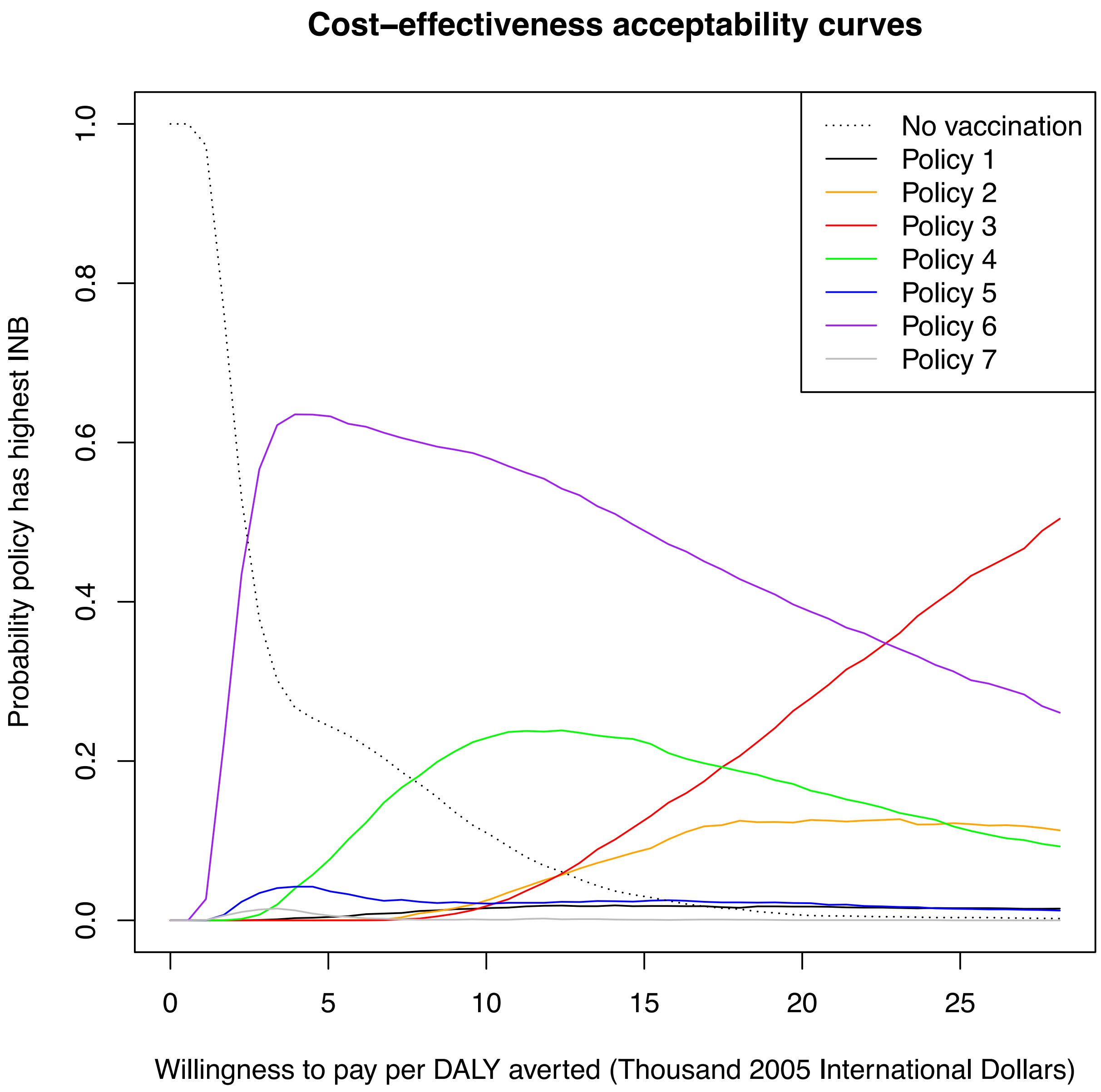

S44 Figure. Estimated numbers of symptomatic cases of seasonal influenza infection under sensitivity analysis 6.

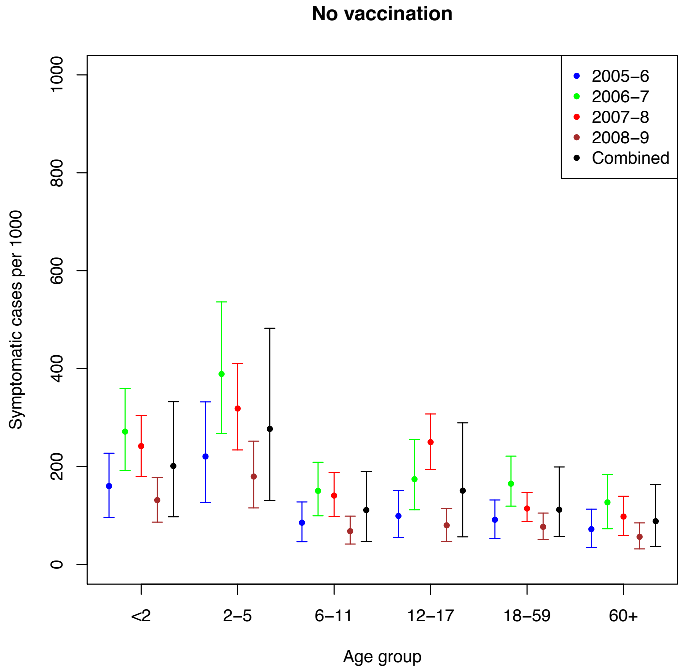

S45 Figure. Symptomatic cases averted under sensitivity analysis 6.

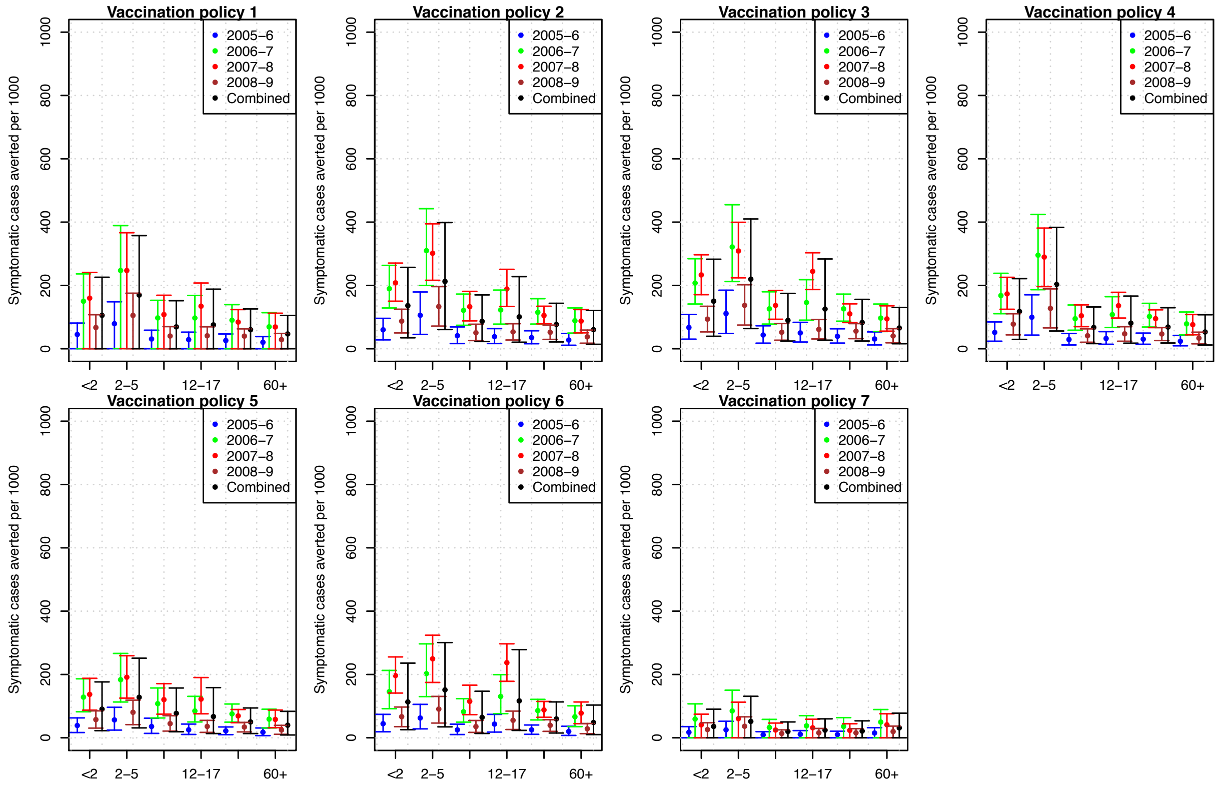

S46 Figure. Estimated number of deaths by age group due to influenza in the absence of vaccination against seasonal influenza under sensitivity analysis 6.

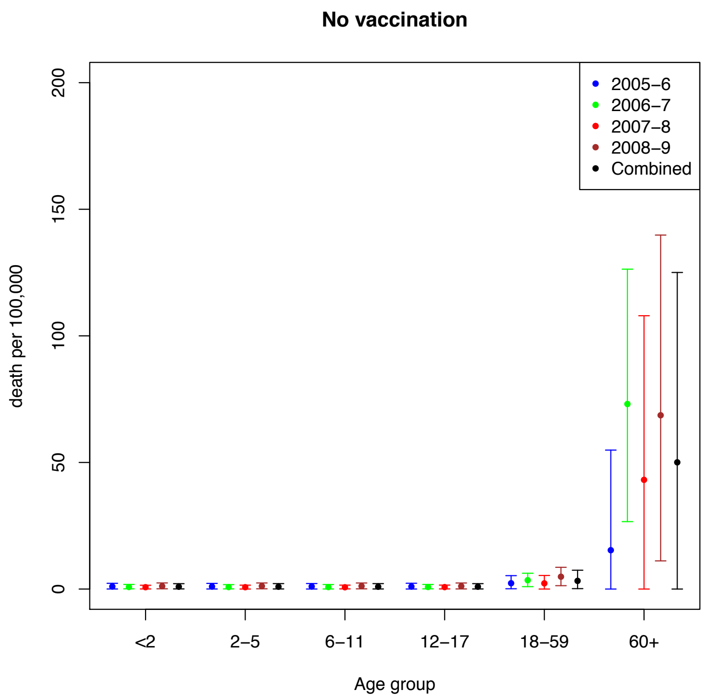

S47 Figure. Deaths averted under sensitivity analysis 6.

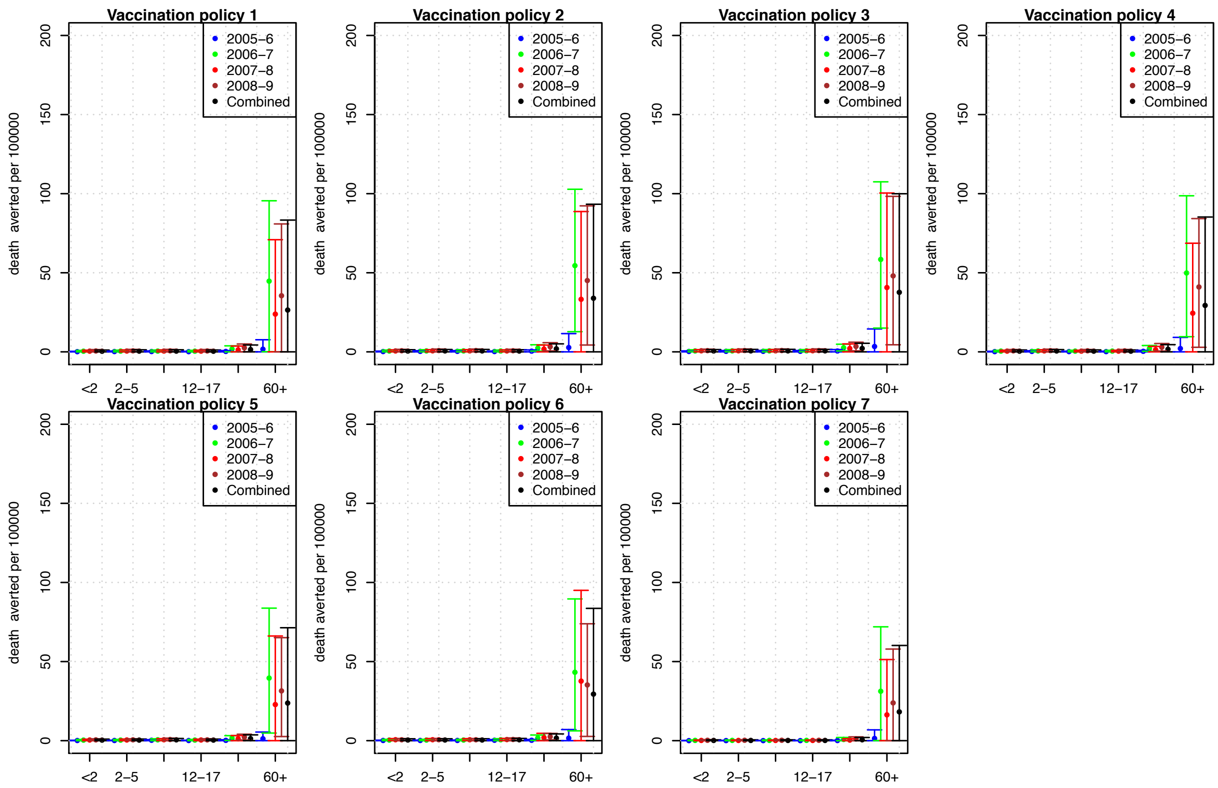

S48 Figure. DALYs averted as a result of direct and indirect vaccine effects under sensitivity analysis 6.

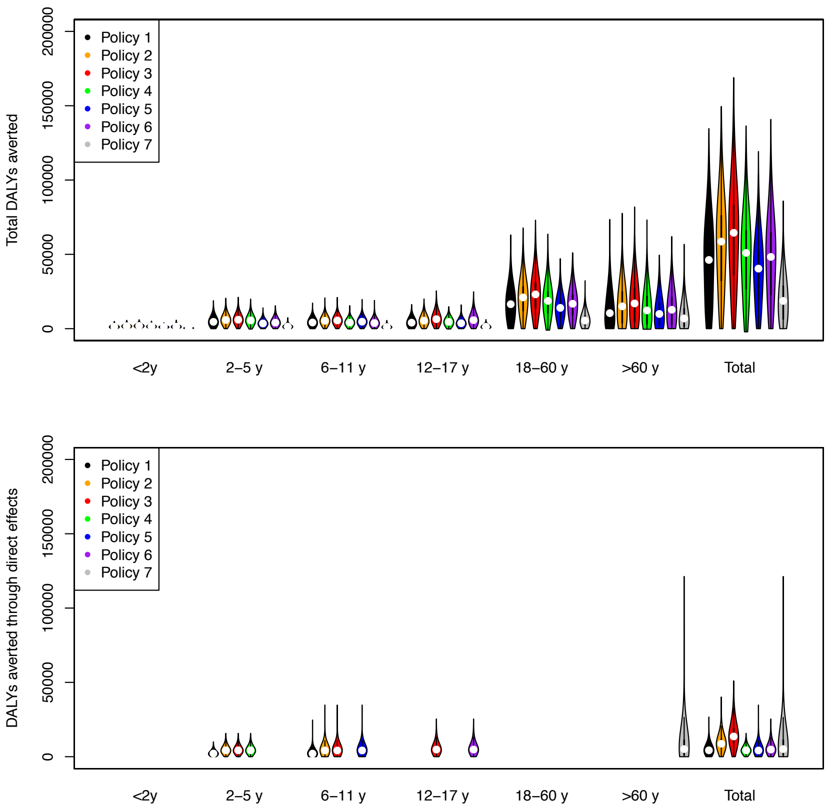

S49 Figure. Expected value of perfect information under sensitivity analysis 6.

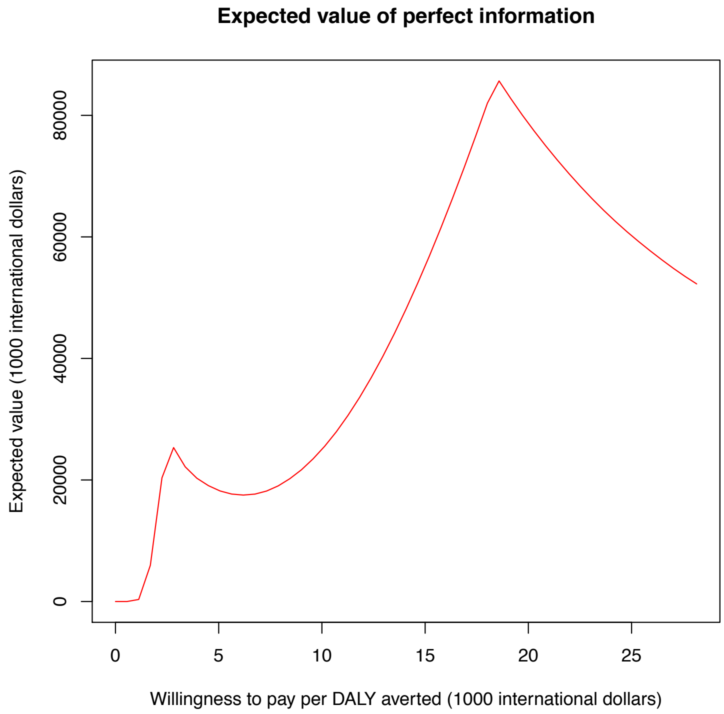

3.7 Additional results from sensitivity analysis 7

S19 Table. Posterior distributions from sensitivity analysis 7 (takes prior probability of immunity prior to start of each influenza season in age groups 2-17 as three times the value in the baseline scenario).

Parameter	Posterior mean (95% CrI)
R_0 for influenza A/ H1N1	2005-6: 0.57 (0.12, 1.20)<br>2006-7: 1.39 (1.30, 1.51)<br>2007-8: 1.40 (1.29, 1.50)<br>2008-9: 1.27 (1.21 1.35)
R_0 for influenza A/ H3N2	2005-6: 1.35 (1.25, 1.47)<br>2006-7: 1.36 (1.28, 1.45)<br>2007-8: 1.31 (1.24, 1.40)<br>2008-9: 1.31 (1.22, 1.42)
R_0 for influenza B	2005-6: 1.31 (1.23, 1.42)<br>2006-7: 1.36 (1.28, 1.48)<br>2007-8: 1.32 (1.27, 1.39)<br>2008-9: 1.32 (1.23, 1.42)
Serial interval influenza A/H1N1	2005-6: 2.50 (2.31, 2.70)<br>2006-7: 2.52 (2.34, 2.73)<br>2007-8: 2.37 (2.17, 2.57)<br>2008-9: 2.52 (2.33 2.72)
Serial interval influenza A/H3N2	2005-6: 2.51 (2.33, 2.72)<br>2006-7: 2.52 (2.32, 2.70)<br>2007-8: 2.51 (2.32, 2.72)<br>2008-9: 2.53 (2.31, 2.70)
Serial interval influenza B	2005-6: 2.50 (2.31, 2.70)<br>2006-7: 2.48 (2.30, 2.67)<br>2007-8: 2.64 (2.44, 2.86)<br>2008-9: 2.48 (2.28, 2.69)
Probability of immunity at the start of influenza season	
< 2 y	A/H1N1 2005-9: 0.10 (0.05, 0.17)<br>A/H3N2 2005-9: 0.10 (0.05, 0.17)<br>B 2005-9: 0.10 (0.05, 0.17)
2-5 y	A/H1N1 2005-9: 0.43 (0.20, 0.84)<br>A/H3N2 2005-9: 0.39 (0.20, 0.62)<br>B 2005-9: 0.39 (0.20, 0.65)
6-11 y	A/H1N1 2005-9: 0.50 (0.43, 0.58)<br>A/H3N2 2005-9: 0.50 (0.43, 0.57)<br>B 2005-9: 0.50 (0.43, 0.57)
12-17 y	A/H1N1 2005-9: 0.50 (0.43, 0.57)<br>A/H3N2 2005-9: 0.50 (0.43, 0.57)<br>B 2005-9: 0.50 (0.43, 0.57)
18-59 y	A/H1N1 2005-9: 0.20 (0.14, 0.27)<br>A/H3N2 2005-9: 0.19 (0.13, 0.26)<br>B 2005-9: 0.20 (0.14, 0.27)

Parameter	Posterior mean (95% CrI)
≥60 y	A/H1N1 2005-9: 0.19 (0.10, 0.30)<br>A/H3N2 2005-9: 0.19 (0.10, 0.30)<br>B 2005-9: 0.19 (0.10, 0.29)
Proportion initially infected	Influenza A/H1N1<br>2005-6: 0.001 (1×10^{-7}, 0.006)<br>2006-7: 1×10^{-4} (5×10^{-5}, 2×10^{-4}),<br>2007-8: 2×10^{-5} (1×10^{-5}, $3x10^{-5}$),<br>2008-9: 8×10^{-5} (5×10^{-5}, 1×10^{-4}),<br><br>Influenza A/H3N2<br>2005-6: 0.001 (4×10^{-4}, 0.002)<br>2006-7: 8×10^{-7} (3×10^{-8}, 2×10^{-6})<br>2007-8: 7×10^{-5} (5×10^{-5}, 9×10^{-5})<br>2008-9: 2×10^{-4} (2×10^{-4}, 3×10^{-4})<br><br>Influenza B<br>2005-6: 6×10^{-5} (3×10^{-5}, 9×10^{-5}),<br>2006-7: 2×10^{-5} (7×10^{-6}, 5×10^{-5}),<br>2007-8: 3×10^{-6} (1×10^{-6}, 5×10^{-6}),<br>2008-9: 1×10^{-4} (8×10^{-5}, 2×10^{-4}),
Sensitivity of laboratory confirmation test	0.34 (0.25, 0.45)
Probability of symptoms given infection	0.68 (0.59, 0.76)

S20 Table. Model outcomes showing mean (95% CrI) under sensitivity analysis 7.

	Outcomes (1000s)				Comparison with no childhood vaccination		
Total cost (million int $)	Symptomatic infections	Outpatient visits	In-patient visits	Deaths	Incremental cost (million int $)	DALYs averted (1000s)	ICER
No childhood vaccination							
19 (16,24)	7663 (3818, 12710)	5.3 (0.0,7.3)	3.3 (1.2,8.0)	4.3 (0.7, 9.2)			
Policy 1: Vaccinate those aged 2-11 years with TIV							
237 (215, 288)	3689 (860, 9086)	3.1 (0.0,7.3)	1.2 (0.5,4.6)	2.2 (0.3, 5.7)	219 (195, 269)	41 (0, 91)	5299
Policy 2: Vaccinate those aged 2-11 years with LAIV							
296 (273, 346)	2465 (521, 5044)	2.3 (0.0,5.0)	0.7 (0.4, 0.9)	1.6 (0.2, 3.7)	277 (253, 327)	54 (10, 104)	5142
Policy 3: Vaccinate those aged 2-17 years with LAIV							
392 (359, 453)	2136 (402, 4631)	2.0 (0.0,4.8)	0.6 (0.3, 0.8)	1.4 (0.1, 3.4)	373 (340, 435)	58 (11, 110)	6472
Policy 4: Vaccinate those aged 2-5 years with LAIV							
185 (175, 196)	2910 (747, 5562)	2.6 (0.0,5.4)	0.9 (0.5, 1.8)	1,8 (0.3, 4.1)	166 (154, 178)	49 (8, 96)	3398
Policy 5: Vaccinate those aged 6-11 years with LAIV							
127 (109, 178)	4995 (2462, 8385)	3.6 (0.0,6.1)	1.8 (0.8, 4.3)	2.7 (0.5, 5.9)	108 (90, 159)	29 (5, 58)	3683
Policy 6: Vaccinate those aged 12-17 years with LAIV							
112 (96, 153)	4423 (2178, 7512)	3.4 (0.0,6.0)	1.6 (0.7, 3.7)	2.4 (0.4, 5.4)	93 (76, 137)	35 (5, 69)	2650
Policy 7: Increase vaccination coverage in those aged 60 years and over with TIV							
84 (76, 93)	5500 (2564, 10106)	4.1 (0.0, 7.3)	2.2 (0.9, 6.7)	2.7 (0.5, 6.3)	65 (56, 74)	26 (0, 61)	2516

S50 Figure. Model fits to laboratory-confirmed influenza surveillance data under sensitivity analysis 7. Monthly numbers of PCR-confirmed influenza cases (circles) and model predictions: median (broken line), and 95%, 90% and 80% (gray shading) prediction intervals for the expected number of cases.

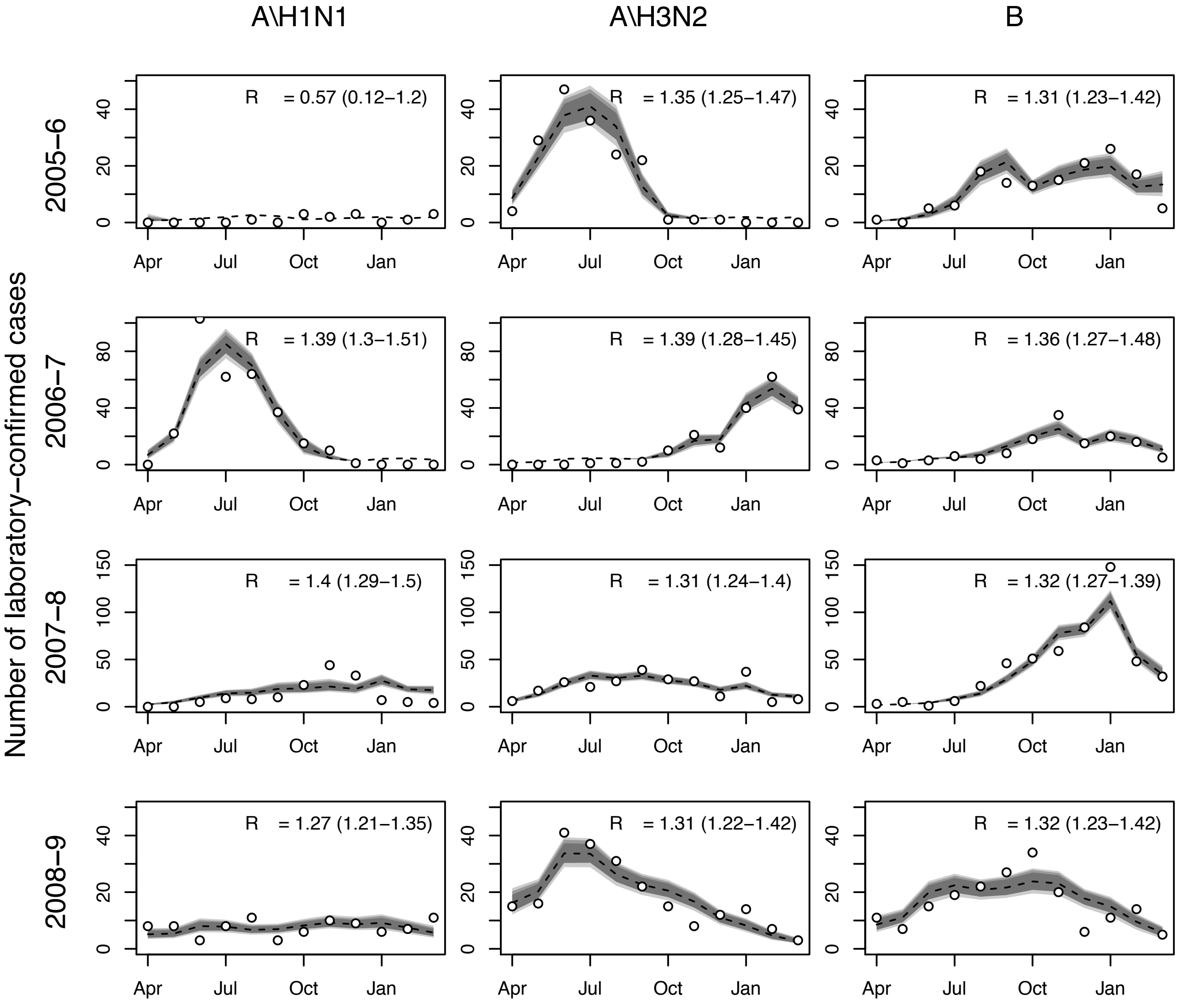

S51 Figure. Cost-effective Acceptability Curves under sensitivity analysis 7.

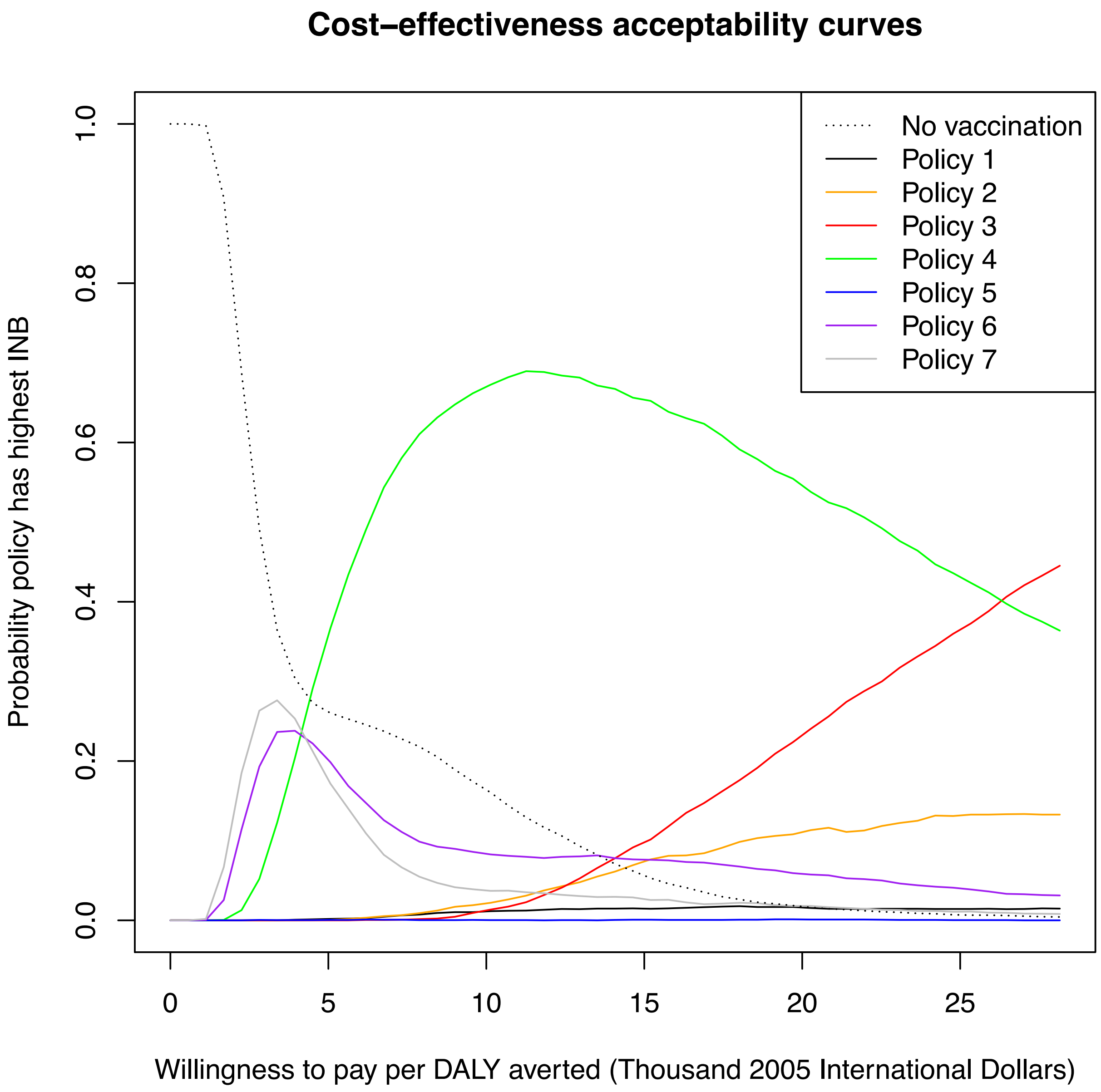

S52 Figure. Estimated numbers of symptomatic cases of seasonal influenza infection under sensitivity analysis 7.

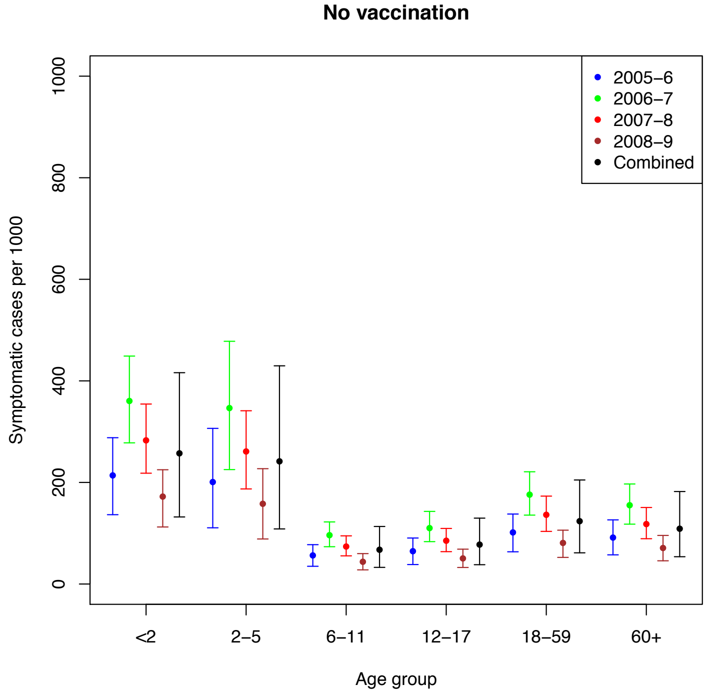

S53 Figure. Symptomatic cases averted under sensitivity analysis 7.

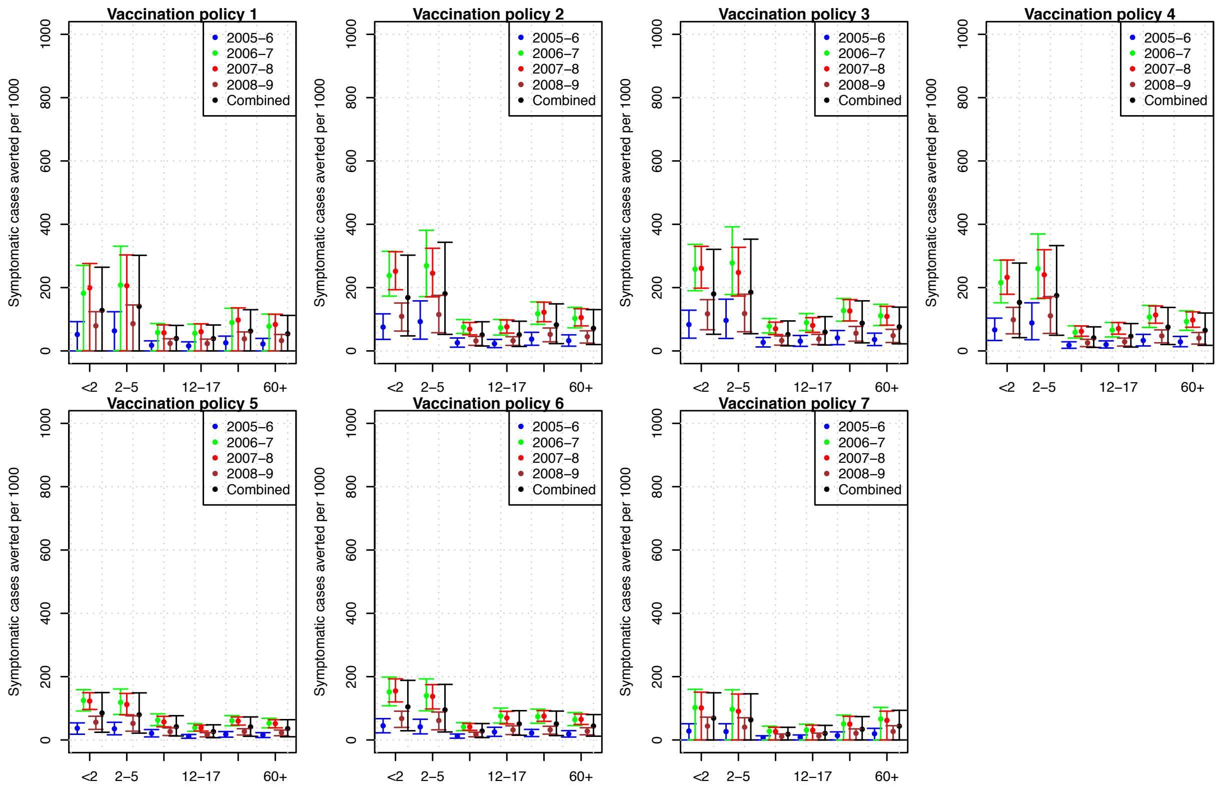

S54 Figure. Estimated number of deaths by age group due to influenza in the absence of vaccination against seasonal influenza under sensitivity analysis 7.

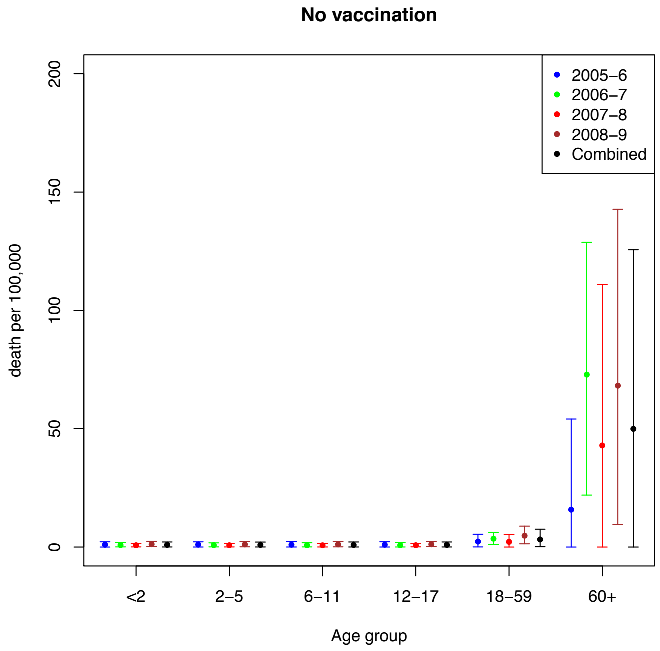

S55 Figure. Deaths averted under sensitivity analysis 7.

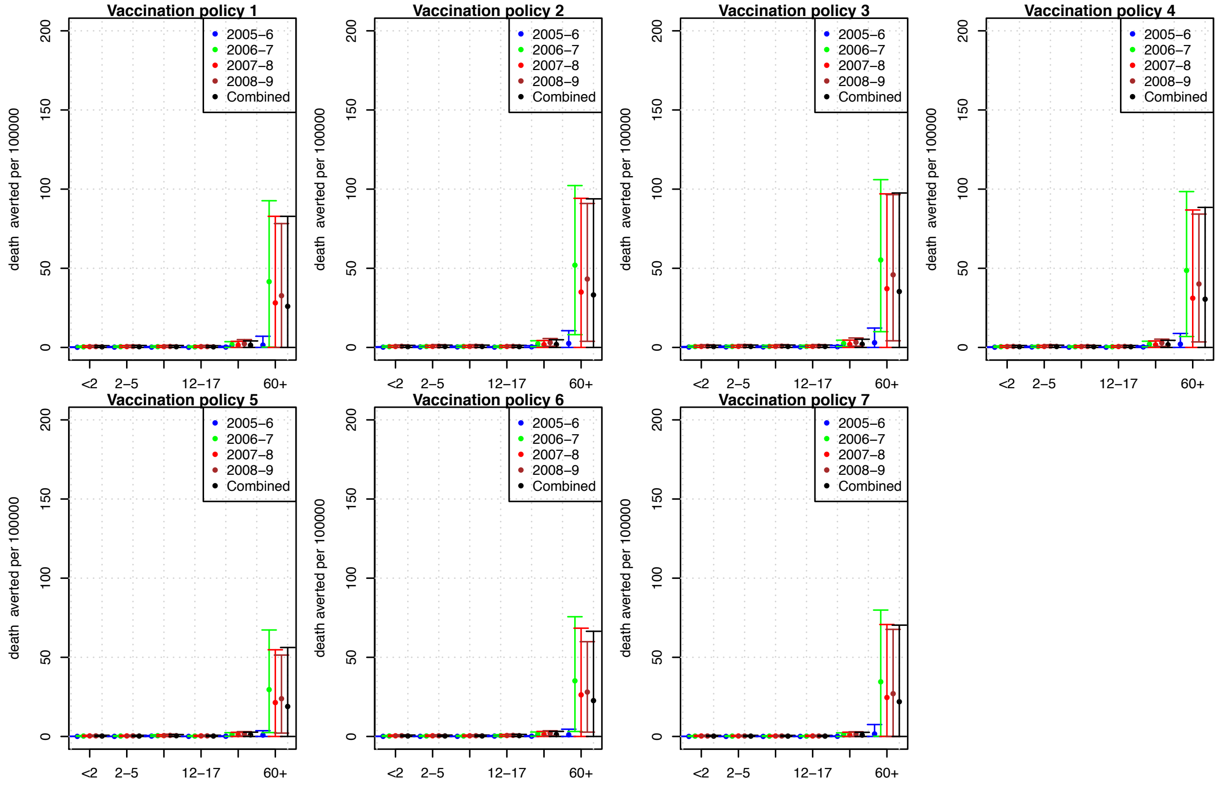

S56 Figure. DALYs averted as a result of direct and indirect vaccine effects under sensitivity analysis 7.

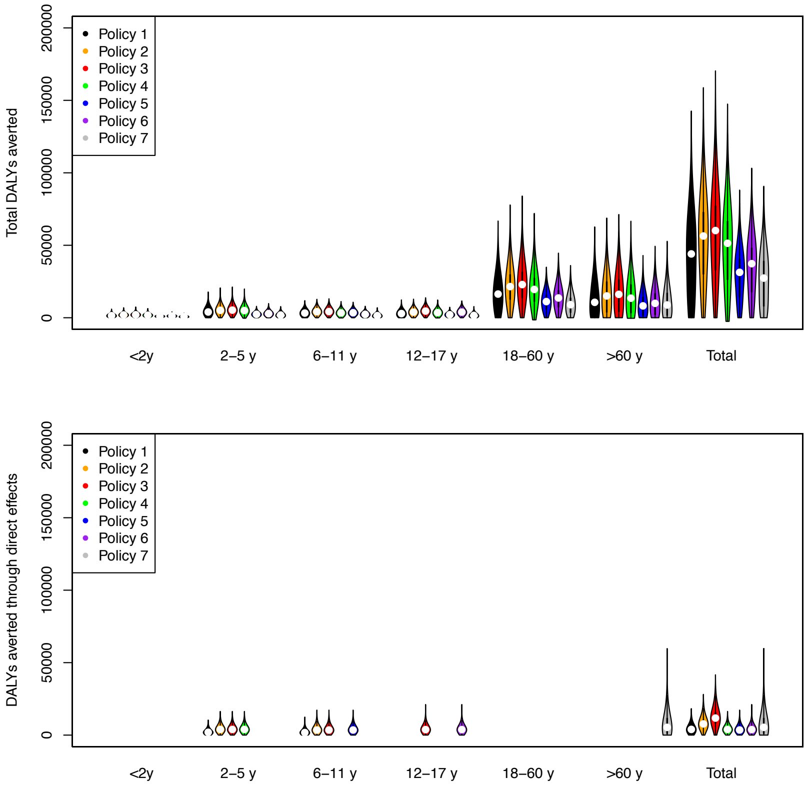

S57 Figure. Expected value of perfect information under sensitivity analysis 7.

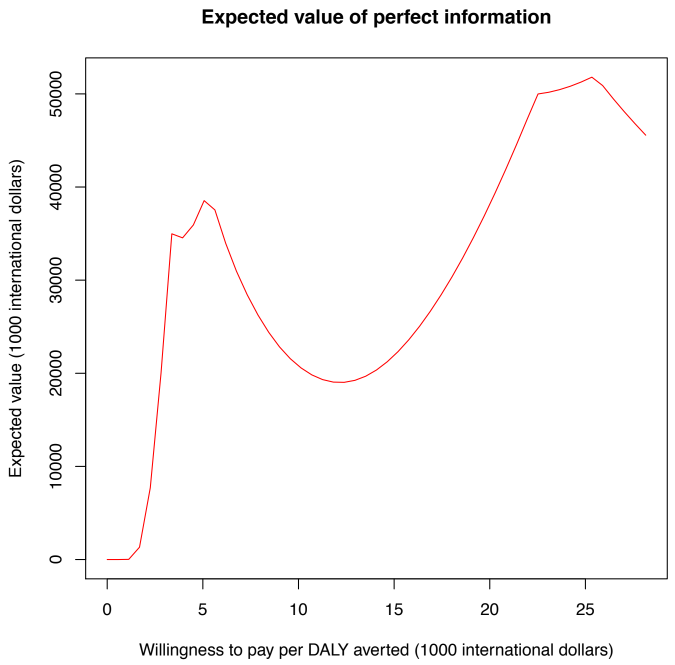

3.8 Additional results from sensitivity analysis 8

S21 Table. Posterior distributions from sensitivity analysis 8 (prior probability that an infection is symptomatic is taken as one quarter of the value in the base case analysis).

Parameter	Posterior mean (95% CrI)
R_0 for influenza A/ H1N1	2005-6: 0.49 (0.12, 1.02)<br>2006-7: 1.29 (1.23, 1.36)<br>2007-8: 1.15 (1.11, 1.20)<br>2008-9: 1.13 (1.08 1.19)
R_0 for influenza A/ H3N2	2005-6: 1.16 (1.05, 1.26)<br>2006-7: 1.18 (1.14, 1.24)<br>2007-8: 1.15 (1.11, 1.18)<br>2008-9: 1.16 (1.11, 1.21)
R_0 for influenza B	2005-6: 1.12 (1.07, 1.18)<br>2006-7: 1.15 (1.10, 1.23)<br>2007-8: 1.18 (1.13, 1.25)<br>2008-9: 1.17 (1.11, 1.23)
Serial interval influenza A/H1N1	2005-6: 2.50 (2.31, 2.69)<br>2006-7: 2.53 (2.34, 2.74)<br>2007-8: 2.48 (2.29, 2.67)<br>2008-9: 2.50 (2.32 2.70)
Serial interval influenza A/H3N2	2005-6: 2.51 (2.33, 2.71)<br>2006-7: 2.51 (2.32, 2.70)<br>2007-8: 2.51 (2.33, 2.71)<br>2008-9: 2.55 (2.36, 2.75)
Serial interval influenza B	2005-6: 2.50 (2.32, 2.70)<br>2006-7: 2.49 (2.30, 2.68)<br>2007-8: 2.53 (2.35, 2.74)<br>2008-9: 2.48 (2.29, 2.68)
Probability of immunity at the start of influenza season	
< 2 y	A/H1N1 2005-9: 0.10 (0.05, 0.18)<br>A/H3N2 2005-9: 0.10 (0.05, 0.17)<br>B 2005-9: 0.10 (0.05, 0.17)
2-5 y	A/H1N1 2005-9: 0.13 (0.06, 0.21)<br>A/H3N2 2005-9: 0.13 (0.07, 0.21)<br>B 2005-9: 0.13 (0.06, 0.21)
6-11 y	A/H1N1 2005-9: 0.17 (0.14, 0.19)<br>A/H3N2 2005-9: 0.17 (0.14, 0.19)<br>B 2005-9: 0.17 (0.14, 0.19)
12-17 y	A/H1N1 2005-9: 0.17 (0.14, 0.19)<br>A/H3N2 2005-9: 0.17 (0.14, 0.19)<br>B 2005-9: 0.17 (0.14, 0.19)
18-59 y	A/H1N1 2005-9: 0.19 (0.13, 0.26)<br>A/H3N2 2005-9: 0.19 (0.13, 0.25)<br>B 2005-9: 0.19 (0.13, 0.27)

Parameter	Posterior mean (95% CrI)
≥60 y	A/H1N1 2005-9: 0.19 (0.09, 0.30)<br>A/H3N2 2005-9: 0.19 (0.10, 0.29)<br>B 2005-9: 0.19 (0.10, 0.30)
Proportion initially infected	Influenza A/H1N1<br>2005-6: 0.002 (3×10^{-7}, 0.01)<br>2006-7: 3×10^{-5} (1×10^{-7}, 9×10^{-5}),<br>2007-8: 4×10^{-6} (2×10^{-6}, $7x10^{-6}$),<br>2008-9: 2×10^{-4} (1×10^{-4}, 2×10^{-4}),<br><br>Influenza A/H3N2<br>2005-6: 0.002 (5×10^{-3}, 0.003)<br>2006-7: 8×10^{-7} (3×10^{-9}, 3×10^{-6})<br>2007-8: 2×10^{-5} (1×10^{-5}, 3×10^{-5})<br>2008-9: 3×10^{-4} (2×10^{-4}, 5×10^{-4})<br><br>Influenza B<br>2005-6: 9×10^{-5} (1×10^{-5}, 2×10^{-4}),<br>2006-7: 4×10^{-5} (1×10^{-5}, 8×10^{-5}),<br>2007-8: 1×10^{-6} (5×10^{-7}, 2×10^{-6}),<br>2008-9: 1×10^{-4} (8×10^{-5}, 3×10^{-4}),
Sensitivity of laboratory confirmation test	0.25 (0.18, 0.34)
Probability of symptoms given infection	0.17 (0.14, 0.19)

S22 Table. Model outcomes showing mean (95% CrI) under sensitivity analysis 8.

Total cost (million int $)	Outcomes (1000s) Symptomatic infections	Outpatient visits	In-patient visits	Deaths	Comparison with no childhood vaccination Incremental cost (million int $)	DALYs averted (1000s)	ICER
No childhood vaccination							
19<br>(16,24)	7663<br>(3818, 12710)	5.3<br>(0.0,7.3)	3.3<br>(1.2,8.0)	4.3<br>(0.7, 9.2)			
Policy 1: Vaccinate those aged 2-11 years with TIV							
237<br>(215, 288)	3689<br>(860, 9086)	3.1<br>(0.0,7.3)	1.2<br>(0.5,4.6)	2.2<br>(0.3, 5.7)	219<br>(195, 269)	41<br>(0, 91)	5299
Policy 2: Vaccinate those aged 2-11 years with LAIV							
296<br>(273, 346)	2465<br>(521, 5044)	2.3<br>(0.0,5.0)	0.7<br>(0.4, 0.9)	1.6<br>(0.2, 3.7)	277<br>(253, 327)	54<br>(10, 104)	5142
Policy 3: Vaccinate those aged 2-17 years with LAIV							
392<br>(359, 453)	2136<br>(402, 4631)	2.0<br>(0.0,4.8)	0.6<br>(0.3, 0.8)	1.4<br>(0.1, 3.4)	373<br>(340, 435)	58<br>(11, 110)	6472
Policy 4: Vaccinate those aged 2-5 years with LAIV							
185<br>(175, 196)	2910<br>(747, 5562)	2.6<br>(0.0,5.4)	0.9<br>(0.5, 1.8)	1,8<br>(0.3, 4.1)	166<br>(154, 178)	49<br>(8, 96)	3398
Policy 5: Vaccinate those aged 6-11 years with LAIV							
127<br>(109, 178)	4995<br>(2462, 8385)	3.6<br>(0.0,6.1)	1.8<br>(0.8, 4.3)	2.7<br>(0.5, 5.9)	108<br>(90, 159)	29<br>(5, 58)	3683
Policy 6: Vaccinate those aged 12-17 years with LAIV							
112<br>(96, 153)	4423<br>(2178, 7512)	3.4<br>(0.0,6.0)	1.6<br>(0.7, 3.7)	2.4<br>(0.4, 5.4)	93<br>(76, 137)	35<br>(5, 69)	2650
Policy 7: Increase vaccination coverage in those aged 60 years and over with TIV							
84<br>(76, 93)	5500<br>(2564, 10106)	4.1<br>(0.0, 7.3)	2.2<br>(0.9, 6.7)	2.7<br>(0.5, 6.3)	65<br>(56, 74)	26<br>(0, 61)	2516

S58 Figure. Model fits to laboratory-confirmed influenza surveillance data under sensitivity analysis 7. Monthly numbers of PCR-confirmed influenza cases (circles) and model predictions: median (broken line), and 95%, 90% and 80% (gray shading) prediction intervals for the expected number of cases.

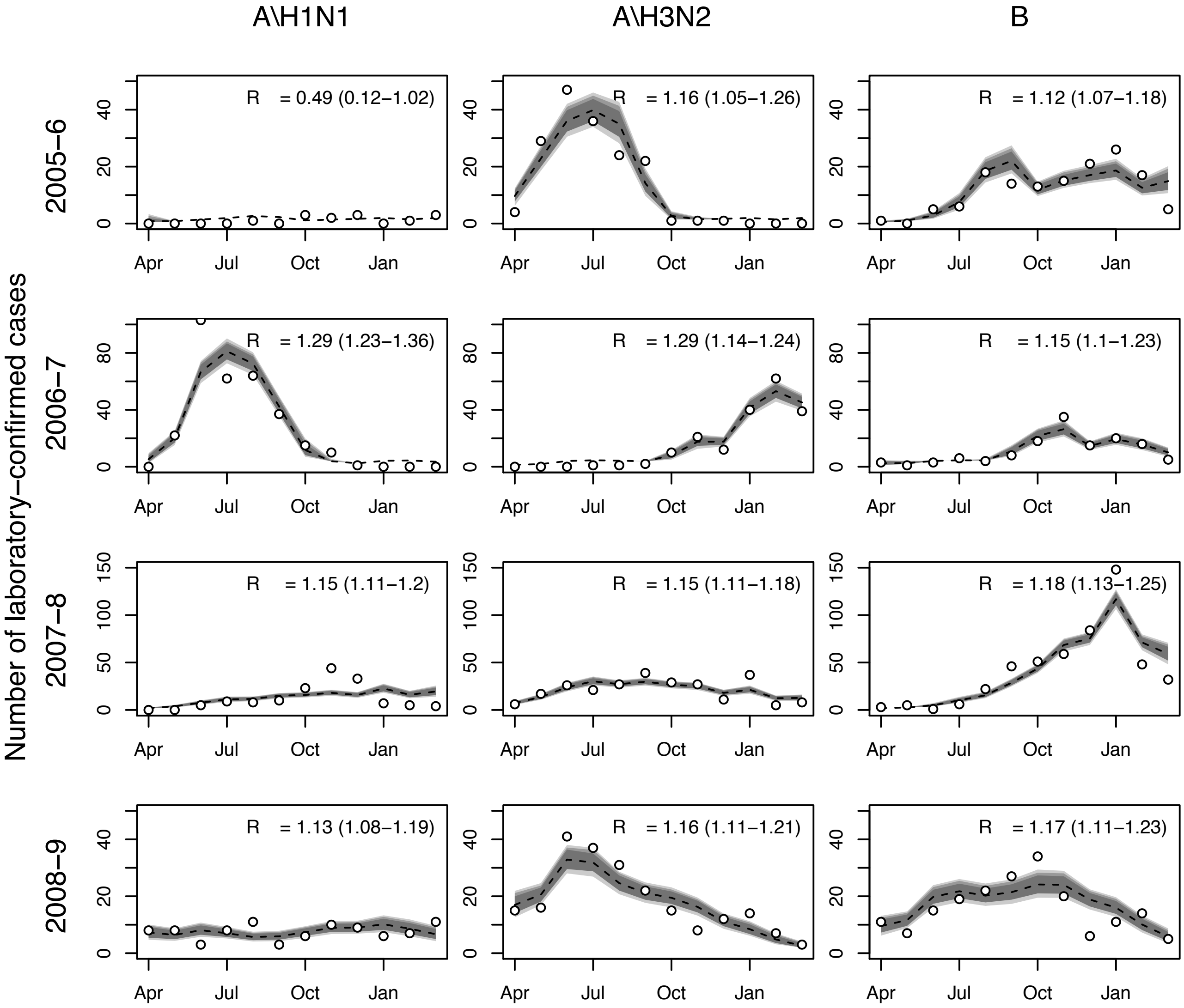

S59 Figure. Cost-effective Acceptability Curves under sensitivity analysis 8.

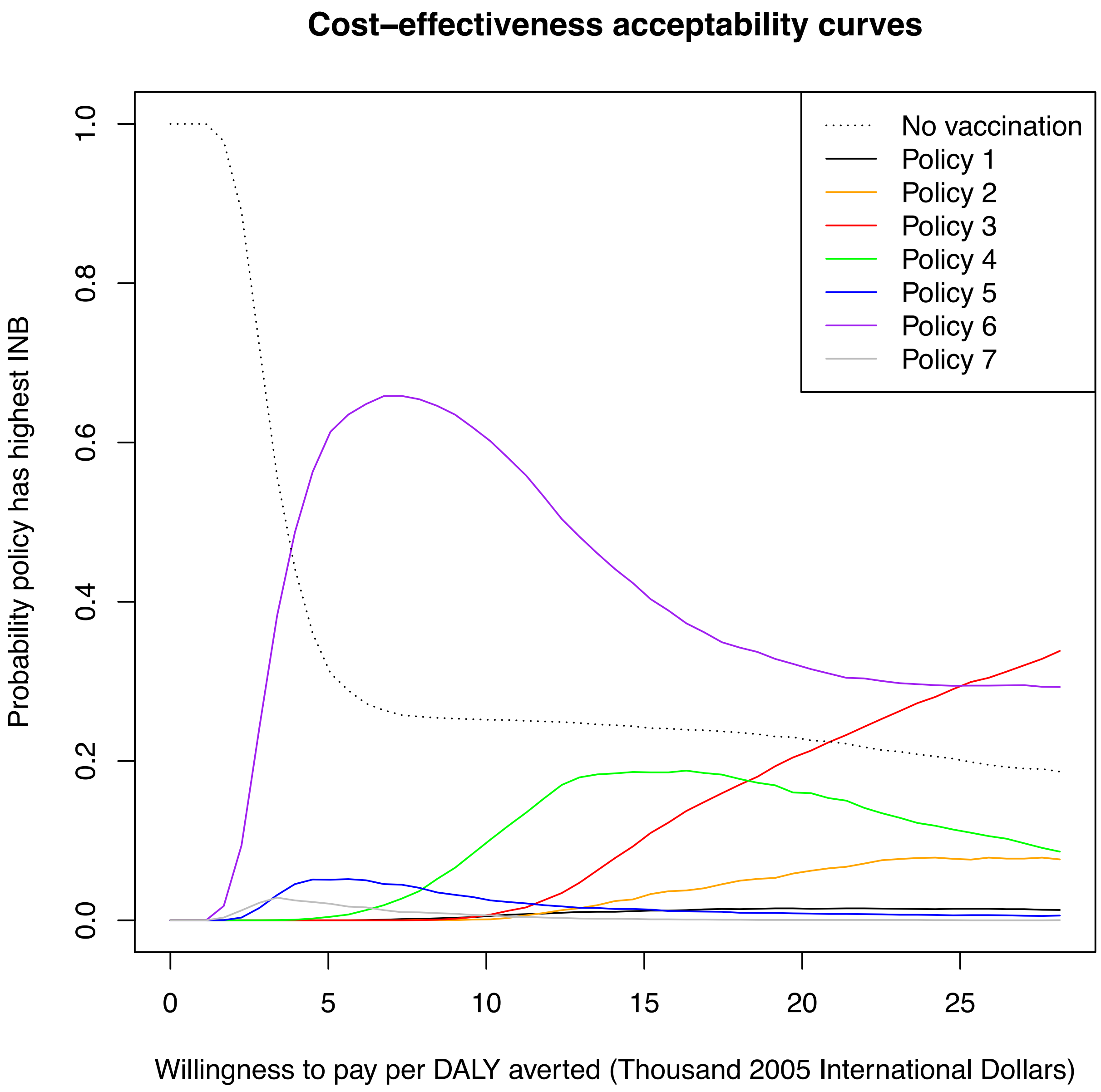

S60 Figure. Estimated numbers of symptomatic cases of seasonal influenza infection under sensitivity analysis 8.

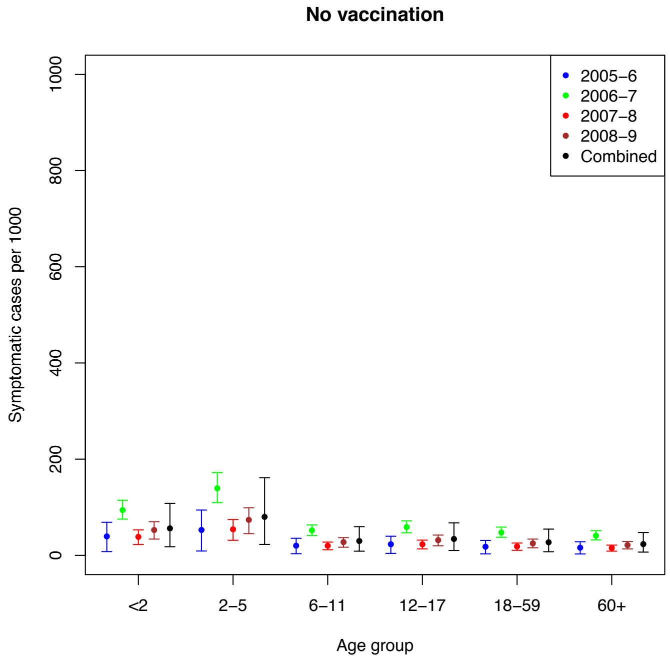

S61 Figure. Symptomatic cases averted under sensitivity analysis 8.

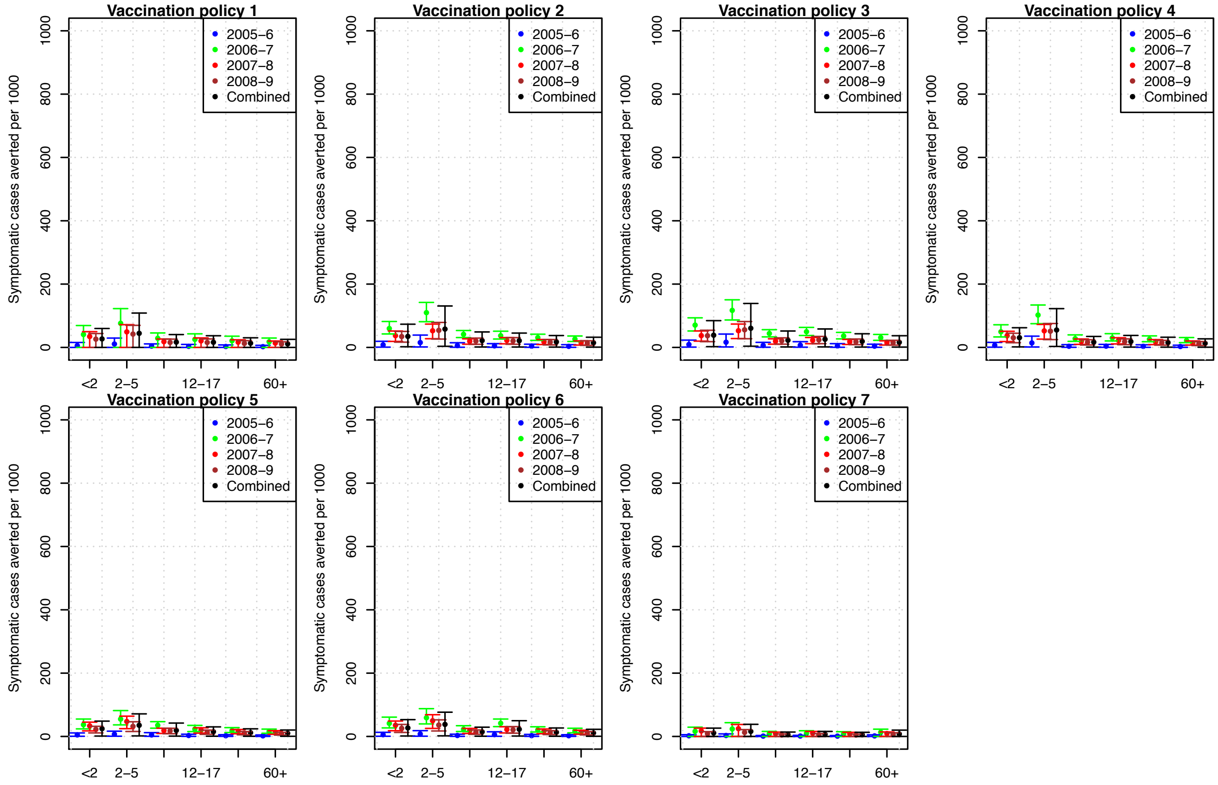

S62 Figure. Estimated number of deaths by age group due to influenza in the absence of vaccination against seasonal influenza under sensitivity analysis 8.

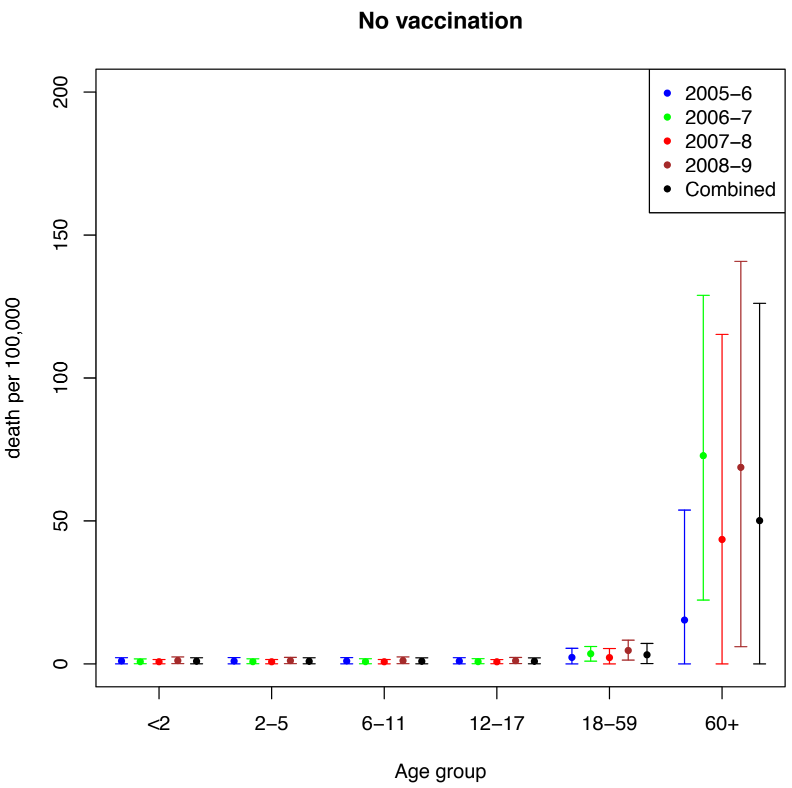

S63 Figure. Deaths averted under sensitivity analysis 8.

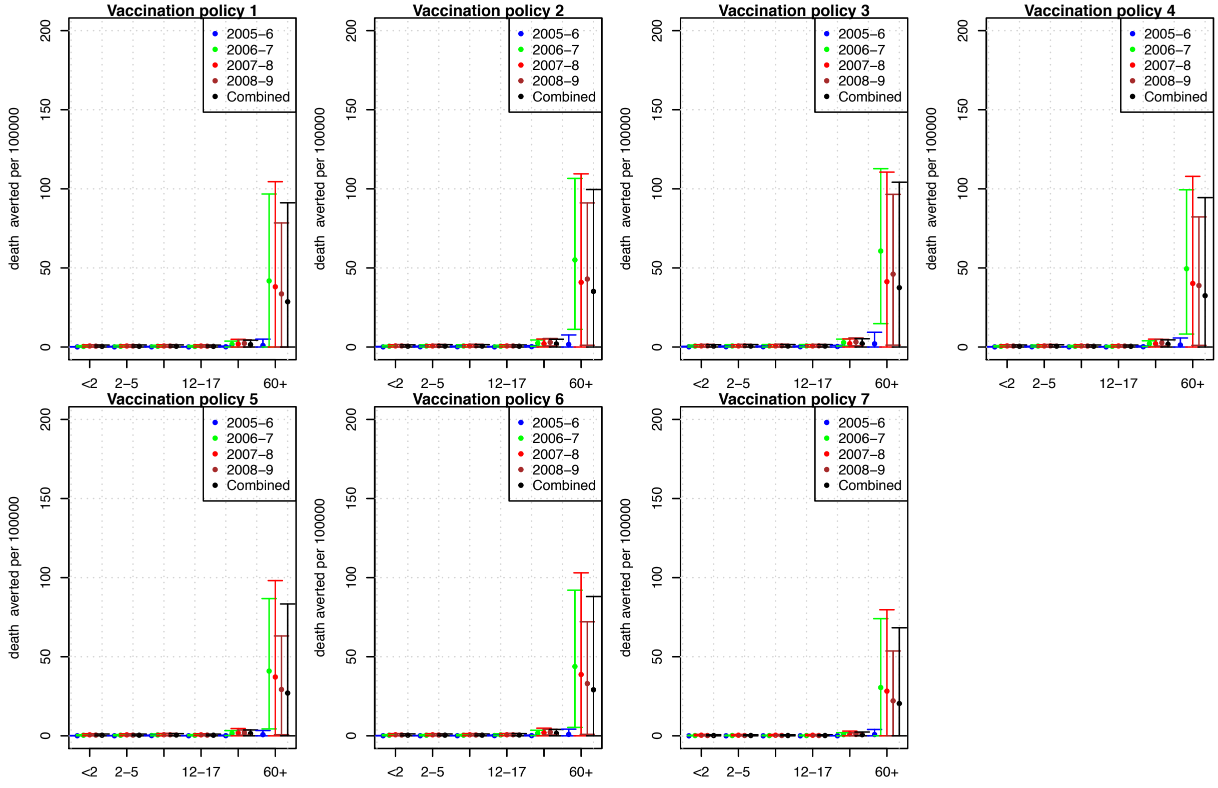

S64 Figure. DALYs averted as a result of direct and indirect vaccine effects under sensitivity analysis 8.

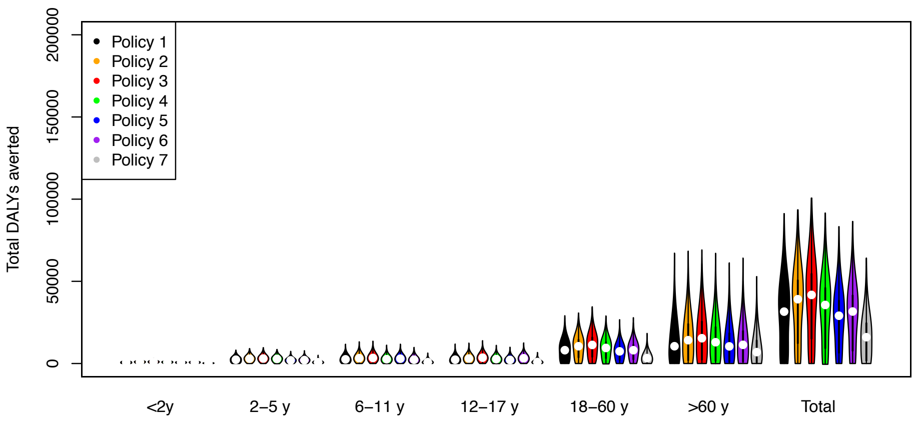

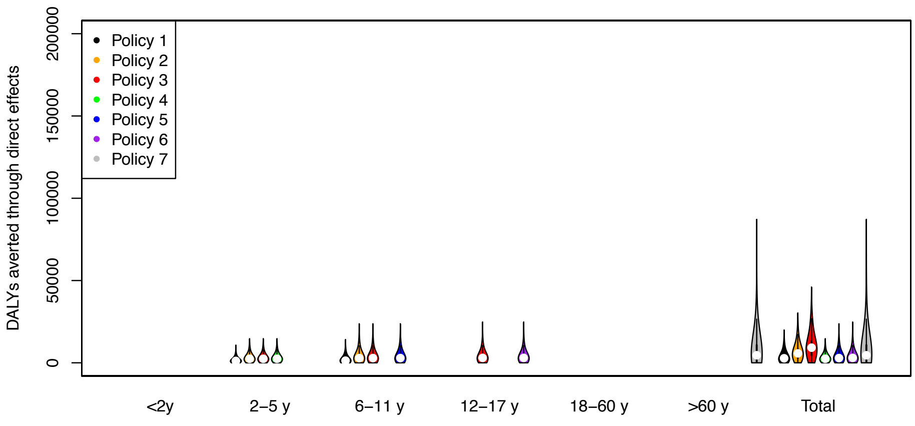

S65 Figure. Expected value of perfect information under sensitivity analysis 8.

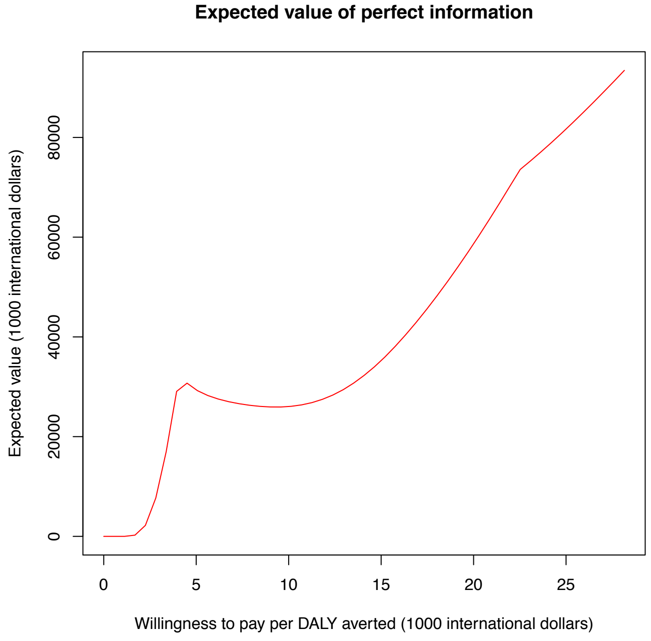

4. References

Loeb M, Russell ML, Moss L, Fonseca K, Fox J, et al. (2010) Effect of influenza vaccination of children on infection rates in Hutterite communities: a randomized trial. *JAMA* 303: 943-950.

Clover RD, Crawford S, Glezen WP, Taber LH, Matson CC, Couch RB (1991) Comparison of heterotypic protection against influenza A/Taiwan/86 (H1N1) by attenuated and inactivated vaccines to A/Chile/83-like viruses. *J Infect Dis* 163:300-4.

Belshe RB, Mendelman PM, Treanor J, King J, Gruber WC, et al. (1998) The efficacy of live attenuated, cold-adapted, trivalent, intranasal influenza virus vaccine in children. *N Engl J Med* 338: 1405-1412.

Belshe RB, Gruber WC, Mendelman PM, Cho I, Reisinger K, Block SL, Wittes J, Iacuzio D, Piedra P, Treanor J, King J, Kotloff K, Bernstein DI, Hayden FG, Zangwill K, Yan L, Wolff M (2000) Efficacy of vaccination with live attenuated, cold-adapted, trivalent, intranasal influenza virus vaccine against a variant (A/Sydney) not contained in the vaccine. *J Pediatr* 136:168-75.

Cowling BJ, Ng S, Ma ES, Fang VJ, So HC, Wai W, Cheng CK, Wong JY, Chan KH, Ip DK, Chiu SS, Peiris JS, Leung GM (2012) Protective efficacy against pandemic influenza of seasonal influenza vaccination in children in Hong Kong: a randomized controlled trial. *Clin Infect Dis* 55:695-702.

Klick B, Durrani S, Chan KH, Ip DK, Chou ES, Kwok HK, Ng S, Chiu SS, Peiris JS, Leung GM, Cowling BJ (2013) Live attenuated seasonal and pandemic influenza vaccine in school-age children: a randomized controlled trial. *Vaccine* 31:1937-43.

Tam JS, Capeding MR, Lum LC, Chotpitayasunondh T, Jiang Z, et al. (2007) Efficacy and safety of a live attenuated, cold-adapted influenza vaccine, trivalent against culture-confirmed influenza in young children in Asia. *Pediatr Infect Dis J* 26: 619-628.

Lum LC, Borja-Tabora CF, Breiman RF, Vesikari T, Sablan BP, et al. (2010) Influenza vaccine concurrently administered with a combination measles, mumps, and rubella vaccine to young children. *Vaccine* 28: 1566-1574.

Cooper BS, Kotirum S, Kulpeng W, Praditsitthikorn N, Chittagan- pitch M, Limmathurotsakul D, Day NP, Coker R, Teerawattananon Y, Meeyai A (2014). Mortality attributable to seasonal influenza A and B infections in Thailand, 2005-2009: a longitudinal study. American Journal of Epidemiology (in press).

Goldstein E, Viboud C, Charu V, Lipsitch M (2012) Improving the estimation of influenza-related mortality over a seasonal baseline. *Epidemiology* 23:829-38.

Simmerman JM, Lertiendumrong J, Dowell SF, Uyeki T, Olsen SJ, Chittaganpitch M, et al. (2006) The cost of influenza in Thailand. *Vaccine* 24:4417-26.

Dieleman J, Romio S, Johansen K, Weibel D, Bonhoeffer J, Sturkenboom M (2011). Guillain- Barre syndrome and adjuvanted pandemic influenza A (H1N1) 2009 vaccine: multinational case-control study in Europe. *BMJ* 343:d3908.

Stowe J, Andrews N, Wise L, Miller E (2009) Investigation of the temporal association of Guillain-Barre syndrome with influenza vaccine and influenzalike illness using the United Kingdom General Practice Research Database. *Am J Epidemiol* 169:382-8

Neuzil KM, Dupont WD, Wright PF, Edwards KM (2001) Efficacy of inactivated and cold-adapted vaccines against influenza A infection, 1985 to 1990: the pediatric experience. *Pediatr Infect Dis J* 20:733-40.

Prosser LA, Bridges CB, Uyeki TM, Rego VH, Ray GT, Meltzer MI, et al (2005) Values for preventing influenza-related morbidity and vaccine adverse events in children. *Health Qual Life Outcomes* 3:18.

Lugner AK, van Boven M, de Vries R, Postma MJ, Wallinga J (2012). Cost effectiveness of vaccination against pandemic influenza in European countries: mathematical modelling analysis. *BMJ* 345:e4445.

Riewpaiboon A. (2011) Standard cost lists for health technology assessment. Nonthaburi: Health Intervention and Technology Assessment Program (HITAP).

PATH WHO, Health Systems Research Institute, Mahidol University (2011) An assessment of vaccine supply chain and logistics systems in Thailand. Seattle.

R Development Core Team (2010) R: A language and environment for statistical computing. R Foundation for Statistical Computing, Vienna, Austria. ISBN 3-900051-07-0, URL http://www.R-project.org/.

Bowman A, Crawford E, Alexander G, Bowman RW (2007) rpanel: Simple Interactive Controls for R Functions Using the tcltk Package. *Journal of Statistical Software* 17, 1-18. URL http://www.jstatsoft.org/v17/i09/.

Baguelin M, Flasche S, Camacho A, Demiris N, Miller E, et al. (2013) Assessing optimal target populations for influenza vaccination programmes: an evidence synthesis and modelling study. PLoS Med 10: e1001527.

5. Model code

Code for running the model is available here http://goo.gl/htrvrk .
